# Supplementary material for: Scabies outbreak management in refugee/migrant camps in Europe 2014–2017: a retrospective qualitative interview study of healthcare staff experiences and perspectives
Source: BMJ Open. 2023 Nov 8;13(11):e075103. doi: 10.1136/bmjopen-2023-075103 (PMC10632829; doi:10.1136/bmjopen-2023-075103)
Supplement: Author's manuscript [file bmjopen-2023-075103.draft_revisions.pdf]

BMJ Open is committed to open peer review. As part of this commitment we make the peer review history of every article we publish publicly available.

When an article is published we post the peer reviewers' comments and the authors' responses online. We also post the versions of the paper that were used during peer review. These are the versions that the peer review comments apply to.

The versions of the paper that follow are the versions that were submitted during the peer review process. They are not the versions of record or the final published versions. They should not be cited or distributed as the published version of this manuscript.

BMJ Open is an open access journal and the full, final, typeset and author-corrected version of record of the manuscript is available on our site with no access controls, subscription charges or pay-per-view fees (http://bmjopen.bmj.com).

If you have any questions on BMJ Open's open peer review process please email info.bmjopen@bmj.com

Scabies outbreak management in refugee/migrant camps in Europe 2014-17: a retrospective qualitative interview study of healthcare staff experiences and perspectives

Journal:	*BMJ Open*
Manuscript ID	bmjopen-2023-075103
Article Type:	Original research
Date Submitted by the Author:	26-Apr-2023
Complete List of Authors:	Richardson, Naomi; Brighton and Sussex Medical School<br>Cassell, Jackie; Brighton and Sussex Medical School, Primary Care and Public Health; UK Health Security Agency<br>Head, Michael; University of Southampton, Clinical Informatics Research Unit, Faculty of Medicine<br>Lanza, Stefania; Brighton and Sussex Medical School<br>Schaefer, Corinna ; CADUS e.V<br>Walker, Stephen; London School of Hygiene & Tropical Medicine, Faculty of Infectious and Tropical Diseases; University College London Hospitals NHS Foundation Trust, Hospital for Tropical Diseases, and Department of Dermatology<br>Middleton, Jo; Brighton and Sussex Medical School, Primary Care and Public Health, and NIHR Global Health Research Unit on Neglected Tropical Diseases; University of Sussex, Ecology, Behaviour and Environment, School of Life Sciences
Keywords:	Infectious diseases & infestations < DERMATOLOGY, International health services < HEALTH SERVICES ADMINISTRATION & MANAGEMENT, Infection control < INFECTIOUS DISEASES, Mass Drug Administration, Neglected Diseases, QUALITATIVE RESEARCH

BMJ

I, the Submitting Author has the right to grant and does grant on behalf of all authors of the Work (as defined in the below author licence), an exclusive licence and/or a non-exclusive licence for contributions from authors who are: i) UK Crown employees; ii) where BMJ has agreed a CC-BY licence shall apply, and/or iii) in accordance with the terms applicable for US Federal Government officers or employees acting as part of their official duties; on a worldwide, perpetual, irrevocable, royalty-free basis to BMJ Publishing Group Ltd ("BMJ") its licensees and where the relevant Journal is co-owned by BMJ to the co-owners of the Journal, to publish the Work in this journal and any other BMJ products and to exploit all rights, as set out in our licence.

The Submitting Author accepts and understands that any supply made under these terms is made by BMJ to the Submitting Author unless you are acting as an employee on behalf of your employer or a postgraduate student of an affiliated institution which is paying any applicable article publishing charge ("APC") for Open Access articles. Where the Submitting Author wishes to make the Work available on an Open Access basis (and intends to pay the relevant APC), the terms of reuse of such Open Access shall be governed by a Creative Commons licence – details of these licences and which Creative Commons licence will apply to this Work are set out in our licence referred to above.

Other than as permitted in any relevant BMJ Author's Self Archiving Policies, I confirm this Work has not been accepted for publication elsewhere, is not being considered for publication elsewhere and does not duplicate material already published. I confirm all authors consent to publication of this Work and authorise the granting of this licence.

For peer review only

[3989/4000 words; 3 tables, two figures; online supplementary file]

Scabies outbreak management in refugee/migrant camps in Europe 2014–17: a retrospective qualitative interview study of healthcare staff experiences and perspectives

Naomi A Richardson[1], Jackie A. Cassell[1,2], Michael G Head[3], Stefania Lanza[1], Corinna Schaefer[4], Stephen L Walker[5,6], Jo Middleton[7,8,*]

[1] Department of Primary Care and Public Health, Brighton and Sussex Medical School, Falmer, UK
[2] UK Health Security Agency, London, UK
[3] Clinical Informatics Research Unit, Faculty of Medicine, University of Southampton, Southampton, UK
[4] CADUS e.V., Berlin, Germany
[5] Faculty of Infectious and Tropical Diseases, London School of Hygiene & Tropical Medicine, London, UK
[6] Hospital for Tropical Diseases and Department of Dermatology, University College London Hospitals NHS Foundation Trust, London, UK.
[7] NIHR Global Health Research Unit on Neglected Tropical Diseases and Department of Primary Care and Public Health, Brighton and Sussex Medical School, Falmer, UK
[8] Evolution, behaviour and environment, School of Life Sciences, University of Sussex, Falmer, UK

*Correspondence: Jo Middleton, Department of Primary Care and Public Health, Brighton and Sussex Medical School, Watson Building, Falmer, BN1 9PH, UK.
Email: j.middleton@bsms.ac.uk
Profiles: https://profiles.sussex.ac.uk/p262804-jo-middleton, https://orcid.org/0000-0001-5951-6608
Twitter: https://twitter.com/MedVetAcarology

Keywords: Infectious diseases & infestations < DERMATOLOGY; International health services < HEALTH SERVICES ADMINISTRATION & MANAGEMENT; Infection control < INFECTIOUS DISEASES; Mass Drug Administration; Neglected Diseases; QUALITATIVE RESEARCH

For peer review only - http://bmjopen.bmj.com/site/about/guidelines.xhtml

ABSTRACT

Objectives. Provide insights into the experiences and perspectives of healthcare staff who treated scabies or managed outbreaks in formal and informal refugee/migrant camps in Europe 2014–17.

Design. Retrospective qualitative study using semi-structured telephone interviews and framework analysis. Recruitment primarily through online networks of healthcare staff involved in medical care in refugee/migrant settings.

Setting. Formal and informal refugee/migrant camps in Europe 2014–17.

Participants. Twelve participants (four doctors, four nurses, three allied health workers, one medical student) who had worked in camps (six in informal camps, nine in formal ones) across 15 locations within seven European countries (Greece, Serbia, Macedonia, Turkey, France, the Netherlands, Belgium).

Results. Participants reported that in camps they had worked scabies diagnosis was primarily clinical (without dermatoscopy), and treatment and outbreak management varied highly. Seven stated scabicides were provided, five that only symptomatic management was offered. They described camps as difficult places to work, with poor living standards for residents. Key perceived barriers to scabies control were (i) lack of water, sanitation and hygiene, specifically: absent/limited showers (difficult to wash off topical scabicides); inability to wash clothes and bedding (may have increased transmission/re-infestation), (ii) social factors: language; stigma; treatment non-compliance; mobility (interfering with contact tracing and follow-up treatments), (iii) healthcare factors: scabicide shortages and diversity; lack of examination privacy; staff inexperience, (iv) organisational factors: overcrowding; ineffective inter-organisational coordination; lack of support and maltreatment by state authorities (e.g. not providing basic facilities; obstruction of self-care by camp residents and NGO aid).

Conclusions. We recommend development of accessible scabies guidelines for camps, use of consensus diagnostic criteria, and oral ivermectin mass treatments. In addition, as much of the work described was by small, volunteer staffed NGOs, we in the wider healthcare community should reflect how to better support such initiatives, and those they serve.

For peer review only - http://bmjopen.bmj.com/site/about/guidelines.xhtml

STRENGTHS AND LIMITATIONS OF THIS STUDY

- Recruitment through online healthcare staff networks enabled collection of the subjective perspectives of a participant population difficult to reach through formal means, given many had volunteered with NGOs but were not necessarily still anchored to them.
- Telephone interviews prevented observation of non-verbal cues, but did facilitate international involvement in a timely and resource-efficient manner.
- The relatively long and semi-structured nature of the interviews gave participants opportunity to articulate their perspectives, to an extent unconstrained by researcher presumptions over what may have been important to them about their experiences.
- Conducting data collection and thematic analysis simultaneously enabled assessment of data saturation during recruitment, but advertising and interviewing solely in English excluded experiences from non-English speaking individuals.
- The retrospective nature of the study may have introduced recall bias.

For peer review only

For peer review only - http://bmjopen.bmj.com/site/about/guidelines.xhtml

INTRODUCTION

Scabies is a stigmatised contagious skin condition caused by infestation with the mite *Sarcoptes scabiei*.[1] Transmission is mainly skin-to-skin, less commonly via fomites such as bedding.[2] Evidence from epidemiology and experimental trials indicates scabies prevalence is probably not influenced by levels of personal body washing.[3] Symptoms begin 3–6 weeks after first infestation, as early as one day after re-infestation.[4] Secondary bacterial infections are common,[5,6] with potential for serious long-term health impacts, including chronic rheumatic heart disease and chronic kidney disease.[7–9] Diagnosis is normally by clinical examination, sometimes using dermatoscopy.[4] Definitive diagnosis has been classified as requiring 'clinical features suggestive of scabies… plus a visible mite under dermatoscopy… or a skin scraping or biopsy with identified mites, mite eggs, or faeces'.[10] Simultaneous treatment for diagnosed individuals and close contacts is required.[11] This normally consists of topical scabicides applied to the entire body or oral ivermectin, in both cases often given twice (one week apart).[12] Environmental decontamination advice varies.[12] WHO designate scabies as a neglected tropical disease, with an estimated 455 million cases annually.[13] It's highest burden is in the tropics, but in lower-burdened regions (such as Europe) scabies remains common, emerging as a public health problem when institutional outbreaks occur.[10,13,14]

Over the last decade conflicts triggered large numbers of people to migrate to Europe.[15–17] By the end of 2016 European countries hosted 5.2 million refugees, 2.9 million in Turkey alone (mostly from Syria).[15] As a result, during 2014–17 many formal refugee/migrant camps and reception centres, and informal (often illegally occupied) camps came into being or expanded (figure 1). Healthcare services in camps were often limited to clinics operating from caravans or makeshift structures, largely staffed by volunteers; better resourced camps could still face shortages and overcrowding following unexpected influxes.[16, 18] Scabies has been one of the most frequently observed medical conditions in these settings.[18–22] In Germany, it was the third commonest outbreak type in asylum seeker shelters.[21] In France, Doctors of the World UK estimated in 2015 that up to 40% of those seeking their care in the Calais 'Jungle' camp had scabies (Cooper, online supplementary file). Outbreaks have been widely discussed in news and social media. Media analysis by Seebach *et al*.[23] described some of this coverage as stigmatising those affected by scabies, whilst positing migrants/refugees more generally as an invasive health threat to European countries. Multiple camps in France have been evicted with the public justification of responding to scabies outbreaks (examples of newspaper reports: Lichfield; Newton, online supplementary file). Despite all this, to our knowledge no study prior to ours has been published specifically on scabies outbreak diagnosis, treatment, and management in refugee/migrant camps in Europe during this period. Further, we are

For peer review only - http://bmjopen.bmj.com/site/about/guidelines.xhtml

unaware of any published study on healthcare staff perspectives on barriers and facilitators to treating and managing scabies in refugee camps globally.

Aims

This qualitative study aimed to provide insights into experiences and perspectives of healthcare staff who treated scabies or managed associated outbreaks in refugee/migrant camps in Europe 2014–17. Specifically, to describe: (i) methods used to diagnose, treat, and manage scabies; (ii) camp characteristics and perceived barriers and facilitators to effective scabies outbreak management.

METHODS

Study design

We carried out a retrospective qualitative study using semi-structured telephone interviews and framework analysis. Recruitment was through online networks of healthcare staff active in refugee/migrant settings. We report our work in line with the Standards for Reporting Qualitative Research guidelines[24] (checklist and researcher characteristics, online supplementary file).

Participant selection and recruitment

Healthcare staff from state and non-governmental organisations (NGOs) who had treated scabies in refugee/migrant camps in Europe, and/or managed associated outbreaks in the previous three years were eligible. We recruited by contacting:

(i) relevant healthcare staff known to us;

(ii) individuals publishing research on medical care in refugee/migrant settings;

(iii) through online networks of healthcare staff active in refugee/migrant settings, including the alumni network of the European Centre for Disease Control fellowship program and groups hosted on facebook.com. To target the latter a public page (https://web.archive.org/web/20180523163917/https://www.facebook.com/scabiesresearchproject/) was shared to ten relevant facebook.com hosted groups (online supplementary table 1) and messages sent to members whose posts indicated they fitted the inclusion criteria.

Data collection, processing, and analysis

Audio-recorded, semi-structured telephone interviews were conducted from a private room in the Department of Primary Care and Public Health at Brighton and Sussex Medical School by first author NR November 2017 to February 2018. Participants gave data on personal characteristics and experience, and interviews followed a topic guide based on published literature and guidance from experts in scabies (epidemiology, JAC; dermatology, SLW; medical acarology, JM). They were scheduled for c.40mins, but so as to provide sufficient information power[25] participants were told

For peer review only - http://bmjopen.bmj.com/site/about/guidelines.xhtml

131 they could speak longer as necessary, and to not feel precluded by the topic guide from relating any
132 experiences and perspectives they thought relevant to the study aims. NR conducted data collection
133 and thematic analysis simultaneously to determine when data saturation was reached,[26,27] which
134 was prospectively defined as being no new themes emerging in three consecutive interviews. Once
135 data saturation was observed no new interviews were conducted as the team could 'be reasonably
136 assured that further data collection would yield similar results and serve to confirm emerging themes
137 and conclusions.'[28] Transcription was undertaken by professional service (9 of 12 interviews), or
138 by NR (3). NR coded text in NVivo 11 (QSR International, Melbourne), generated an initial
139 framework matrix and exported it to Microsoft Excel, in which qualitative framework analysis was
140 completed (as outlined by Gale *et al.*[29]). Groups of meaningful concepts were sought in the data
141 and arranged hierarchically into main themes and subthemes. Each transcript was studied again twice
142 for more evidence of the concepts. Subsequently author JM reviewed the reported themes and
143 subthemes and read the transcripts, finding no new relevant themes or subthemes than those grouped
144 by NR.

145 Patient and public involvement

146 Patients and the public were not involved in research design. Members of healthcare staff networks
147 aided recruitment. CS is an aid worker with scabies outbreak experience in the study setting and co-
148 authored the paper (they were not an interviewee).

149 RESULTS

150 Participants

151 Recruitment halted after 12 interviews as data saturation was assessed to have been reached (no new
152 themes emerged in the last three interviews). Interviews lasted 34–71min (mean 47). The twelve
153 interviewees consisted of four doctors, four nurses, three allied health workers (AHWs: an ECG
154 technician, podiatrist, and a first aider), and one medical student. Ten were NGO volunteers, one an
155 independent volunteer, one was employed by a public health service. Six had worked in informal
156 camps, nine in formal ones, across camps at 15 locations within seven European countries: Greece,
157 France, the Netherlands, Serbia, Belgium, Turkey, Macedonia. Geographic distribution of the camps
158 (figure 2) reflected eastern entry migration routes into Europe, and clustering at the English Channel
159 by those seeking to enter the UK. Experience in camps ranged from 4 d to 2.5 y (median 84 d, IQR
160 233–251 d) (participant characteristics, online supplementary table 3).

161 Diagnosis, treatment, and outbreak management

162 Scabies diagnosis, treatment and outbreak management are summarised in table 1. Eleven
163 interviewees (92%) diagnosed scabies based on history and clinical findings. Two AHWs and the

For peer review only - http://bmjopen.bmj.com/site/about/guidelines.xhtml

medical student confirmed diagnoses with a doctor. One participant occasionally used skin-scrapings in a Greek formal camp and during an outbreak in Serbia. In one camp diagnosis was routinely given by a dermatologist and confirmed by microscopic examination. No one reported dermatoscopy. Three organisations attempted to follow Médecins Sans Frontiers' (MSF) guidelines for ordinary scabies treatment[30] (online supplementary file), one combined this with British National Formulary advice.[31] Another had their own scabies protocol.

Only seven participants (58%) reported scabicides were provided in camps they worked in whilst they were there. The remaining five (42%) stated only symptomatic management was offered (detailed in table 1) because it was felt proper treatment would be futile due to high re-infestation risks (four participants), or due to prescribing restrictions (one participant). A third of participants said treatments varied, depending on availability. When NGOs offered scabicides, they were generally given to anyone complaining of itch, due to a low threshold of suspicion (5 of 7). However, one NGO only treated individuals with a dermatologist-confirmed diagnosis, another only treated 'serious cases'.

Eleven participants (92%) believed scabies was difficult to manage, whereas a GP following a formal camp's scabies protocol considered it "just a nuisance" (P7). Interviewees described high variation in outbreak management (table 1), particularly in timings used for permethrin application. These ranged from singular treatment to second applications up to a week later. Three participants (25%) could not recall timings or if close contacts were treated. In four interviews, participants claimed to treat all close contacts regardless of symptoms. Only three participants (25%) reported use of oral ivermectin for scabies, which was limited due to lack of availability. Five interviewees (42%) reported environmental decontamination was considered too challenging to conduct. Cleaning/replacing clothing and bedding was the most common form of decontamination. Individuals were given replacement clothing/bedding and old items washed (2 of 7), disposed of (3 of 7), or put into plastic bags for 72 h (2 of 7). One formal camp in Greece burned blankets. One organisation built an inventory of rental clothes for individuals while theirs were in bags. Generally, decontamination of furniture and shelters was not attempted (only mentioned in two interviews). Where it was, it involved disinfectant or covering furniture and insides of shelters in plastic sheeting for 72 h.

For peer review only - http://bmjopen.bmj.com/site/about/guidelines.xhtml

Table 1. Diagnosis, treatment, and outbreak management of scabies (n=12 participants)

**Diagnosis**		
**Clinical features observed in patients**	Itching, mainly at night	12
	Rash	7
	Burrow lines	6
	Infected lesions	2
**Parts of the body examined**	Hands	8
	Arms	7
	Torso	6
	Genitals	4
	Legs	3
	Feet	3
	Armpits	2
**Equipment used during examination**	Gloves	7
	Nothing	2
	Lights	2
	Microscope	2
	Dermatoscope	0
**Duration of examination**	<5 minutes	8
	5–10 minutes	2
	>10 minutes	1
**Treatment**		
**Treatment guidelines**	None	8
	MSF clinical guidance	3
	British National Formulary	1
	NGO's own protocol	1
**Management of individual**	Symptomatic management only	5
	Antihistamines	3
	Topical steroid	1
	Moisturizer	1
	Antibiotics for infections	1
	Varying treatment depending on availability	4
	Unknown topical treatment	3
	1st line: permethrin 5% cream. 2nd line: benzyl benzoate. Symptomatic treatment: antihistamines.	2
	1st line: ivermectin PO. 2nd line (or if ≤15kg): permethrin 5% cream	2
	Permethrin 5% cream only	1
**Who was given acaricide treatment**	No one	5
	Anyone with itching, plus close contacts	4
	Anyone with itching	1
	Only "serious cases"	1
	Cases confirmed by dermatologist, plus close contacts	1
**Outbreak management**		
**Timing of mass treatments**	No treatment	4
	Unknown	3
	Cases treated twice, one week apart (permethrin)	1
	Cases & symptomatic contacts treated x2 a few days apart	1
	(permethrin), asymptomatic contacts x1	1
	Cases treated x1, 3 days apart (permethrin)	1
	Cases treated x2, 24 hours apart (permethrin and benzyl benzoate)	1
	Case treated x1 (permethrin)	1
	Cases treated x1 (ivermectin)	
**Treatment of close contacts**	No treatment available to anyone	4
	All close contacts	4
	Unknown	3
	Symptomatic contacts	1
**Environmental decontamination**	Clothing	7
	Bedding	7
	None	5
	Furniture	1
	Shelters	2

For peer review only

For peer review only - http://bmjopen.bmj.com/site/about/guidelines.xhtml

Camp characteristics and perceived barriers and facilitators to effective scabies outbreak management

Camp Characteristics

All participants described camps as difficult places to work; legal status seemed unconnected to environmental quality. Participants described poor living standards (quotes, online supplementary file, p.6–8). For example, P8 described a formal camp (Greece):

> "It's horrible. There's only one water source for over two thousand people, there's not enough clothes, people are sleeping outside, there's not enough tents, the hygiene is terrible. I was there in the summer so it was hot, there's not enough shading and people couldn't find a place outside the sun. It's more horrible now, as its winter, so it's freezing, and there's no heating. To be honest it's worse than the refugee camps I've seen in Africa."

P9, who had worked in formal and informal camps (France and Greece) spoke similarly:

> "They are a human rights violation. I don't think people should be living in them, especially in a first world European country this should not be happening. People are living in cold tents without heating and electricity, there's rats, there's scabies, there's a lack of toileting facilities, a lack of hygiene, lack of food."

Ten participants (83%) believed shelter was inadequate or non-existent, two (17%) very basic. Twelve (100%) described lack of suitable Water, Sanitation, and Hygiene (WaSH) facilities. Five (42%) described safety worries regarding: fights between residents; police mistreatment; instability of camp structures.

Barriers and facilitators to effective scabies outbreak management

Themes and subthemes are illustrated by example quotes in tables 2 (barriers) and 3 (facilitators), with 16 pages of themed quotes in online supplementary file. Each interviewee described many barriers to effective scabies management; four key themes arose.

Lack of WaSH facilities: Lack of access to washing facilities for fabrics to prevent re-infection was identified by 10 interviewees (83%). Three interviewees (25%) believed sharing belongings was promoting re-infestation, amplified by inability to wash them between users. Six interviewees (50%) described difficulties associated with camp residents inability to wash themselves, due to non-existent or insufficient showers. This was especially relevant regarding topical scabicides, which should be washed off.[4]

For peer review only - http://bmjopen.bmj.com/site/about/guidelines.xhtml

Table 2. Barriers to providing effective scabies management.
Further themed participant quotes in online supplementary file, p.8–16.

**Key Themes** *Subthemes*	**Comments from participants**
**Lack of WaSH Facilities**	
Inability to wash belongings	"Part of the treatment is also to wash the bedding, and all clothing in hot water, dry it in a drier... those facilities weren't readily available for anybody at the camp... even if we'd had a sort of endless supply of Permethrin or Ivermectin, I think it still would have been problematic with this problem of re-infection." (P6)
Lack of showering facilities	"There is really no shower and there are some areas that are a kind of shower area, but it's not really a shower and its cold water." (P5)<br><br>"Treating scabies was problematic because… the lotion, you're supposed to keep it on for 12 hours and then wash it off… and access to showers was not always that easy." (P6)
Sharing of belongings	"Charities came in to give clothes, but the clothes weren't ever washed and when someone went across the border... their stuff was left, and whoever was in the tent just used that." (P1)
**Social**	
Language barriers	"They don't understand why they have to use the cream and what's going on. So, they feel reluctant to comply, and they're like no, it doesn't work. If you don't have proper translators to translate anything to you that also could be a big barrier." (P2)
Stigma	"Didn't want to tell the other people they had scabies... there is some sort of stigma of course if you have some sort of rash that you are not clean or something." (P8)
Non-compliance to treatment	"I would say to put the stuff all over your body, head down to the toes... to do it all in one night and keep it on for so many hours and then they walk away and then only put a bit of cream here, and a bit of cream there, for a week and then they come back and it hasn't got better." (P7)
Transient population	"You have to trace who might be the contact... in the refugee setting you have to also to treat patients that might be from the same family, and they just move around. You can't really tell who has slept here, who has not slept here, who might possibly get it." (P2)
**Healthcare**	
Availability of medication	"We don't have the treatment for it, because we don't have the money to buy in the treatment." (P4)<br><br>"The medication available is the least convenient medication you could use. If you could use a pill for everyone that would be so much easier." (P10)
Lack of privacy for medical examinations	"Everything sees what we are doing, so if someone has to show something we don't have a closed examination room... you can't really examine them... you can't really touch and see in your own eyes... you have to rely on the photos because they're too shy to show it." (P2)
Inexperienced staff	"European doctors did not have a lot of experience with scabies, so I think a lot of them didn't realize what they were seeing." (P10)
**Organisational**	
Crowded living conditions	"People were living in fairly cramped conditions which obviously made transmission a high possibility." (P6)
Poor coordination between organisations	"Lack of coordination and appropriate communication... I don't think it's the resources; the resources are there they're just not put together in the right way." (P10)<br><br>"Logistical problems, sufficient manpower, sufficient washing machines, sufficient bedding, who cares for this?" (P11)
Poor treatment by authorities	"refugees have their bedding and their coats and shoes confiscated by police at night." (P3)<br><br>"People were hiding from the authorities... the settlement or their place where they would sleep would not be there because tents were being like routinely slashed and belongings were being destroyed by the police and authorities." (P12)

Social barriers*:* Eight interviewees (67%) felt language differences obstructed understanding individuals' problems and explaining treatment and decontamination. Four (33%) felt stigma associated with scabies adversely affected management, as it reduced presentation to services and individuals were less likely to inform close contacts who required treatment. Treatment non-compliance was considered a barrier in four interviews (33%), caused by complicated management

For peer review only - http://bmjopen.bmj.com/site/about/guidelines.xhtml

regimes, and residents' competing priorities. Population mobility concerned six interviewees (50%), who felt it affected contact tracing, follow-up treatment, and disrupted elimination efforts.

Organisational barriers: Six participants (50%) perceived overcrowding as a major barrier to effective management. Large dormitory-style rooms were said to be the hardest in which to determine close contacts, as were bigger units in which blanket partitions were used between families. Five interviewees (42%) described poor coordination between organisations (both NGOs and governmental organisations) as a barrier. This primarily involved organisations providing different resources not communicating about appropriate timings (e.g., lack of connection between treatments, clothing replacement, access to showers). Two participants described disrespectful care and stigmatisation by healthcare workers. Ten participants (83%) described how lack of support and mistreatment by state authorities acted as a major barrier, most of which was described in informal camp settings (7 of 10). Two interviewees mentioned residents facing police brutality, including having tents slashed, and clothing, shoes, and bedding confiscated. This encouraged sharing (with potential implications for transmission), and by adding further challenges to everyday life it reduced volunteer and patient capacity to focus on treatment and decontamination. One participant reported authorities "sometimes they cut… water, they try not to get us to be able to give out clothes, they raided our distribution place." (P2) Their explanation was: "What the government is trying to do is, they're trying not to get the refugees too comfortable."

Healthcare barriers: Eight interviewees (67%) considered scabicide availability a major barrier. Cost, prescribing restrictions, and variations in pharmacy stocking contributed; for most NGOs availability relied on donations. Inconsistency complicated treatment and fostered mistrust. Three interviewees (25%) felt lack of privacy impacted care. Some residents attempted to circumvent this by providing photos on phones, but this was not considered as useful as examinations. Five interviewees (42%) felt healthcare workers accustomed to European work were unfamiliar with scabies, which was exacerbated by volunteer-turnover.

Facilitators: Two participants (17%) said they did not experience factors aiding scabies treatment and management, the remaining described five, when available: WaSH facilities; language facilitators; education; treatment incentives; coordination of organisations (table 3). Three (25%) felt access to showers and washing machines was vital, as clothing and bedding could be reused without concern of reinfection, and topical treatment guidelines adhered to. Nine interviewees (75%) described the importance of translators in overcoming language barriers. Only three NGOs used official translators, the rest were aided by residents. One participant described using recorded voice messages and information sheets in different languages. Four (33%) believed resident education aided outbreak management, by alerting them to others who needed treatment and by providing education on correct treatment application. This was also thought to have reduced stigma within camps. Four (33%) stated

For peer review only - http://bmjopen.bmj.com/site/about/guidelines.xhtml

incentives (e.g., new clothing or just underwear) aided treatment compliance. Eight interviewees (67%) reported positive impacts from inter-organisational communication, including information and resource sharing, and enabling referrals. The GP who described scabies as "just a nuisance" (P7), attributed that to having a protocol followed by all organisations. In particular, the "ability to pass on a systematic and well-rehearsed message" (P7) was thought to have improved adherence.

Table 3. Facilitators to providing effective scabies management.

Further themed participant quotes in online supplementary file, p.18–21.

**Key Themes** *Subthemes*	**Comments from participants**
**WaSH Facilities**	"People have access to showers... clothes had to be washed in the washing machines… everything else was taken out and burnt. That was the protocol... we didn't have outbreaks." (P7)
**Social** *Language facilitators*	"We had our own translator who spoke several languages, and there were people who lived in the camp, working or volunteering as translators." (P1)
Education	"If you educate the patient very well, make them understand very well what's going on, and what needs to be done... They understand, and they start to even manage to recognise their friends." (P2)
**Treatment incentive**	"When they found out we would give them another set of clothes and we would buy underwear for them, some more people were interested as they had an incentive to take part." (P8)
**Coordination of organisations**	"We give them a cream 24 hours before and then asked them to get showers, and then we put the creams again. So we partner with shower teams, and also, we partner with people from the distribution team so we could get them new fresh clothes." (P2)

DISCUSSION

Principal findings

Participants reported in the camps they had worked scabies diagnosis was primarily clinical (without dermatoscopy), and treatment and outbreak management varied highly. Nearly half said scabicides were unavailable; only symptomatic management was offered. They described camps as difficult places to work, with poor living standards for residents. Key perceived barriers to control were (i) lack of WaSH facilities, specifically: absent/limited showers (difficult to wash off topical scabicides); inability to wash clothes and bedding (may have increased transmission/re-infestation), (ii) social factors: language; stigma; treatment non-compliance; mobility (interfering with contact tracing and follow-up treatments), (iii) healthcare factors: scabicide shortages and diversity; lack of examination privacy; staff inexperience, (iv) organisational factors: overcrowding; ineffective inter-organisational coordination; lack of support and maltreatment by state authorities (e.g. not providing basic facilities; obstruction of self-care by camp residents and NGO aid).

Strengths and weaknesses

This qualitative study succeeded in giving voice to the subjective perspectives and experiences of healthcare staff involved in this previously unstudied topic. However, recruitment methods may have prevented fully representative participant selection. Firstly, because recruitment and interviews were

For peer review only - http://bmjopen.bmj.com/site/about/guidelines.xhtml

conducted in English, excluding experiences from non-English speaking individuals. Secondly, as we assessed data saturation had been reached and halted recruitment, there was a fairly small number of participants. Notably though, our sample size does correspond with what Guest et al.[26] determined was usually sufficient to reach data saturation in qualitative studies focused on narrow topics. One study strength was that the relatively long semi-structured interviews gave participants opportunity to articulate their perspectives relatively unconstrained by researcher presumptions over what may have been important to them about their experiences. Telephone interviews prevented observation of non-verbal cues, but did enable international recruitment. The retrospective nature of the study may have introduced recall bias. Inference and public health recommendations based on this research must be pragmatic with limitations acknowledged.

Louka et al.[22] report scabies epidemiology in refugee/migrant populations in Greece, while more general quantitative studies from across Europe touch on scabies in these populations (for example, see [19-21]). However, to our knowledge our study is the only qualitative work published on healthcare staff perspectives on barriers and facilitators to treating and managing scabies within refugee/migrant camps, either in Europe or globally. This underlines the major strength of our study in providing a platform for these underrepresented healthcare voices, yet given this it is difficult to compare our findings to others. Wollina et al.[32] do usefully recount their related experience in a German hospital which received dermatological referrals (scabies being the most common) from a nearby formal refugee camp. Even in this more resourced setting language barriers were an issue, as they were for some of our participants. An environmental health assessment of a French informal camp with a high scabies incidence detailed a very similar environment to those described by many of our participants: 'Shelters were vastly inadequate and directly exacerbating the ill-health and psychological distress suffered by some residents of the camp... Residents at all sites reported difficulties with washing themselves, their clothes and their bedding.'[18]

Implications for clinicians and policymakers

Diagnosis and treatment

It is unsurprising no participants reported dermatoscopy use given equipment expense, paucity of trained individuals, and its reduced sensitivity when outbreak size limits examination time.[33] However, subsequently published consensus criteria for scabies diagnosis[34] provides a standard for clinical diagnosis, though it requires validating in these settings. Some participants suggested screening on entry to camps to reduce ingress of new infestations. This was also suggested by Wollina et al.[32], has been carried out in reception centres[35], and may be feasible in camps. However, in informal camps screening may not be consented to by those seeking entry and NGOs may have insufficient resources to adequately screen, potentially leading to false positives/negatives. The

For peer review only - http://bmjopen.bmj.com/site/about/guidelines.xhtml

asymptomatic incubation period would also mean some potential outbreak index cases would still be missed.

Participants reported problems using topical scabicides. Firstly, topical treatment involves full body coverage with creams, left on for at least half a day. Privacy was limited and individuals living in close proximity may have wished to avoid being seen treating themselves for a stigmatised condition, thus discouraging appropriate application. Secondly, topical scabicides need be washed off to reduce potential irritancy[4] but facilities were usually only minimally available (if at all). Oral ivermectin Mass Drug Administrations (MDAs) are easier to administer, and were conducted successfully in Dutch and German reception centres during the period.[35, 36] However, ivermectin is expensive in Europe and unlicensed for scabies in most European countries[12] despite a good safety profile,[37,38] and inclusion in WHO's essential medicines list.[39] The potential health impacts of chronic conditions secondary to scabies[7–9] supports the value of timely MDAs in these settings. Participants reported widespread use of camp residents as informal translators, but this raises confidentiality concerns, and ad-hoc interpreters often misinterpret/omit important medical information.[40]

Outbreak management and camp environments

Discrepancies in management reflect lack of widely accepted standard outbreak guidelines. Guidance (for example,[30, 31]) is often for individual households and does not make adaptions based on the logistical difficulties of camp environments. Institutional scabies outbreaks guidelines exist, yet none are based on a systematic review of the published evidence.[12] This is also the case for the 'European Guideline'.[41] More broadly there is a lack of research regarding the most effective interventions for preventing scabies transmission to close contacts,[42] particularly in refugee/migrant settings. Organisations often favoured attempting to decontaminate clothing and bedding rather than shelters, yet living mites have been found in dust samples on floors and furniture.[43] Variance in reported decontamination reflects a sparse historic evidence-base.[12] However, recent experimental work[44] could support uniform action. Where washing/drying machines are available (few camps in this study) ≥10min at ≥50°C will kill all *S.scabiei* mites and eggs.[44] Lacking such resources, some participants reported isolating potential fomites with plastic bags/sheeting for 72 h. Due to mite desiccation this may have been effective in temperate-dry settings, but if bags were left outdoors in the cold and damp mite survival can be longer. At the outer limit, in warm-humid conditions elimination can take >8 d.[44] Existing guidance should be updated. Even though improving WaSH facilities is unlikely to reduce skin-to-skin transmission,[3] it nevertheless would support treatment and control where transmission via fomites may be a risk, when topical scabicides are used, and to minimise secondary infections.[14] It could also be expected to reduce transmission of other diseases.

For peer review only - http://bmjopen.bmj.com/site/about/guidelines.xhtml

Participants described crowded and substandard living conditions that likely provided an ideal environment for scabies transmission, whilst obstructing treatment and outbreak management. Some believed lack of political will was preventing provision of suitable living conditions and access to healthcare, as governments feared they would encourage people to stay too long. Going further, participants reported actual obstruction by authorities of resident self-care and NGO aid efforts, with implications for transmission and control of scabies. Without support and coordination of all those responsible for care of refugees/migrants, successful scabies management will remain challenging, and the resource poor and overcrowded aspects of camp environments which enable outbreaks will remain unchanged. Indeed, in late 2020 a similar situation to those described in 2014–2017 was observed by co-author CS in a formal camp on Lesvos (Greece). The NGO CADUS, in which CS works, had been deployed within a WHO initiative to provide medical care. Scabies was widespread among the camp population of 7500, but the eradication program had to begin with a capacity of ten treatments per day. A small volunteer-staffed NGO provided treatment, including provision of (camp-external) showers, clothes and blankets exchange. The overcrowded camp lacked adequate facilities to wash clothes, and mostly only symptomatic treatment could be offered, which CADUS medical staff viewed as highly unsatisfactory.

Future Recommendations

Our study demonstrates the importance of having clear protocols for scabies diagnosis, treatment, and management in refugee/migrant camps. Systematic evidence evaluation must be combined with input from individuals and organisations on-the-ground to provide high-quality feasible guidelines that explicitly consider camp settings, formal and informal. Given most participants were not doctors, guidelines need to be understandable to everyone involved. Though topicals should be provided for children and pregnant women, difficulties associated with them in camps could be avoided for most adult patients through wider licensing and availability of oral ivermectin. The 'WHO Informal Consultation on a Framework for Scabies Control' recommended populations with ≥10% prevalence should receive ivermectin MDAs;[45, 46] we suggest this should include refugee/migrant camps. Strategies for lower levels of endemicity in camps should also be investigated. More research and advocacy for those living in refugee/migrant camps in Europe could help improve management of common health problems. This is particularly relevant for managing scabies in camps, where substandard conditions, inadequate resources, and lack of guidance can lead to poor quality of care and ineffective treatment. As our participants indicate, much of this work is shouldered by small, volunteer staffed NGOs operating with minimal resources in highly challenging (often illegally occupied) settings. We in the wider healthcare community should reflect on how we can better support these initiatives, and those they serve.

For peer review only - http://bmjopen.bmj.com/site/about/guidelines.xhtml

STATEMENTS

Online supplementary file: This web only file has been produced by the BMJ Publishing Group from an electronic file supplied by the authors and has not been edited for content.

Contributors: This study was carried out as part of the work of the Scabies Research Team based at Brighton and Sussex Medical School, London School of Hygiene and Tropical Medicine, and University of Southampton. Authorship order is alphabetical by surname, except the first (primary researcher) and last (lead supervisor). For clarity we detail contributions using the CRediT Contributor Taxonomy (https://credit.niso.org/), and provide employment and disciplinary descriptions. Conceptualisation: JM, JAC. Formal analysis: NR, JM. Investigation: NR, JM. Methodology: JM. Project administration: NR, JM. Supervision: SL, JM. Writing – original draft: NR, JM. Visualization: NR, JM. Writing – review & editing: NR, JAC, MGH, SL, CS, SLW, JM. NR is a global emergency medicine clinical fellow; JAC a public health physician and epidemiologist; MGH a senior research fellow [global health]; SL a research co-ordinator; CS is an aid worker; SLW a consultant dermatologist, and associate professor [infectious and tropical diseases]; JM a research fellow [public health; neglected tropical skin diseases].

Ethics: This study involves human participants and was approved by Brighton and Sussex Medical School Research Governance and Ethics Committee approved the research (ER/BSMS3398/1).

Acknowledgments: We thank the interviewees for their time and openness with our study, and the administrators and members of the healthcare staff networks who advertised our study on-line. We are also grateful to our colleagues at Brighton and Sussex Medical School, Elizabeth Ford (for input into study design), Jessica Stockdale (for producing figure 2), and Maya Khan (for setting up our Facebook recruitment pages).

Data availability: Online supplementary file includes 16 pages of themed quotes from interviews. Other data are available on reasonable request from the corresponding author.

Funding: This research was financially supported by Brighton and Sussex Medical School from internal funds and did not receive any specific grant from funding agencies in the public, commercial, or not-for-profit sectors.

Competing interests: None declared.

REFERENCES

1. Trettin B, Lassen JA, Andersen F, et al. The journey of having scabies– a qualitative study. J Nurs Educ 2019;9:2. doi: 10.5430/jnep.v9n2p1

For peer review only

For peer review only - http://bmjopen.bmj.com/site/about/guidelines.xhtml

2. Mellanby K. Scabies in 1976. J R Soc Promo Health 1977;97:32–36. doi: 10.1177/146642407709700112
3. Middleton J, Cassell JA, Jones CI, et al. Scabies control: the forgotten role of personal hygiene- Authors reply. Lancet Infect Dis 2018;18:1068-1069. doi: 10.1016/S1473-3099(18)30558-9
4. Griffiths C, et al. Rook's Textbook of Dermatology. New York: Wiley-Blackwell 2016:1111–1119.
5. Swe PM, Reynolds SL, Fischer K. Parasitic scabies mites and associated bacteria joining forces against host complement defence. Parasite Immunol 2014;36:585–593. doi: 10.1111/pim.12133
6. Romani L, Steer AC, Whitfeld MJ, et al. Prevalence of scabies and impetigo worldwide: a systematic review. Lancet Infect Dis 2015;15:960–967. doi: 10.1016/S1473-3099(15)00132-2
7. Chung SD, Wang KH, Huang CC, et al. Scabies increased the risk of chronic kidney disease: a 5-year follow-up study. J Eur Acad Dermatol 2014;28:286–292. doi: 10.1111/jdv.12099
8. Thornley S, Marshall R, Jarrett P, et al. Scabies is strongly associated with acute rheumatic fever in a cohort study of Auckland children. J Paediatr Child H 2018;54:625–632. doi: 10.1111/jpc.13851
9. Thornley S, King R, Marshall R, et al. How strong is the relationship between scabies and acute rheumatic fever? An analysis of neighbourhood factors. J Paediatr Child H 2020;56:600–606. doi: 10.1111/jpc.14697
10. Cassell JA, Middleton J, Nalabanda A, et al. Scabies outbreaks in ten care homes for the elderly: a prospective study of clinical features, epidemiology, and treatment outcomes. Lancet Infect Dis 2018;18:894–902. doi: 10.1016/S1473-3099(18)30347-5
11. NICE. Clinical Knowledge Summaries: Scabies [online]. 2017. https://cks.nice.org.uk/scabies#!scenario (accessed 10 Nov 2017).
12. White LCJ, Lanza S, Middleton J, et al. The management of scabies outbreaks in residential care facilities for the elderly in England: a review of current health protection guidelines. Epidemiol Infect 2016;144;3121–3130. doi: 10.1017/S0950268816001746
13. Engelman D, Cantey PT, Marks M, et al. The public health control of scabies: priorities for research and action. Lancet Infect Dis 2019;394:81–92. doi: 10.1016/S0140-6736(19)31136-5
14. Middleton J, Cassell JA, Walker SL. Scabies management in institutions. In: Chosidow and Fisher eds. Scabies. Vienna: Springer Nature In Press [expected publication summer 2023].
15. UNHCR. Global Trends 2016 [online]. 2017. http://www.unhcr.org/5943e8a34.pdf (accessed 14 July 2018).
16. WHO Europe. Greece: assessing health-system capacity to manage large influxes of migrants [online]. http://www.euro.who.int/__data/assets/pdf_file/0007/300400/Greece-Assessment-Report-en.pdf?ua=1 (accessed 10 Nov 2017).
17. Catchpole M, Coulombier D. Refugee crisis demands European Union-wide surveillance! Eurosurveillance 2015;20. doi: 10.2807/1560-7917.ES.2015.20.45.30063
18. Dhesi S, Isakjee A, Davies T. An Environmental Health Assessment of the New Migrant Camp in Calais [online]. 2015. https://www.birmingham.ac.uk/Documents/college-les/gees/research/calais-report-oct-2015.pdf (accessed 10 Nov 2017).
19. Kortas AZ, Polenz J, von Hayek J, et al. Screening for infectious diseases among asylum seekers newly arrived in Germany in 2015: A systematic single-centre analysis. Public Health 2017;153:1. doi: 10.1016/j.puhe.2017.07.011

For peer review only - http://bmjopen.bmj.com/site/about/guidelines.xhtml

20. Alberer M, Malinowski S, Sanftenberg L, et al. Notifiable infectious diseases in refugees and asylum seekers: Experience from a major reception center in Munich, Germany. Infection 2018:1–9. doi: 10.1007/s15010-018-1134-4

21. Kühne A, Gilsdorf A. [Infectious disease outbreaks in centralized homes for asylum seekers in Germany from 2004–2014.] Ausbrüche von Infektionskrankheiten in Gemeinschaftsunterkünften für Asylsuchende 2004–2014 in Deutschland] Bundesgesundeheitsbla 2016;59:570–577. doi: 10.1007/s00103-016-2332-9

22. Louka C, Logothetis E, Engelman D, et al. Scabies epidemiology in health care centers for refugees and asylum seekers in Greece. PLoS Negl Trop Dis 2022;16: e0010153. doi: 10.1371/journal.pntd.0010153

23. Seebach S, Baleriola E, Valasquez MM, et al. Fear of and anger against the other - the strange, the sick and the imaginary struggle for survival. Digithum: Las humanidades en la era digital 2016;18:13–24. doi: 10.7238/d.v0i18.2811

24. O'Brien BC, Harris IB, Beckman TJ, et al. Standards for reporting qualitative research: a synthesis of recommendations. Acad Med 2014;89:1245-1251. doi: 10.1097/ACM.0000000000000388

25. Malter K, Siersm VD, Guassora AD. Sample Size in Qualitative Interview Studies: Guided by Information Power. Qual Health Res 2016;26:1753–1760. doi: 10.1177/1049732315617444

26. Guest G, Bunce A, Johnson L. How Many Interviews Are Enough?: An Experiment with Data Saturation and Variability. Field Method 2006;18:59–82. doi: https://doi.org/10.1177/1525822X05279903

27. Fusch PI, Ness LR. Are We There Yet? Data Saturation in Qualitative Research. Qual R 2015;20:1408–1416. doi: 10.46743/2160-3715/2015.2281

28. Faulkner SL, Trotter SP. Data Saturation. In: Matthes J, Davis CS, Robert F, et al., eds. International Encyclopaedia of Communication Research Methods. New Jersey: Wiley 2017. doi: 10.1002/9781118901731.iecrm0060

29. Gale NK, Heath G, Cameron E, et al. Using the framework method for the analysis of qualitative data in multi-disciplinary health research. BMC Med. Res. Methodol 2014;13:117. doi: 10.1186/1471-2288-13-117.

30. Médecins Sans Frontiers. Clinical Guidelines: Diagnosis and treatment manual [online]. 2016. http://refbooks.msf.org/msf_docs/en/clinical_guide/cg_en.pdf (accessed 1 April 18).

31. Joint Formulary Committee. British National Formulary [edition number uncollected]. London: BMJ Group and Pharmaceutical Press [year of publication uncollected].

32. Wollina U, Gaber B, Mansour R, et al. Dermatologic challenges of health care for displaced people. Our Dermatol Online 2016;7:136–138. http://www.odermatol.com/odermatology/20162/2.Dermatologic-WollinaU.pdf

33. Walker SL, Middleton J, Cassell JA. Scabies outbreaks in care homes for the elderly – Authors' reply. Lancet Infect Dis 2019;19:26–27. doi: 10.1016/S1473-3099(18)30668-6

34. Fuller LC, Steer AC, et al. Consensus criteria for the diagnosis of scabies: A Delphi study of international experts. PLoS Neglect Trop D 2018;12:e0006549. doi: 10.1371/journal.pntd.0006549 .

35. Beeres DT, Ravensbergen SJ, Heidema A, et al. Efficacy of ivermectin mass-drug administration to control scabies in asylum seekers in the Netherlands: A retrospective cohort study between January 2014 – March 2016. PLoS Neglect Trop D 2018;12:e0006401. doi: 10.1371/journal.pntd.0006401.

For peer review only

For peer review only - http://bmjopen.bmj.com/site/about/guidelines.xhtml

36. Solbach P, Sdlacek L, Schmidt RE, et al. [Mass therapy with ivermectin for scabies outbreak during the refugee crisis in Germany in 2015] Systemische antiinfektive Therapie eines Skabiesausbruchs mit Ivermectin in einem Flüchtlingslager. Anästh Intensivmed 2017;58:534–541. doi: 10.19224/ai2017.534

37. Marks M, Taotao-Wini B, Satorara L, et al. Long-Term Control of Scabies Fifteen Years after an Intensive Treatment Programme. PLoS Neglect Trop D 2015;9:e0004246. doi: 10.1371/journal.pntd.0004246

38. Kinyanjui T, Middleton J, Guttel S, et al. Scabies in residential care homes: Modelling, inference and interventions for well-connected population sub-units. PLoS Comput Biol 2018;14:e1006046. doi: 10.1371/journal.pcbi.1006046.

39. Middleton J, Walker SL, House T, et al. Ivermectin for the control of scabies outbreaks in the UK. Lancet 2019;10214:2068–2069. doi: 10.1016/S0140-6736(19)32619-4

40. Flores G. The impact of medical interpreter services on the quality of health care: a systematic review. Med Care Res Rev 2005;62:255–299. doi: 10.1177/1077558705275416

41. Salavastru CM, Chosidow O, Boffa MJ, et al. European guideline for the management of scabies. J Eur Acad Dermatol 2017;31:1248–1253. doi: 10.1111/jdv.14351

42. FitzGerald D, Grainger RJ, Reid A. Interventions for preventing the spread of infestation in close contacts of people with scabies. Cochrane DB Syst Rev 2014:CD009943. doi: 10.1002/14651858.CD009943.pub2

43. Arlian LG, Estes SA, Vyszenski-Moher DL. Prevalence of Sarcoptes scabiei in the homes and nursing homes of scabietic patients. J Am Acad Dermatol 1988;19:806–811. doi: 10.1016/s0190-9622(88)70237-6

44. Bernigaud C, Fernando DD, Lu H, et al. How to eliminate scabies parasites from fomites: A high-throughput ex vivo experimental study. J Am Acad Dermatol 2020;83:241–245. doi: 10.1016/j.jaad.2019.11.069

45. WHO. WHO informal consultation on a framework for scabies control, meeting report [online]. 2019. https://www.who.int/publications/i/item/9789240008069 (accessed 16 April 2021).

46. Engelman D, Marks M, Steer AC, et al. A framework for scabies control. PLoS Neglect Trop D 2021;15: e0009661. doi: 10.1371/journal.pntd.0009661

FIGURES

Figure 1. Informal and formal refugee/migrant camps.
Left: informal camp in Calais, France, 2015 (VOA News - Nicolas Pinault (2015), public domain, original: https://web.archive.org/web/20210406154625/https://commons.wikimedia.org/wiki/File:Calais2015a.jpg). Right: formal camp Thessaloniki, Greece, 2016 (Konstantinos Stampoulis (2016), used under CC BY-SA 4.0 which also applies to this figure, original: https://web.archive.org/web/20210406155208/https://commons.wikimedia.org/wiki/File:Refugee_hotspot_Fessas_(3).jpg).

Figure 2. Camp locations and number of participants (n=12) with experience at each site.
Distribution of camps follows eastern entry migration routes into Europe, and clustering at the English Channel by those seeking to enter the UK. Left to right: Calais, France, 5 participants all

For peer review only

For peer review only - http://bmjopen.bmj.com/site/about/guidelines.xhtml

active at informal (IF) camps; Dunkirk, France, 3 all IF; Brussels, Belgium, 1 formal (F); Heumensoord, Netherlands, 1 F; Belgrade, Serbia, 1 IF; Macedonia, 1 IF; Patras, Greece, 1 IF; Idomeni, Greece, 1 IF; Thessaloniki, Greece, 3 – 2 F, 2 IF; Athens, Greece, 1 IF; Attica, Greece, 1 F; Lesbos, Greece, 3 –1 F, 2 IF; Çesme, Turkey, 1 IF; Samos, Greece, 2 F; Tilos, Greece, 1 F. (Figure uses adapted basemap by Julio Reis (2006) created using public domain mapping from the European Environment Agency, use and changes made under CC BY-SA 3.0 which also applies to this image. Original basemap: https://web.archive.org/web/20161019140631/https://commons.wikimedia.org/wiki/File:Europe_biogeography_blank.svg

For peer review only

For peer review only - http://bmjopen.bmj.com/site/about/guidelines.xhtml

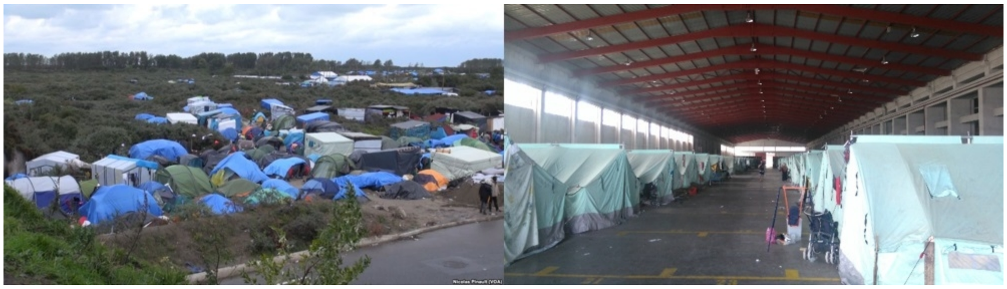

Figure 1. Informal and formal refugee/migrant camps
Left: informal camp in Calais, France, 2015 (VOA News - Nicolas Pinault (2015), public domain, original: https://web.archive.org/web/20210406154625/https://commons.wikimedia.org/wiki/File:Calais2015a.jpg).
Right: formal camp Thessaloniki, Greece, 2016 (Konstantinos Stampoulis (2016), used under CC BY-SA 4.0 which also applies to this figure, original: https://web.archive.org/web/20210406155208/https://commons.wikimedia.org/wiki/File:Refugee_hotspot_Fessas_(3).jpg).

263x74mm (96 x 96 DPI)

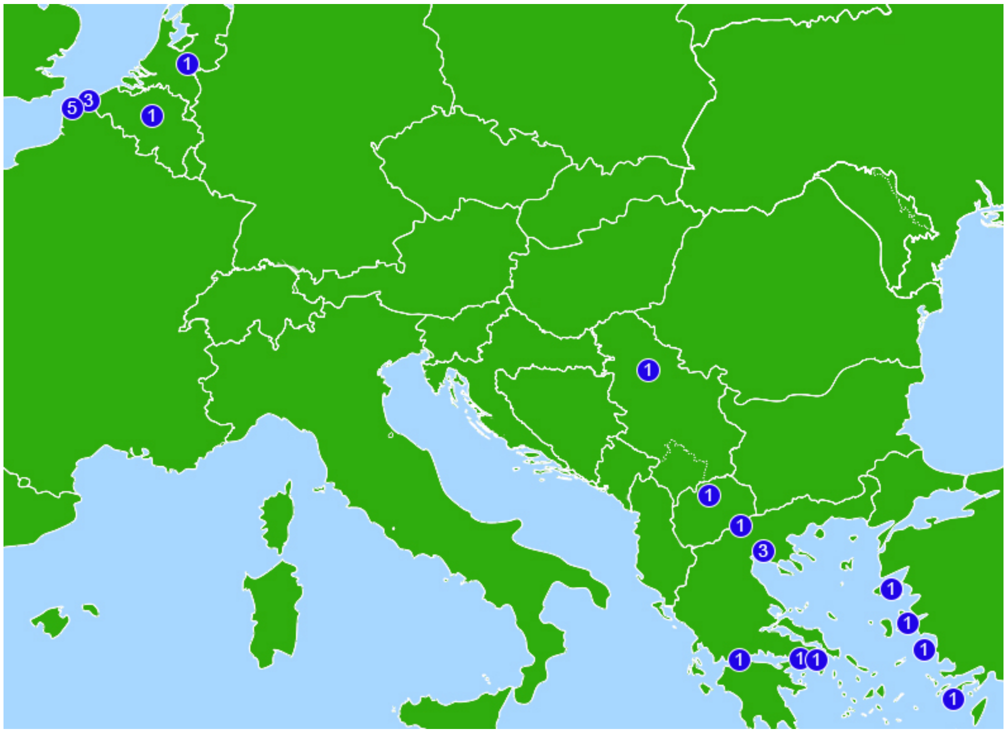

Figure 2. Camp locations and number of participants (n=12) with experience at each site. Distribution of camps follows eastern entry migration routes into Europe, and clustering at the English Channel by those seeking to enter the UK. Left to right: Calais, France, 5 participants all active at informal (IF) camps; Dunkirk, France, 3 all IF; Brussels, Belgium, 1 formal (F); Heumensoord, Netherlands, 1 F; Belgrade, Serbia, 1 IF; Macedonia, 1 IF; Patras, Greece, 1 IF; Idomeni, Greece, 1 IF; Thessaloniki, Greece, 3 – 2 F, 2 IF; Athens, Greece, 1 IF; Attica, Greece, 1 F; Lesbos, Greece, 3 –1 F, 2 IF; Çesme, Turkey, 1 IF; Samos, Greece, 2 F; Tilos, Greece, 1 F. (Figure uses adapted basemap by Julio Reis (2006) created using public domain mapping from the European Environment Agency, use and changes made under CC BY-SA 3.0 which also applies to this image. Original basemap: https://web.archive.org/web/20161019140631/https://commons.wikimedia.org/wiki/File:Europe_biogeography_blank.svg

80x58mm (250 x 250 DPI)

Online supplementary file

Scabies outbreak management in refugee/migrant camps in Europe 2014–17: a retrospective qualitative interview study of healthcare staff experiences and perspectives

NA Richardson, JA Cassell , MG Head, S Lanza, C Schaefer, SL Walker, J Middleton

CONTENTS

- Examples of news reports of scabies in refugee/migrant camps in Europe 2
- Supplementary Table 1: Facebook.com hosted groups in which the study was advertised 2
- Characteristics of researchers 2
- Supplementary Table 2: Standards for Reporting Qualitative Research checklist 3
- Supplementary Table 3. Participants' characteristics and settings 4
- MSF clinical guidance for scabies 5
- Themed quotations from interviews 6–21

For peer review only - http://bmjopen.bmj.com/site/about/guidelines.xhtml

Examples of news reports of scabies in refugee/migrant camps in Europe

Cooper C. Calais crisis: Migrants are living in appalling conditions, say doctors: Scabies is spreading like wildfire. The Independent. 26 September 2015, London. [Accessed 10 Nov 2017]. Available from: https://www.independent.co.uk/news/world/europe/calais-crisis-migrants-are-living-in-appalling-conditions-say-doctors-a6668631.html

Lichfield J. French police clear hundreds of asylum-seekers from Calais migrant camps after outbreak of scabies. The Independent. 28 May 2014, London. [Accessed 3 Jan 2019]. Available from: https://www.independent.co.uk/news/world/europe/french-police-clear-hundreds-of-asylum-seekers-from-calais-migrant-camps-after-outbreak-of-scabies-9444407.html

Newton J. Migrants living in Paris refugee camp often used as a stop-off before heading to Calais and Britain are forced to move on by police over fears they were sparking a scabies epidemic. Mail Online. 1 June 2015, London. [Accessed 3 Jan 2019]. Available from: http://www.dailymail.co.uk/news/article-3105501/Migrants-living-Paris-refugee-camp-used-stop-heading-Calais-Britain-forced-police-fears-sparking-scabies-epidemic.html

Supplementary Table 1.
Facebook.com hosted groups in which the study was advertised.

- Boat Refugee Foundation – UK Medical Recruitment
- Care 4 Calais Volunteer Chat Group
- CARE UK Charity (Refugee aid NW)
- Humanitarians in the UK
- Information Point for Lesvos Volunteers
- Junior doctors – refugee medical volunteers
- Medics for Refugees (former Medics for Greece)
- People to People Solidarity – Dunkirk and small camps
- RAISE – Refugee Action In Somerset East
- Solidarity for Refugees

Characteristics of researchers

NR is a global emergency medicine clinical fellow with a Diploma in Tropical Medicine and Hygiene, and training in interview skills and qualitative analysis. SL and JM are Brighton and Sussex Medical School staff with prior research into institutional scabies outbreaks [3, 10, 12, 14, 33, 38, 39]. JM has previously done voluntary NGO work with refugees, and lived in physically similar informal camps to those described in the study.

For peer review only

For peer review only - http://bmjopen.bmj.com/site/about/guidelines.xhtml

Supplementary Table 2.
Standards for Reporting Qualitative Research [24] checklist.

No.	Topic	Page/line no(s)
	**Title and abstract**	
S1	Title	1/2–4
S2	Abstract	2/25–51
	**Introduction**	
S3	Problem formulation	4–5/82–100
S4	Purpose of research question	4–5/96–105
	**Methods**	
S5	Qualitative approach and research paradigm	6/133, 138–140
S6	Researcher characteristics and reflexivity	5/111, 129; 20/446–59<br>Sup file 2/30–35
S7	Context	4–5/65–100; Figures 1 and 2<br>Sup file 2/14–27
S8	Sampling strategy	4/112–123;Sup file 2/Sup table 1
S9	Ethical issues pertaining to human subjects	16/410–411
S10	Data collection methods	5/108–110, 125–136
S11	Data collection instruments and technologies	5/128–129
S12	Units of study	6/150–160; Sup file/Sup table 3
S13	Data processing	6/137–138; Figure 2
S14	Data analysis	6/132–137, 138–144
S15	Techniques to enhance trustworthiness	6/142–144
	**Results/findings**	
S16	Synthesis and interpretation	6–12/152–275
S17	Links to empirical data	Tables 1–3; 9/198–209<br>11/246–249; 12/269–271<br>Sup file 6–21/61–723
	**Discussion**	
S18	Integration with prior work, implications, transferability and contribution(s) to the field	13–15/302–393
S19	Limitations	12–13/288–308
	**Other**	
S20	Conflicts of interest	16/422
S21	Funding	16/419–421

For peer review only

For peer review only - http://bmjopen.bmj.com/site/about/guidelines.xhtml

39 **Supplementary Table 3.**
40 **Participant characteristics and settings.**

**Sex**	
Female	10
Male	2
**Age**	
≤30	5
31–40	2
41–50	2
51–60	1
≥61	1
Unknown	1
**Type of healthcare staff**	
Doctor	4
Nurse	4
Allied health professional	3
Other (medical student)	1
**Number of years practising**	
1	1
2	2
5.5	1
11	1
12	1
22	1
24	1
40	1
Unknown	1
N/A	2
**Nation state of usual practice**	
UK	6
France	1
Belgium	1
Netherlands	2
India	1
USA	1
**Pay status**	
Volunteer	10
Paid staff	2
**Legal status of camp***	
Informal	9
Formal	6

41 ** n=12, total >12 as some participants worked in multiple camps*

For peer review only

For peer review only - http://bmjopen.bmj.com/site/about/guidelines.xhtml

MSF clinical guidance for scabies [30]

All cases:

- Close contacts treated simultaneously
- Clothing and bedding changed after treatment

1st line:

Permethrin 5% (lotion or cream)

Apply everywhere on the body, contact time of 8 hours then rinse

One application may be sufficient, second application 7 days later reduces treatment failure

Or **Oral ivermectin** 200mg single dose

Single dose sufficient, second dose 7 days later reduces treatment failure

Not for pregnant women or children under 15kg

If 1st line treatment unavailable:

Benzyl Benzoate 25% lotion

Diluted before application use based on age

Apply everywhere on the body, contact time of 24 hours then rinse (contact time reduced for infants/babies)

Second application reduces the risk of treatment failure (eg. after 24 hours, with a rinse between applications or two successive applications 10 minutes apart)

For peer review only

For peer review only - http://bmjopen.bmj.com/site/about/guidelines.xhtml

THEMED QUOTATIONS FROM INTERVIEWS

Quotes from the thematic framework matrix, extracted from verbatim transcriptions of 12 interviews, 34–71min each (mean 47).

Description of the Camp Environment

General

P1: "It was quite distressing to look at really, and people were exploited."

"There was quite a few cameras from the newspapers... which was quite intrusive"

P4: "Can you imagine like frontline war correspondent, you know just getting there or I know frontline medic you know in World War 2, bit like that without the bullets"

P7: "Very confusing, very high stress levels and very difficult. It was a transient camp, people came straight from the boats, the tents, they were hypothermic, it was winter, so it was extremely transient and extremely a difficult situation."

P8: "It's horrible. There's only one water source for over two thousand people, there's not enough clothes, people are sleeping outside, there's not enough tents, the hygiene is terrible. I mean I was there in the summer so it was hot, there's not enough shading and people couldn't find a place outside the sun. It's more horrible now, as its winter, so it's freezing, and there's no heating. To be honest it's worse than the refugee camps I've seen in Africa"

P9: "I think they are a human rights violation. I don't think people should be living in them, especially in a first world European country this should not be happening. People are living in cold tents without heating and electricity, there's rats, there's scabies, there's a lack of toileting facilities, a lack of hygiene, lack of food."

P10: "You don't see why everyone says it's horrible, it doesn't seem that bad when you first arrive, but when you start talking to people who live in these camps every single day, you see how it crushes them over time."

"The other thing that really struck me was how gender based vulnerability was accounted for in practice. When it snowed, they kept moving women out to a better and nicer camp, but men get cold to yet they only moved out the women. It sucks to be a man."

P11: "It was like an emergency pavilion... they put 3,000 refugees together in a big camp... Every tent, there were 10 people sleeping, and they went eating in a large room, where they could eat together with 100s of people. They have all the place where they can go to the toilet, and places where they can wash themselves, but it was all outside, it was inside they could sleep and eat, but they can wash themselves outside, and they could to the toilet. It was all arranged, but it was very basic."

Shelter

P1: "It's very organised... you think you're in a small village really... but then when you go beyond that you see just a sea of tents where people just live"

P2: "There's not even proper tents or anything, they just use whatever they can find"

"In Serbia it's almost like a jungle, so it depends on like how lucky you are. So, some refugees have tents, some doesn't have tent. And then sometimes they just sleep in the field because they walk to the border and sometimes they just stop in between and they sleep like under the stars."

For peer review only - http://bmjopen.bmj.com/site/about/guidelines.xhtml

P3: "There's no shelter... there's just literally people sleeping in bushes"

P4: "There are no more camps, they're gone... so we have like, nine hundred refugees living rough in Calais and then we have probably about four hundred and fifty refugees living in the forests of Dunkirk."

P5: "People live there with tents or without tents."

P7: "The camp was steadily improving by the volunteer and NGO work, and residents work, it was not as stressful environment as the factory hole which had been utilised in the first as tents... It was not a pretty environment but a lot of effort to make it a habitual environment and make it was a better environment in the end."

P12: "Different locations so it was the side of roads mainly, and next to the roundabout there would be a patch of grass and there would be a large group of people on the grass... then at another location might be in kind of a residential area but at the back of that there'd be a field, just some woodland and then there'd be a large group of people there as well so it was, it was groups of people scattered throughout Calais. It wasn't a camp"

Water and Sanitation

P1: "Running water was in troughs, like animal troughs. People washed in the open, or didn't wash at all. They didn't have any clothes washing; there was no way... People would just wash their clothes and just hang it over a bush or something"

P2: "There is no proper latrine system"

"In Serbia, there's one German NGO that are giving free showers every second day for refugees. They also provide some water so the refugees can wash their clothes and stuff. But, in general, in one setting they can only give showers to up to forty people max. So, there are like one hundred and fifty to two hundred people, they could only give showers to forty people. So, some people might not be able to do showers, like maybe you can do once in a week. The sanitation is quite bad, and even the water, sometimes there is no water, if this NGO doesn't come and provide water, so they do take water from some cart, but it's not really clean"

P3: "There are portable toilets there, but that's about it, really. Occasionally an organisation, like Calais government, kind of, a local authority, they occasionally bring, I guess you would describe them as, not showers, but more like taps, portable taps with cold water, so people could have a kind of a quick wash and things"

P6: "It was quite well established... having said that, facilities were still really quite basic. The toilets were just portaloos, there was very limited access to showers, and running water and it was very muddy"

P10: "I never stepped inside any of the facilities in the camps, I smelled them as I walked past but I didn't have an incentive to go inside so I didn't. I assume they were quite terrible, like I could smell them, but not good, I did not see them, they were not good, I didn't want to see them."

Food

P3: "There was the food distribution which was run by volunteers so there would be a van going twice a day to camps around Calais and once a day to Dunkirk. But apart from that, there's very little"

Safety

P1: "There was areas there where the women and children stayed that was quite well organised and had security and were patrolled... but a lot of people didn't want to go in there, and often families were separated"

For peer review only

For peer review only - http://bmjopen.bmj.com/site/about/guidelines.xhtml

P2: "Often the riot police, they're called the CRS, they have these big vans and they would often kind of hover around, trying to intimidate the refugees and the volunteers, and so that didn't kind of make for a very nice atmosphere it was quite tense"

P5: "It feels like the woman, she is not completely safe because they protect themselves, they are old, sometimes there are pictures of walls falling down. It's not safe also because there are a lot of tensions so sometimes fights happen."

P12: "People were hiding from the authorities... because tents were being routinely slashed and belongings were being destroyed by the police and authorities"

Healthcare

P2: "Those camps in Turkey, the settlements, they are really in bad shape, no NGOs are running in, no-one to give free food, no-one to give the healthcare. I mean we were there giving free healthcare, but basically we didn't go and visit them every day, so they'd basically sometimes end up not having at all, and they were refused to get healthcare in normal hospitals"

P10: "You start to see how someone with heat failure isn't able to access treatment - it's crushing him. A person with diabetes can't get their blood sugars up - its killing them."

"A lot of organisations can be disrespectful, especially the doctors, they can be very humiliating, that makes people reluctant to go back for treatment. And they can also be rather indifferent to a lot of things, which means things don't get taken care of. Refugees wait in line for several hours, to be seen, to be disrespected, so they don't go back to the doctors."

Barriers

Water, Sanitation and Hygiene Facilities

Washing of clothes

P1: "Clothes that they hadn't been able to wash, or they only came over in their clothes that they were standing in"

"There wasn't anywhere that people could wash their clothes, yeah, have washing facilities to wash their clothes... They wanted to be clean. And it's very difficult to keep clean if you're living outside in a tent"

P2: "Most of the scabies has gone so bad, the scar has got so infected because of the very poor clean and hygiene. They've been wearing the same pants, sometimes if it's raining you get wet and you don't have any new clothes so you just wear the wet clothes all over again."

"They might have some utensils to cook, but not enough to boil so much of water, or somewhere they can soak all the clothes and to wash them. And even if they hand it all to the volunteers, even we don't have the capacity to disinfect all the clothes"

"Sometimes they don't have water...it's quite tricky to wash the scabies infected clothes. If you have big washing machine that has a warm water setting, that would be great. But some of them doesn't have it, so you have to boil water and soak it or you have to put chemicals and stuff... the chemicals that were needed, you don't know where to get them, so you need to get support from the larger NGOs. If you don't have larger NGOs then you might not be able to do it"

P3: "People have so little and they don't want to give any clothes or bedding that they have up... definitely not clothes washing"

P4: "There was like cold taps there that they could wash with to wash it off the next day, but my problem with that was the clothes that they're putting back on. Yeah, the clothes have to be washed at a high temperature. So it's very much a struggle"

For peer review only - http://bmjopen.bmj.com/site/about/guidelines.xhtml

“the treatment we were giving was that because it was toxic we told them you have to wash it off the next day, that they could shower it off, but that’s all. They put back on infected clothes or they’d dry with infected towels”

P5: “Then we have other issues which is means we have to get clothes, new clothes for these people and new sleeping bags because, and then we cannot tell them to wash the sleeping bags in the residence at 60°, it doesn’t work because they’re homeless.... they can only wash their clothes by hand so of course they cannot wash everything that there is... we cannot provide clothes for all of them.”

P6: “Even if a patient is treated for scabies, part of the treatment is also to wash the bedding, and all clothing in hot water, dry it in a drier picking up all the scabies mites. And those facilities weren't readily available for anybody at the camp... I still think that even if we'd of had a sort of endless supply of Permethrin or Ivermectin, I think it still would have been problematic with this problem of re-infection.”

P8: “The challenge was to do the hygiene part of the treatment... The problem was to find enough clothes for them to change their clothes because there is no washing thing in the camp so we would have to do the whole thing of putting the clothes in a garbage bag, close it for 72 hours, then take it out. Then we actually found out that a lot of people don’t actually have a spare set of clothes, or they have a sweater and trousers but don’t have more underwear.”

“There’s only one water source for over two thousand people... there was no washing facilities for the people, they had to wash their clothes by hand. There was no soap provided. People did not have the possibility to really wash their clothes well.”

P9: “They couldn’t wash and we didn’t have enough blankets to give out, so everything was getting re-infected all the time. We couldn’t take everybody’s clothes because there wasn’t enough clothes to give out again so that was really hard to manage... Things were just getting re-infected because it wasn’t very well controlled.”

P11: “Insufficient washing and bedding, and like not enough washing machines... a lot of people, most of them only had one pair of clothes, and they can't change them, when they treat. We need clean clothes, so that was a difficult part... You also have to think about the hats, scarves, they also have to wash them, because in the beginning, that was a thing we didn't clean the hats, scarves.”

P12: “Most quite overwhelming was the lack of amenities, people’s clothing, sanitation facilities, so no showers, no toilets.”

Shower facilities

P2: “There are one hundred and fifty to two hundred people, but they could only give showers to forty people. So, some people might not be able to do showers… the sanitation is quite bad, and even the water, sometimes there is no water. If this NGO doesn’t come and provide water, so they do take water from some cart, like not really clean and it’s not really in a good shape.”

P3: “Not having access to showers”

“That would be the only way to get rid of scabies. But this doctor said that he was already, I mean it’s a small clinic, he was already overwhelmed, he was saying he’d have to… They only had two showers, there’s no way they could do that, he just wouldn’t be able to cope with that many people.”

“They don’t have showers, or they don’t let the refugees use the showers.”

“At least in Calais they have these kind of portable taps, shower things. That’s not provided in Dunkirk, the council in Dunkirk won’t provide that for the refugees. One of the main barriers we faced was getting proper showers to people, or people to the showers.”

For peer review only

For peer review only - http://bmjopen.bmj.com/site/about/guidelines.xhtml

P4: "Lack of showers and lack of laundry meant that you couldn't focus on one. Okay, here's a tube of scabies treatment, it's quite toxic so you put this on your body, rub it all over, twenty-four hours later you wash it off, but all your clothes have to be clean and they'll look at you like really how. It's a very, very uphill struggle to treat it and it's really prolific."

"They did have that facility [clothes washing] in the Dunkirk camp but, towards the end of the camp, before it burnt down, they stopped washing clothes and they stopped, they turned off the showers and then scabies started there as well... In the beginning in the Dunkirk camp it seemed manageable. When we were there we hardly saw any. It was under control, but then the minute you put people out there in the middle of a forest in filth and dirt."

P5: "There is really no shower and there are some areas that are a kind of shower area, but it's not really a shower and its cold water. Basically it's not helping at all... it's very, very difficult to stay clean."

P6: "Treating scabies was problematic because as you're probably aware, with the Permethrin treatment, the lotion, you're supposed to keep it on for 12 hours and then wash it off after a 12-hour period, and access to showers was not always that easy... I never actually saw the shower facilities there but I understand they were very basic and there were often very long queues for the showers. So it wasn't easy to shower."

P8: "There is not even one shower, there's no soap to wash yourself... people couldn't even wash their bodies with soap."

Sharing of belongings

P1: "Charities came in to give clothes, but the clothes weren't ever washed and when someone went across the border, tried to come into England, all their stuff was left, and whoever was in the tent just used that"

P2: "The main biggest problem is to get them to improve their own hygiene... to get them to clean themselves, and have each one have their own towel, and not to share them."

P8: "A lot of people basically only had one pair or underwear, or socks, or they couldn't wash their shoes... they usually only had one pair of blankets... There would always be something that wouldn't be replaceable... Because of lack of things for themselves, they keep borrowing each other's clothes and blankets."

Healthcare factors

Availability of medication

P2: "Limited availability of medications."

"In Turkey, we don't have big NGOs because they're not allowed to be… so that was a bit tricky. We need get all this medications on the camp by ourselves. It's also depends on what is in each country. In some countries they use Benzyl Benzoate, then some of the countries Permethrin, so you need to know which one that they use for that country and what's familiar to that country. Then you can source it locally... Some people smuggle it in the bag, and they took a flight and then they give it to the organisation. In the long run it's not going to work because when you run out and you change to different regimes that's a bit complicated, if you stick to one thing that's easier to source."

P3: "The medication was very expensive... there's a lot of people, nearly 1000 people there in Calais and Dunkirk, that's an issue."

P4: "We don't have the treatment for it, because we don't have the money to buy in the treatment."

For peer review only

For peer review only - http://bmjopen.bmj.com/site/about/guidelines.xhtml

P5: "Only one we can buy in Greece is the more difficult to apply and the one you have to apply many times.... so there is less chance of success of people really following how to do it."

P8: "We didn't have enough medicine most of the time... what we would do is write down their names and a week later if we got the medicine we would ask them to come back... Because we were short of pills we would just give it once."

"In the beginning they trusted us, after a while they didn't because we promised them treatment and we didn't give it."

P9: "Just didn't have enough treatment... we have to fundraise to buy it, and if we don't have funds we can buy it."

P10: "Permethrin in pill form [we presume they actually mean ivermectin], they don't sell that in Greece, that would be the most convenient way to provide medical treatment... X had a stock from Netherlands, but as soon as that ran out it was gone. The only treatment that is available is Benzyl peroxide, but you have to dilute that to make it the right concentration, and dilute it differently for children, then leave it on for 24 hours or at least overnight. There was only one pharmacy that would only sometimes have it in stock, so we had to arrange with them beforehand... The medication available is the least convenient medication you could use. If you could use a pill for everyone that would be so much easier."

P12: "The lack of cream or treatment... Because none had been donate. We didn't have any in the container, and we just weren't able to get hold of any. The only medication we had was what was in the container, which I believe was from volunteers bringing over all donations. It was quite limited really."

Lack of privacy for medical exams

P2: "Don't have any proper building, clinics set up. So, everything sees what we are doing, so if someone has to show something we don't have a closed examination room, we don't have a private examination... you can't really examine them, like you can't really touch and see in your own eyes, you have to rely on the photos because they're too shy to show it to you."

P4: "You're out there in the middle of, you know, you're not somewhere quiet and private you can't ask people to strip off."

P12**:** "If you were treating somebody, there certainly wasn't any privacy for people... you'd find there'd be sometimes, gathering around and watching you... amongst large crowd."

"I don't think we were openly saying to people, "Oh I think you've got scabies," just because we didn't want to say in large groups of people and I don't think we wanted to publicise that type thing because you don't want anybody to be offended or people, other people are listening."

Lack of staff with experience of scabies

P2: "Most of the Western doctors are European, they're not very used to seeing scabies in normal settings."

"They have that scabies and it gets really bad and infected, some people might mistake it for like normal infected things... they don't give the creams for scabies and stuff."

"In Greece I met a lot of UK doctors. I find most of them, they know scabies and stuff. But when I was working with in Turkey and in Serbia, I worked with people from Europe, like Austrian and Germans, and then some of them had never seen scabies before. Because it's not very common in these kind of areas... so in terms of that staffing, it's not really hard to get staff, but it's really hard to get staff that have seen or have managed scabies before."

For peer review only - http://bmjopen.bmj.com/site/about/guidelines.xhtml

P4: “Most of the other first aid people they’d never seen scabies in their life.”

P8: “We heard they say they haven’t seen scabies which could mean they don’t know how to recognise scabies or they don’t want to, so could mean the other doctors in the camp don’t know how to recognise scabies.”

“Many people came to volunteer only for a few weeks so a lot of them didn’t have much experience to recognise it.”

P10: “European doctors did not have a lot of experience with scabies, so I think a lot of them didn’t realise what they were seeing.”

“A lot of volunteers had never seen scabies before... volunteer based organisations take people that want to volunteer, but because they need people they’re not really in a position to screen them for the job. I’ve definitely noticed that there really isn’t a lot of experience of doctors with scabies.”

P12: “I don’t know if I ever seen scabies before so I felt quite inexperienced.”

Social factors

Language barrier

P2: When I was working with the Afghan refugees, one of the time we couldn’t get a proper translator... and Didn’t want to tell the other people they had scabies and what’s going on. So, they feel reluctant to comply, and they’re like no, it doesn’t work. If you don’t have proper translators to translate anything to you that also could be a big barrier to understand what’s going on really.”

“When I was working in Serbia we don’t have a proper translator, so we were using one of the refugees to translate for us, which is not very ethically correct I would say, because of the confidentiality issues”

P3: “There were a lot of people, their English wasn’t great and we rarely had someone on the team who could speak Arabic for example so sometimes it was difficult to diagnose someone... it was difficult for them to explain to us, and for us to understand exactly what the problem was.”

P4: “We didn’t have all translators... so that was an issue. We sometimes had to ask another refugees and call out for somebody that could come over and help and speak enough English to tell us what was wrong with this person... I couldn’t explain to him what I could do so it’d just be, just frustration.”

P6: “We didn't have proper translating facilities. Trying to explain all of what I've just explained to you about washing off the treatment, the lotion and washing bedding and clothing, even if that was all possible, explaining all that to people without proper translators was difficult.”

P8: “It’s hard, because we were a volunteer organisation we didn’t have official translators. We would always ask people in the camp to help us translate. Sometimes it was difficult to find translators in certain languages, like Kurdish. In the end that would mean that the Kurdish people have less access to our care than Arabic or Farsi speaking patients.”

P9: “We usually have translators... but you don’t always have the right translator.”

P11: “When you talk to 10 people at a time, and both women and men together, it's maybe difficult, and there are also people who had another language, and some languages are not known in other translator centre, so we couldn’t get a translation for them.”

P12: “Language barrier”

For peer review only

For peer review only - http://bmjopen.bmj.com/site/about/guidelines.xhtml

Stigma

P2: “To ask them to, not to share things with your friend, try not to sleep with them, I feel bad sometimes. They sleep together, they have like one blanket and all sleep like five people on the same blanket. So, when you say okay, you have this problem, you can’t sleep with your friend, it’s almost like giving them a stigma.”

P7: “Some people had social barriers, as in I am so clean and I don’t get this kind of thing... "I have eczema", or "I have dry skin" or this or that, "I don’t have scabies". Those were the people who often suffered for the longest... Possibly as a result of stigma surrounding scabies... Scabies and mites are not very pleasant things to have.”

P8: “didn’t want to tell the other people they had scabies... there is some sort of stigma of course if you have some sort of rash that you are not clean or something.”

P11: “it was difficult because the Syrian people looked at Eritrean people like they were dirty… because they had scabies.”

Non-compliance with treatment

P2: “This whole effort has to be put by the patient, and under these circumstances this patient has a lot … a lot other things to think about rather than this itchy thing ... The compliance is really hard, and also to get them to continue the treatment over a few days, also quite hard as well.”

P5: “You have to treat case by case explaining to them how to do the treatment with the message of giving them new clothes and sleeping bag and that are shown everything and even when we try to explain everything they don’t always do it correctly. That’s for a major issue because if the treatment is not done correctly then it won’t be effective.”

P7: “The one thing which we did encounter occasionally was people that didn’t listen to us, so I would say to put the stuff all over your body, head down to the toes and they got it everywhere... to do it all in one night and keep it on for so many hours and then they walk away and then only put a bit of cream here, and a bit of cream there, for a week and then they come back and it hasn’t got better.”

P8: “When they would hear about the whole treatment thing that their whole tent has to be treated or it doesn’t make sense, then they would say ok never mind and go away and don’t come back. Or people would come and say that their roommates don’t want to come, or they would bring their roommates and then the roommates would say oh this is too much work, I don’t have itching so I won’t take part in this.”

Population Mobility

P2: “You have to trace who might be the contact... in the refugee setting you have to also to treat patients that might be from the same family, and they just move around. You can’t really tell who has slept here, who has not slept here, who might possibly get it.”

P3: “Not being able to kind of, you know, everyone’s moving around Calais all the time, so not being able to kind of, I guess, not having the resources to be able to keep track of individuals with scabies.”

P5: “You have always new people coming in, and people leaving so it means that even the people treated, if they come to you and you treat them and then they disappear, not having any other treatment.”

“Making one day of treatment of scabies is not successful because the population keeps changing... all the energy and the money spent from the treatment day actually is a kind of a waste... in this population which is always changing, it is not a good use of resources because we won’t be able to properly medicate it.”

For peer review only - http://bmjopen.bmj.com/site/about/guidelines.xhtml

P7: "Often these [single young men] were the most transient people who would simply wander off at some point, and not come back for a second dose or not come back for collecting whatever they're supposed to collect"

P8: "The fact that people move in the camp is also a big challenge because even if you treat a whole container, if one person is put there by the police and if they say this person has to sleep there and this person has scabies then the whole effort is for nothing. So that's actually one of the biggest challenges, that people are moving all the time."

P11: "It was difficult in this setting, because you can see there are 3,000 people, and everywhere probably 10 people in and 10 people out... it was also very difficult to treat them well, because normally we have to treat everyone together, at the same time."

"It's difficult to treat everybody together, and there's nowhere to isolate people, a lot of mobility of people, and logistical problems with that."

Organisational Factors

Living conditions – dormitory style rooms

P2: "In the government run camps they usually have their own tent or their own cubicle but in, in the jungle settings most of them live in quite overcrowded, they all just live, sit together and stuff."

P3: "Barriers in terms of the crowded conditions... I think just in terms of people who are living close together so scabies is very easily spread."

P6: "People were living in fairly cramped conditions which obviously made transmission a high possibility."

P7: "The most difficult to treat was the single young men, of being in the large accommodation, and camp accommodation, while the families, were in the family rooms. The single young men were in large dormitory style rooms and so it was a lot more difficult to determine the close contacts."

P8: "A lot of people were sharing tents or containers with large number of people."

P10: "Sometimes you would have people living in bigger units... and people would partition of with blankets where different families lived. That was really difficult."

Poor coordination between organisations

P5: "I remember one guy that they sent with everything for the scabies treatment and because it was my first day I was really trying to do it the way it should be done... He had an appointment for the day, so I wrote on a bit of paper and I wrote please allow this man to have two showers because he needs to do the lotion and wash it, 8 hours later. I sent him with a paper and everything. He had the new clothes, to bring back. Then they didn't allow him to have the first shower because he had scabies. They sent him away because he had scabies, even though he was coming to get the treatment."

P8: "A lot of things are managed by volunteers. Ideally in the camp you would want to have one named medical actor providing healthcare and asking for specialist to come and do clinics or to ask for advice from other organisations but there was no governmental healthcare facility. There was a huge gap because it didn't even exist, so other organisations would come in and do little things but the whole coordination between us and the others were so strange... there's no continuity. It was really unorganised."

"So many different organisations with people coming and going, also the paid staff did not stay longer than 6 months to one year, and so many volunteers coming and going - there's no continuity. It was really unorganised."

For peer review only - http://bmjopen.bmj.com/site/about/guidelines.xhtml

For peer review only

P10: “Lack of coordination and appropriate communication. I don’t think it’s the resources, the resources are there they're just not put together in the right way.”

“Topical steroids was what was given for symptom relief because they couldn’t give anything else, proper treatment required too much coordination.”

“The medical organisation in Moria didn’t communicate with non-food item NGOs, so due to lack of coordination he could never have medication at a time that he wouldn’t be at risk of re-infection... X used to write prescriptions for non-food items that weren’t honoured by the non-food item NGOs.”

P11: “There was another NGO who worked there, and sometimes they did their own things, their own plan. Sometimes it didn't work well together.”

P12: “Clothing was distributed, but we weren’t really able to coordinate that because we, we didn’t really have communication between the individuals and the charity.”

Lack of support and poor treatment from authorities
P1: “There wasn’t anybody in charge of this camp, to tell people that there was a scabies.”

“They were hidden, you know, thousands of people hidden in one corner of France that one-one wanted, the whole world didn’t want to recognise.”

“Because the French Government didn’t want the camp there, they weren’t doing anything. There wasn’t even... basic facilities... They didn’t really want to provide healthcare for people. There wasn’t any shelter, there wasn’t any child protection, there wasn’t any protection for any vulnerable people there at all, adult protection, there wasn’t any of those type of things there.”

P2: [informal camps] “What the government is trying to do is, they’re trying not to get the refugees too comfortable, so sometimes they cut like water, they try not to get us to be able to give out clothes, they raided our distribution place.”

P3: “I mean a lot of people avoid going to anywhere where they’ll interaction with the authorities because, because of the police brutality, they’re worried about how they’re going to be treated and worried about being deported. Even though that can’t happen at hospitals and things, and people at this particular clinic, you know, they’re running it for people like this and they’re very nice, but there’s still that perception. I think a lot of people try to avoid going.”

“The refugees have their bedding and their coats and shoes confiscated by police at night.”

“We had to take someone to A&E once, and he was treated awfully by staff because he was a refugee... what we saw it was just the refugees being stigmatised at being refugees.”

P4: “In France you’re forbidden by law to give first aid, well not give first aid, but to give prescription medication, my hands were very tied into how to treat it.”

“Until authorities do the right thing and take care of these people properly you can never, will eradicate it. It will carry on as long as they can’t shower properly, as long as they can’t get clean clothes and wash the clothes the scabies won’t go away and the trouble is that the authorities in France and in Greece and even in Italy do not care and they’re not bothered and they don’t wanna know and basically refugees are depending on volunteer groups and small charities for their wellbeing and they’re very limited.”
P6: “Local authorities were not getting involved because the camp was informal.”

P8: “This official organisation that was responsible for that kind of camp, there was one doctor who was responsible for the public health in the camp, he was responsible but most the time he wasn’t

For peer review only

For peer review only - http://bmjopen.bmj.com/site/about/guidelines.xhtml

there, he and the other organisation were ignoring that there was scabies in the camp... The Greek doctor kept saying it's not a life-threatening disease, yes its annoying but there are worse things to care about, but even the worse things weren't cared about... The people responsible for it would try to ignore it to ensure that they won't have to deal with it... in the meantime there was lots of suffering."

The care in the camp was very limited. In general, the government would be responsible to provide the healthcare but the government wasn't there so they had contracted [healthcare] out... but they were always short of staff and short of a lot of things.

P9: "In the official camps if scabies happened it would be much easier to deal with because they have some washing facilities and they've got like WaSH and that kind of organisations working there. I was working more in non-official, and where it was happening more for me was nonofficial and we didn't have that support."

P10: "Lack of will and commitment."

"A lot of organisations can be disrespectful, especially the doctors, they can be very humiliating, that makes people reluctant to go back for treatment. And they can also be rather indifferent to a lot of things, which means things don't get taken care of. Refugees wait in line for several hours, to be seen, to be disrespected, so they don't go back to the doctors."

P11: "They were just logistical problems, sufficient manpower, sufficient washing machines, sufficient bedding, who cares for this? It certainly wasn't a priority for the organisation, because people have itching, but they are not going to die"

"The responsible authority, the central body for reception and asylum seekers... they don't support it."

P12: "People were hiding from the authorities... the settlement or their place where they would sleep would not be there because tents were being like routinely slashed and belongings were being destroyed by the police and authorities."

"What would have made it easier to manage?"

P1: [Washing facilities]

"I think it [washing facilities] would have made it easier to manage, because people could wash their clothes, and the people wanted to wash their clothes as well. They wanted to dry them, they wanted to wear clean clothes. They wanted to be clean. And it's very difficult to keep clean if you're living outside in a tent."

P2: [Washing facilities; Public Awareness; Divided living areas]

"If you have a proper washing machine that have hot water then you can do it, or you can ask the refugees to do it themselves, but if you don't have that then it's really hard to be able to eradicate it."

"It's not hard, as long as you educate them and try to make them understand that what's going on, this is what needs to be done."

"For public health education you'd probably want to print out like a really easy version for it, or maybe you have to make a new one so they can hand out to the refugees to say we have these issues, and this is what happens if you are itching."

"If you have like a proper segregations of rooms, or cubicles for each family, that will be a really good point to really manage it. You can just do entire families. It's easier to also plot where the scabies is, you can trace them, where they go and what the patterns... But in the jungle they just get it like a wildfire."

P3: [Coordination of organisations; Access to resources]

For peer review only - http://bmjopen.bmj.com/site/about/guidelines.xhtml

"If we could kind of put a system in place, if we worked with X, one of the main organisations at Calais, they have a warehouse and do clothes distribution... to see if we could organise, maybe have a ticket system so organise getting people treated at the clinic so with cream or tablets, and then on the same day giving them a shower, clean clothes, taking away the old clothes to burn... giving them clean clothes, new clothes straight away and giving them a ticket so they can come back the next day for another shower 'cause you need to, and then to completely get rid of it, and making sure everyone in the camp does that.

"Access to resources... showers, clean clothes, and cream."

P4: [Better living conditions; Washing facilities]
"If they had somewhere decent to live... If they had somewhere with a roof over their heads and if they had somewhere where they could have a hot shower and somewhere to wash their clothes."

"I did think at one point I wonder if I could get like a mobile clothes washing van or something, it crossed my mind that it could be one thing we could have done with the treatment - get the clothes washed, shower, clothes washed, but it didn't happen."

P5: [Screening on arrival; Washing facilities]
"When we have treatment and sleeping bags and clothes, it's easier to manage because at least we can do something."

"The only measure that you could take is to take every new arrival and any sign of scabies and treat them, but it would take a lot of time to get sorted to do it. It is what they are doing in Serbia where all new arrivals with scabies, is quite a lot. We give all our new clothes and sleeping bag."

"If we could have a place to send people where there is a washing machine and dryer and maybe a shower, in this case we could treat more because we could have, like the new clothes but if all clothes that people wear, and the sleeping bags and blankets."

P6: [Oral medication]
"Availability of Ivermectin may have, to some extent because it doesn't require washing the lotion off afterwards, you know it's an oral treatment, a single dose oral treatment, may have helped but it wouldn't have really have overcome the problem of reinfection."

P7: "Nothing in particular - it wasn't a problem."

P8: [Screening on entry; Cooperation of organisations]
"In every camp where people arrive there's always a medical check for all the people so that would be a great time to check everybody for it, before they allocate them in a tent."

"Cooperation of the people that manage the camp because they could help by not moving people all the time, or always discuss it with a public health responsible person in the camp, but there was no such person. Making sure to allocate people in the right place to not disturb the scabies treatment, that would be great. Working together, putting together resources, but the first thing is to recognise that there is a problem."

P9: [Better living conditions]
"They shouldn't be living in these conditions in the first place. They should be in homes and houses, not refugee camps and abandoned buildings."

P10: [Interpersonal communication; Coordination of organisations]
"I think it's really important to train humanitarian workers in communication and interpersonal interaction... You have to really care, you have to understand that the other person isn't always going

For peer review only - http://bmjopen.bmj.com/site/about/guidelines.xhtml
For peer review only

566 to understand right away and you have to work with them... There's a lot of conversations that need to
567 be had."

568 "You need to appreciate what others in your NGO and what other NGOs are doing, and be conscious
569 of the fact that it is a collaborative goal."

570 P11: [More resources; Systematic treatment]
571 "If we had the time, the people, and just one tent more, then it would be no problem anymore because
572 you had to do one day, very intensive, and a good job, and then you have not left problems... The plan
573 I made I think it would be good, if there was money for it, if there were the people for it, and if there
574 were an extra tent over there, then it was managed in one day, in two days. Then we would have to
575 keep doing it, that every person who came in, and had the same treatment. You had first to do it with
576 all the people who were there, Eritrean people, and after that you had to do every Eritrean who came
577 in, we had to give them treatment, and then the problem was solved."

578 P12: [Coordination of organisations]
579 "We could organise what the warehouse felt getting them fresh clothes, but then we'd have to also
580 organise things like showers."

581 **Facilitators**
582 P3, P4, and P12: "nothing"

583 *Water, Sanitation and Hygiene*
584 P5: "What was really helpful is that the centre where people could go with a shower, and have a
585 washing machine... they can get a hot shower two times a week and they can also wash their clothes
586 over there. I mean if we provide one set of new clothes, we can help them to wash the clothes they
587 have. Twice a week is not enough to really make the treatment as it should be, but it is very good,
588 because before we had only places with appointments for the shower, there was only one shower.
589 There we can be sure they have their follow up of 10 days so they can get the treatment, the new
590 clothes, the new sleeping bag and take to the shower."

591 P7: "People have access to showers so that was not a problem, clothes had to be washed in the
592 washing machines… and bed sheets and everything else was taken out and burnt. That was the
593 protocol and we didn't have a persistent problem, we didn't have outbreaks."

594 P9: "There was mobile showers we were getting access to, not everybody could get access all the
595 time, so that was still difficult but there was that there so it's better than nothing."

596 *Coordination of organisations*
597 P1: "What we suggest we do is that they have clean clothing ready so that when they go to the
598 Government organisations, health organisations for the scabies, that they can actually start treatment
599 that evening, and we explain how they do that."

600 "We referred a few people to A&E because they were quite unwell. We gave letters to people about
601 scabies to bring to the medical place the next day. We made sure everyone had a letter saying it was
602 scabies, and documented when it was and how many people were in their tent etc."

603 P2: "We worked a team that provide showers. On one of the days, we call it a scabies shower, so
604 where we have all the scabies patients. We give them a card that says if you have scabies you get
605 showered tomorrow. We give them a card that says they have scabies, we give them a cream 24 hours
606 before and then asked them to get showers, and then we put the creams again. So we partner with
607 shower teams, and also, we partner with people from the distribution team so we could get them new
608 fresh clothes. I think the treatment of scabies, it has to be like not just a healthcare provider, and it has
609 to be like non-NGOs to help you work on that as well."

For peer review only - http://bmjopen.bmj.com/site/about/guidelines.xhtml

"It's really good to liaise with the large NGOs, that could provide, most of the time they [medications] were available. They have all those creams."

P3: "NGOs were more of a help, as I said X said that they could provide clean clothes and they could work with us."

P5: "Make a letter for them saying that this person has been diagnosed with scabies, please wash his clothes, allow him to have a shower and we will make a thing that it's not as contagious and that you cannot catch it like this. It's working well."

P7: "There was the camp administration which was run by a local NGO and the staff were aware of the problem and were good at directing people to us if there was a problem... we gave the patients who were referred slips to take to the administration to get new blankets, new bedding and that and take the old stuff away for washing or burning and give access to machines for to the washing."

"They have no difficulties staying on top of it, because the camp administration are behind it and the residents are behind it... That was all positive... the support from the camp administration."

P8: "The willingness of other organisations like the warehouse on lesbos that provided us with clothes."

P10: "Rented them clothes... built up an inventory of clothes, I worked with contacts in the warehouses to get donations so we could ship them over so we could lend them clothes based on their size/gender etc."

P11: "We had a meeting with them [NGO]... they had their own plan, and it was different from our plan. But we talked about it, and then it's okay. We gave everyone a clear task and then the problem was solved."

Education

P1: "We explain how they do that [the treatment] … and then for those that have been infected, for them not to shake hands, not to share bedding."

P2: "If you educate the patient very well, make them understand very well what's going on, and what needs to be done, I think that also worked very well... They understand, and they start to even manage to recognise their friends who have the same thing, which is really useful."

P3: "Friends might have been able to tell each other. I don't know help diagnose each other or encourage them to go to the clinics."

P7: "One treatment which was once easily explained and where the neighbours could explain to you, and everyone could explain to each other and reinforce the message which we gave every day."

Language facilitators

Translators

P1: "We had lots of translators there. We had our own translator who spoke several languages, and there were people who lived in the camp, working or volunteering as translators, and they were actually very good. Certainly, got the message across. So, there wasn't a barrier."

P3: "Some people did speak very good English and often if someone didn't, they would get someone who they knew did speak English and they would come and translate, so that helped."

P6: **"**Sometimes there were people that helped with translation, other occupants of the camp, other migrants would help with translating, but that wasn't consistent help it was just if they were around at the time and they offered to stick around to help a little bit.... if we did have people to translate then we were able to explain what the probable cause was of the itching and I suppose in itself having an

For peer review only - http://bmjopen.bmj.com/site/about/guidelines.xhtml

explanation can offer some relief to people, and also reassurance that there wasn't anything serious in terms of, you know life threatening."

P7: **"**They had very good translators for the other doctors and nurses."

P8: "A lot of people came to help us translate... The few people who spoke English would always be after other people to translate. Many people don't have anything to do all day so they'll help to translate for other people."

P9: **"**There's a range of languages, if somebody knows both they can tell us a bit more."

P10: **"**We were very lucky because X worked with unofficial translators from every community in the camp, so they were a great facilitator for getting the message across."

P11: "The language is difficult, but we talked with the translator, with a live translator."

P12: "There were a number of people there who had been residents in the UK but then had been deported so often there was somebody who could speak really, really good English. They would act as a translator and that was really helpful."

Voice messages
P5: "Voice messages in different languages."

"I tried to treat and tried to improve the way we are treating, to make it clear for the people in their language. So we have voice messages explaining how to take their cortisone treatment and how to apply the treatment. Basically for each person that we treat, we say in the voice message and ask which one [language] they prefer and then we play the voice message."

Information sheets in different languages
P5: "We do have information sheets... we show them with the data, and the map to go to the shower place and there if this is written in Urdu, then we will have some positive treatment."

Incentive to take part in treatment
P2: "The best way that we come up with is just get them to get rid of whatever clothing and bedding, and then we give them replacements back. So there's an exchange there. That's the only way that we can minimise the spread and stuff."

P7: "The patients knew that it could be treated, some used it possibly as a way of getting new things and were quite disappointed if they didn't have it, but that didn't happen that often either."

P8: "Some people when they found out we would give them another set of clothes and we would buy underwear for them, some more people were interested as they had an incentive to take part in it."

P10: "We would take back everything except the underwear, so they got to keep the underwear, which for a lot of people was like an incentive to participate."

General
Division of living areas
P2: "If you go for like a government run camp you have proper dividing, like in Northern Greece, they have cubicles. So, usually the outbreaks are a lot smaller and usually limited to a few cubicles, or maybe just one cubicle."
"If you have like a proper segregations of rooms, or cubicles for each family, that will be a really good point to really manage it. You can just do entire families. It's easier to also plot where the scabies is, you can trace them, where they go and what the patterns... But in the jungle they just get it like a wildfire."

For peer review only

For peer review only - http://bmjopen.bmj.com/site/about/guidelines.xhtml

P7: “People were living in individual, family individual cells, not exactly houses, not exactly rooms… Anyway there was some level of physical insulation from family to family and so you could say this family is all close contacts and it gives you good conscience to clear other people like neighbours, declare them as neighbours, but not close contacts.”

Technology
P2: “We don’t have a closed examination room... But some refugees are quite smart, so they bring in photos.”

Donations
P3: “We managed to raise money for the cream... we have managed to raise quite a lot, well not a lot, but some money to be able to buy medication.”

“There would be donations of clothes, quite often from the main two warehouses.”

P5: “When I got the donation of ivermectin coming, it was good. I was so happy, I think the person who was leaving me so meds, she didn’t know what happened, I was really quite… I knew that it was so much easier to treat scabies.”

P8: “Our organisation runs on donations so if we did have a donation then we would be able to buy medicines.”

P9: “Fundraising for medicines.”

P10: “We had a stock [medication] from the Netherlands from donations.”

Guidance
P7: [Treatment protocol]
“The protocols worked well... The ability to pass on a systematic and well-rehearsed message.”

“One treatment which was once easily explained and where the neighbours could explain to you, and everyone could explain to each other and reinforce the message which we gave every day... We had one treatment which fits into the medical centre and one treatment which was approved by the different governments and that treatment is positive.”

P10: [staff advice/screening documents]
“Screening questionnaire that X staff could use to screen people, which was really important as a lot of volunteers had never seen scabies before... it meant that a lot of people could do the counselling, and tell people to get seen and at the time when the medication was being distributed the one or two doctors who had experienced seeing it would be there, but in the meantime it gave everyone else a tool to find household who had members suffering from scabies.”

For peer review only

For peer review only - http://bmjopen.bmj.com/site/about/guidelines.xhtml

BMJ Open

Scabies outbreak management in refugee/migrant camps in Europe 2014-17: a retrospective qualitative interview study of healthcare staff experiences and perspectives

Journal:	*BMJ Open*
Manuscript ID	bmjopen-2023-075103.R1
Article Type:	Original research
Date Submitted by the Author:	03-Oct-2023
Complete List of Authors:	Richardson, Naomi; Brighton and Sussex Medical School, Primary Care and Public Health, Watson Building, University of Brighton<br>Cassell, Jackie; Brighton and Sussex Medical School, Primary Care and Public Health, Watson Building, University of Brighton<br>Head, Michael; University of Southampton, Clinical Informatics Research Unit, Faculty of Medicine<br>Lanza, Stefania; Brighton and Sussex Medical School, Primary Care and Public Health, Watson Building, University of Brighton<br>Schaefer, Corinna ; CADUS e.V<br>Walker, Stephen; London School of Hygiene & Tropical Medicine, Faculty of Infectious and Tropical Diseases; University College London Hospitals NHS Foundation Trust, Hospital for Tropical Diseases, and Department of Dermatology<br>Middleton, Jo; Brighton and Sussex Medical School, Primary Care and Public Health, Watson Building, University of Brighton; Brighton and Sussex Medical School, NIHR Global Health Research Unit on Neglected Tropical Diseases, and NIHR Applied Research Collaboration Kent, Surrey and Sussex
**Primary Subject Heading**:	Public health
Secondary Subject Heading:	Dermatology, Epidemiology, Global health, Infectious diseases, Qualitative research
Keywords:	Infectious diseases & infestations < DERMATOLOGY, International health services < HEALTH SERVICES ADMINISTRATION & MANAGEMENT, Infection control < INFECTIOUS DISEASES, Mass Drug Administration, Neglected Diseases, QUALITATIVE RESEARCH

SCHOLARONE™
Manuscripts

I, the Submitting Author has the right to grant and does grant on behalf of all authors of the Work (as defined in the below author licence), an exclusive licence and/or a non-exclusive licence for contributions from authors who are: i) UK Crown employees; ii) where BMJ has agreed a CC-BY licence shall apply, and/or iii) in accordance with the terms applicable for US Federal Government officers or employees acting as part of their official duties; on a worldwide, perpetual, irrevocable, royalty-free basis to BMJ Publishing Group Ltd ("BMJ") its licensees and where the relevant Journal is co-owned by BMJ to the co-owners of the Journal, to publish the Work in this journal and any other BMJ products and to exploit all rights, as set out in our licence.

The Submitting Author accepts and understands that any supply made under these terms is made by BMJ to the Submitting Author unless you are acting as an employee on behalf of your employer or a postgraduate student of an affiliated institution which is paying any applicable article publishing charge ("APC") for Open Access articles. Where the Submitting Author wishes to make the Work available on an Open Access basis (and intends to pay the relevant APC), the terms of reuse of such Open Access shall be governed by a Creative Commons licence – details of these licences and which Creative Commons licence will apply to this Work are set out in our licence referred to above.

Other than as permitted in any relevant BMJ Author's Self Archiving Policies, I confirm this Work has not been accepted for publication elsewhere, is not being considered for publication elsewhere and does not duplicate material already published. I confirm all authors consent to publication of this Work and authorise the granting of this licence.

For peer review only

[4993 words after reviewer suggestions; 3 tables, two figures; online supplementary file]

Scabies outbreak management in refugee/migrant camps in Europe 2014–17: a retrospective qualitative interview study of healthcare staff experiences and perspectives

Naomi A Richardson[1], Jackie A. Cassell[1], Michael G Head[2], Stefania Lanza[1], Corinna Schaefer[3], Stephen L Walker[4,5], Jo Middleton[1,6*]

[1] Department of Primary Care and Public Health, Brighton and Sussex Medical School, Watson Building, University of Brighton, Falmer, UK

[2] Clinical Informatics Research Unit, Faculty of Medicine, University of Southampton, Southampton, UK

[3] CADUS e.V., Berlin, Germany

[4] Faculty of Infectious and Tropical Diseases, London School of Hygiene & Tropical Medicine, London, UK

[5] Hospital for Tropical Diseases and Department of Dermatology, University College London Hospitals NHS Foundation Trust, London, UK.

[6] NIHR Global Health Research Unit on Neglected Tropical Diseases, and NIHR Applied Research Collaboration Kent, Surrey and Sussex, Brighton and Sussex Medical School, Watson Building, University of Brighton, Falmer, UK.

*Correspondence: Jo Middleton, Department of Primary Care and Public Health, Brighton and Sussex Medical School, Watson Building, University of Brighton, Falmer, BN1 9PH, UK.

Email: j.middleton@bsms.ac.uk

https://orcid.org/0000-0001-5951-6608

Twitter: https://twitter.com/MedVetAcarology

Keywords: Infectious diseases & infestations < DERMATOLOGY; International health services < HEALTH SERVICES ADMINISTRATION & MANAGEMENT; Infection control < INFECTIOUS DISEASES; Mass Drug Administration; Neglected Diseases; QUALITATIVE RESEARCH

For peer review only

For peer review only - http://bmjopen.bmj.com/site/about/guidelines.xhtml

ABSTRACT

Objectives. Provide insights into the experiences and perspectives of healthcare staff who treated scabies or managed outbreaks in formal and informal refugee/migrant camps in Europe 2014–17.

Design. Retrospective qualitative study using semi-structured telephone interviews and framework analysis. Recruitment primarily through online networks of healthcare staff involved in medical care in refugee/migrant settings.

Setting. Formal and informal refugee/migrant camps in Europe 2014–17.

Participants. Twelve participants (four doctors, four nurses, three allied health workers, one medical student) who had worked in camps (six in informal camps, nine in formal ones) across 15 locations within seven European countries (Greece, Serbia, Macedonia, Turkey, France, the Netherlands, Belgium).

Results. Participants reported that in camps they had worked scabies diagnosis was primarily clinical (without dermatoscopy), and treatment and outbreak management varied highly. Seven stated scabicides were provided, five that only symptomatic management was offered. They described camps as difficult places to work, with poor living standards for residents. Key perceived barriers to scabies control were (i) lack of water, sanitation and hygiene, specifically: absent/limited showers (difficult to wash off topical scabicides); inability to wash clothes and bedding (may have increased transmission/re-infestation), (ii) social factors: language; stigma; treatment non-compliance; mobility (interfering with contact tracing and follow-up treatments), (iii) healthcare factors: scabicide shortages and diversity; lack of examination privacy; staff inexperience, (iv) organisational factors: overcrowding; ineffective inter-organisational coordination; lack of support and maltreatment by state authorities (e.g. not providing basic facilities; obstruction of self-care by camp residents and NGO aid).

Conclusions. We recommend development of accessible scabies guidelines for camps, use of consensus diagnostic criteria, and oral ivermectin mass treatments. In addition, as much of the work described was by small, volunteer staffed NGOs, we in the wider healthcare community should reflect how to better support such initiatives, and those they serve.

For peer review only - http://bmjopen.bmj.com/site/about/guidelines.xhtml

STRENGTHS AND LIMITATIONS OF THIS STUDY

- Recruitment through online healthcare staff networks enabled collection of the subjective perspectives of a participant population difficult to reach through formal means, given many had volunteered with NGOs but were not necessarily still anchored to them.
- Telephone interviews prevented observation of non-verbal cues, but did facilitate international involvement in a timely and resource-efficient manner.
- The relatively long and semi-structured nature of the interviews gave participants opportunity to articulate their perspectives, to an extent unconstrained by researcher presumptions over what may have been important to them about their experiences.
- Conducting data collection and thematic analysis simultaneously enabled assessment of data saturation during recruitment, but advertising and interviewing solely in English excluded experiences from non-English speaking individuals.
- The retrospective nature of the study may have introduced recall bias.

For peer review only

For peer review only - http://bmjopen.bmj.com/site/about/guidelines.xhtml

INTRODUCTION

Scabies is a stigmatised contagious skin condition caused by infestation with the mite *Sarcoptes scabiei.*[1] Transmission is mainly skin-to-skin, less commonly via fomites such as bedding.[2] Evidence from epidemiology and experimental trials indicates scabies prevalence is probably not influenced by levels of personal body washing.[3] Symptoms begin 3–6 weeks after first infestation, as early as one day after re-infestation.[4] Secondary bacterial infections are common,[5,6] with potential for serious long-term health impacts, including chronic rheumatic heart disease and chronic kidney disease.[7–9] Diagnosis is normally by clinical examination, sometimes using dermatoscopy.[4, 10] However, definitive confirmation of diagnosis, according to consensus criteria of the International Alliance for Control of Scabies (IACS), requires 'A1: Mites, eggs, or feces on light microscopy of skin samples; A2: Mites, eggs or feces visualized on individual using high-powered imaging device; A3: Mite visualized on individual using dermoscopy' (see Box 1, Summary of 2018 IACS criteria for the diagnosis of scabies). Simultaneous treatment for diagnosed individuals and close contacts is required.[11] This normally consists of topical scabicides applied to the entire body or oral ivermectin, in both cases often given twice (one week apart).[12] Environmental decontamination advice varies.[12] WHO designate scabies as a neglected tropical disease (NTD), with an estimated 455 million cases annually.[13] It's highest burden is in the tropics, but in lower-burdened regions (such as Europe) scabies remains common, emerging as a public health problem when institutional outbreaks occur.[10,13,14]

Over the last decade conflicts triggered large numbers of people to migrate to Europe.[15–17] By the end of 2016 European countries hosted 5.2 million refugees, 2.9 million in Turkey alone (mostly from Syria).[15] As a result, during 2014–17 many formal refugee/migrant camps and reception centres, and informal (often illegally occupied) camps came into being or expanded (figure 1). Healthcare services in camps were often limited to clinics operating from caravans or makeshift structures, largely staffed by volunteers; better resourced camps could still face shortages and overcrowding following unexpected influxes.[16, 18] Scabies has been one of the most frequently observed medical conditions in these settings.[18–22] In Germany, it was the third commonest outbreak type in asylum seeker shelters.[21] In France, Doctors of the World UK estimated in 2015 that up to 40% of those seeking their care in the Calais 'Jungle' camp had scabies (Cooper, online supplementary file, p.2). Outbreaks have been widely discussed in news and social media. Media analysis by Seebach *et al*.[23] described some of this coverage as stigmatising those affected by scabies, whilst positing migrants/refugees more generally as an invasive health threat to European countries. Multiple camps in France have been evicted with the public justification of responding to scabies outbreaks (examples of newspaper reports: Lichfield; Newton, online supplementary file, p.2). Despite all this, to our knowledge no study prior to ours has been published specifically on scabies outbreak diagnosis,

For peer review only - http://bmjopen.bmj.com/site/about/guidelines.xhtml

treatment, and management in refugee/migrant camps in Europe during this period. Further, we are unaware of any published study on healthcare staff perspectives on barriers and facilitators to treating and managing scabies in refugee camps globally.

Aims

This qualitative study aimed to provide insights into experiences and perspectives of healthcare staff who treated scabies or managed associated outbreaks in refugee/migrant camps in Europe 2014–17. Specifically, to describe: (i) methods used to diagnose, treat, and manage scabies; (ii) camp characteristics and perceived barriers and facilitators to effective scabies outbreak management.

METHODS

Study design

We carried out a retrospective qualitative study using semi-structured telephone interviews and framework analysis. Recruitment was through online networks of healthcare staff active in refugee/migrant settings. Reporting is in line with the Standards for Reporting Qualitative Research [24] (checklist (supplementary table 1) and researcher characteristics, online supplementary file).

Participant selection and recruitment

Healthcare staff from state and non-governmental organisations (NGOs) who had treated scabies in refugee/migrant camps in Europe, and/or managed associated outbreaks in the previous three years were eligible. We recruited by contacting:

(i) relevant healthcare staff known to us;

(ii) individuals publishing research on medical care in refugee/migrant settings;

(iii) through online networks of healthcare staff active in refugee/migrant settings, including the alumni network of the European Centre for Disease Control fellowship program and groups hosted on facebook.com. To target the latter a public page (https://web.archive.org/web/20180523163917/https://www.facebook.com/scabiesresearchproject/) was shared to ten relevant facebook.com hosted groups (online supplementary table 2) and messages sent to members whose posts indicated they fitted the inclusion criteria.

Data collection, processing, and analysis

Single audio-recorded, semi-structured telephone interviews were conducted from a private room in the Department of Primary Care and Public Health at Brighton and Sussex Medical School by first author NR (who was alone) between November 2017 and February 2018. Participants gave data on personal characteristics and experience, and interviews followed a topic guide (online supplementary file) based on published literature and guidance from experts in scabies (epidemiology, JAC;

For peer review only - http://bmjopen.bmj.com/site/about/guidelines.xhtml

dermatology, SLW; medical acarology, JM). They were scheduled for c.40mins, but so as to provide sufficient information power[25] participants were told they could speak longer as necessary, and to not feel precluded by the topic guide from relating any experiences and perspectives they thought relevant to the study aims. NR conducted data collection and thematic analysis simultaneously to determine when data saturation was reached,[26,27] which was prospectively defined as being no new themes emerging in three consecutive interviews. Once data saturation was observed no new interviews were conducted as the team could 'be reasonably assured that further data collection would yield similar results and serve to confirm emerging themes and conclusions.'[28] Field notes were not made during interviews, instead verbatim transcription was undertaken by professional service (9 of 12 interviews), or by NR (3). NR then coded text in NVivo 11 (QSR International, Melbourne), generated an initial framework matrix and exported it to Microsoft Excel, in which qualitative framework analysis was completed (as outlined by Gale *et al.*[29]). Groups of meaningful concepts were sought in the data and arranged hierarchically into main themes and subthemes. Each transcript was studied again twice for more evidence of the concepts. Subsequently author JM reviewed the reported themes and subthemes and read the transcripts, finding no new relevant themes or subthemes than those grouped by NR. Participants were not asked to subsequently comment on their transcripts or the general findings.

Patient and public involvement

Patients and the public were not involved in research design. Members of healthcare staff networks aided recruitment. CS is an aid worker with scabies outbreak experience in the study setting and co-authored the paper (they were not an interviewee).

RESULTS

Participants

All potential participants who responded to our advertising agreed to be interviewed after initial discussion of the study. Recruitment halted after 12 interviews as data saturation was assessed to have been reached (no new themes emerged in the last three interviews). Interviews lasted 34–71min (mean 47). The twelve interviewees consisted of four doctors, four nurses, three allied health workers (AHWs: an ECG technician, podiatrist, and a first aider), and one medical student. Ten were NGO volunteers, one an independent volunteer, one was employed by a public health service. Six had worked in informal camps, nine in formal ones, across camps at 15 locations within seven European countries: Greece, France, the Netherlands, Serbia, Belgium, Turkey, Macedonia. Geographic distribution of the camps (figure 2) reflected eastern entry migration routes into Europe, and clustering at the English Channel by those seeking to enter the UK. Experience in camps ranged from 4 d to 2.5 y (median 84 d, IQR 233–251 d) (participant characteristics, online supplementary table 3).

For peer review only - http://bmjopen.bmj.com/site/about/guidelines.xhtml

Diagnosis, treatment, and outbreak management

Scabies diagnosis, treatment and outbreak management are summarised in table 1. Eleven interviewees (92%) diagnosed scabies based on history and clinical findings. Two AHWs and the medical student confirmed diagnoses with a doctor. One participant occasionally used skin-scrapings in a Greek formal camp and during an outbreak in Serbia. In one camp diagnosis was routinely given by a dermatologist and confirmed by microscopic examination. No one reported dermatoscopy. Three organisations attempted to follow Médecins Sans Frontiers' (MSF) guidelines for ordinary scabies treatment[30] (online supplementary file), one combined this with British National Formulary advice.[31] Another had their own scabies protocol.

Only seven participants (58%) reported scabicides were provided in camps they worked in whilst they were there. The remaining five (42%) stated only symptomatic management was offered (detailed in table 1) because it was felt proper treatment would be futile due to high re-infestation risks (four participants), or due to prescribing restrictions (one participant). A third of participants said treatments varied, depending on availability. When NGOs offered scabicides, they were generally given to anyone complaining of itch, due to a low threshold of suspicion (5 of 7). However, one NGO only treated individuals with a dermatologist-confirmed diagnosis, another only treated 'serious cases'.

Eleven participants (92%) believed scabies was difficult to manage, whereas a GP following a formal camp's scabies protocol considered it "just a nuisance" (P7). Interviewees described high variation in outbreak management (table 1), particularly in timings used for permethrin application. These ranged from singular treatment to second applications up to a week later. Three participants (25%) could not recall timings or if close contacts were treated. In four interviews, participants claimed to treat all close contacts regardless of symptoms. Only three participants (25%) reported use of oral ivermectin for scabies, which was limited due to lack of availability. Five interviewees (42%) reported environmental decontamination was considered too challenging to conduct. Cleaning/replacing clothing and bedding was the most common form of decontamination. Individuals were given replacement clothing/bedding and old items washed (2 of 7), disposed of (3 of 7), or put into plastic bags for 72 h (2 of 7). One formal camp in Greece burned blankets. One organisation built an inventory of rental clothes for individuals while theirs were in bags. Generally, decontamination of furniture and shelters was not attempted (only mentioned in two interviews). Where it was, it involved disinfectant or covering furniture and insides of shelters in plastic sheeting for 72 h.

For peer review only - http://bmjopen.bmj.com/site/about/guidelines.xhtml

Table 1. Diagnosis, treatment, and outbreak management of scabies (n=12 participants)

**Diagnosis**		
**Clinical features observed in patients**	Itching, mainly at night	12
	Rash	7
	Burrow lines	6
	Infected lesions	2
**Parts of the body examined**	Hands	8
	Arms	7
	Torso	6
	Genitals	4
	Legs	3
	Feet	3
	Armpits	2
**Equipment used during examination**	Gloves	7
	Nothing	2
	Lights	2
	Microscope	2
	Dermatoscope	0
**Duration of examination**	<5 minutes	8
	5–10 minutes	2
	>10 minutes	1
**Treatment**		
**Treatment guidelines**	None	8
	MSF clinical guidance	3
	British National Formulary	1
	NGO's own protocol	1
**Management of individual**	Symptomatic management only	5
	Antihistamines	3
	Topical steroid	1
	Moisturizer	1
	Antibiotics for infections	1
	Varying treatment depending on availability	4
	Unknown topical treatment	3
	1st line: permethrin 5% cream. 2nd line: benzyl benzoate. Symptomatic treatment: antihistamines.	2
	1st line: ivermectin PO. 2nd line (or if ≤15kg): permethrin 5% cream	2
	Permethrin 5% cream only	1
**Who was given acaricide treatment**	No one	5
	Anyone with itching, plus close contacts	4
	Anyone with itching	1
	Only "serious cases"	1
	Cases confirmed by dermatologist, plus close contacts	1
**Outbreak management**		
**Timing of mass treatments**	No treatment	4
	Unknown	3
	Cases treated twice, one week apart (permethrin)	1
	Cases & symptomatic contacts treated x2 a few days apart	1
	(permethrin), asymptomatic contacts x1	1
	Cases treated x1, 3 days apart (permethrin)	1
	Cases treated x2, 24 hours apart (permethrin and benzyl benzoate)	1
	Case treated x1 (permethrin)	1
	Cases treated x1 (ivermectin)	
**Treatment of close contacts**	No treatment available to anyone	4
	All close contacts	4
	Unknown	3
	Symptomatic contacts	1
**Environmental decontamination**	Clothing	7
	Bedding	7
	None	5
	Furniture	1
	Shelters	2

For peer review only - http://bmjopen.bmj.com/site/about/guidelines.xhtml

Camp characteristics and perceived barriers and facilitators to effective scabies outbreak management

Camp Characteristics

All participants described camps as difficult places to work; legal status seemed unconnected to environmental quality. Participants described poor living standards (quotes, online supplementary file, p.9–11). For example, P8 described a formal camp (Greece):

> "It's horrible. There's only one water source for over two thousand people, there's not enough clothes, people are sleeping outside, there's not enough tents, the hygiene is terrible. I was there in the summer so it was hot, there's not enough shading and people couldn't find a place outside the sun. It's more horrible now, as its winter, so it's freezing, and there's no heating. To be honest it's worse than the refugee camps I've seen in Africa."

P9, who had worked in formal and informal camps (France and Greece) spoke similarly:

> "They are a human rights violation. I don't think people should be living in them, especially in a first world European country this should not be happening. People are living in cold tents without heating and electricity, there's rats, there's scabies, there's a lack of toileting facilities, a lack of hygiene, lack of food."

Ten participants (83%) believed shelter was inadequate or non-existent, two (17%) very basic. Twelve (100%) described lack of suitable Water, Sanitation, and Hygiene (WaSH) facilities. Five (42%) described safety worries regarding: fights between residents; police mistreatment; instability of camp structures.

Barriers and facilitators to effective scabies outbreak management

Themes and subthemes are illustrated by example quotes in tables 2 (barriers) and 3 (facilitators), with 16 pages of themed quotes in online supplementary file. Each interviewee described many barriers to effective scabies management; four key themes arose.

Lack of WaSH facilities: Lack of access to washing facilities for fabrics to prevent re-infection was identified by 10 interviewees (83%). Three interviewees (25%) believed sharing belongings was promoting re-infestation, amplified by inability to wash them between users. Six interviewees (50%) described difficulties associated with camp residents inability to wash themselves, due to non-existent or insufficient showers. This was especially relevant regarding topical scabicides, which should be washed off.[4]

For peer review only - http://bmjopen.bmj.com/site/about/guidelines.xhtml

Table 2. Barriers to providing effective scabies management.
Further themed participant quotes in online supplementary file, p.11–21.

**Key Themes** *Subthemes*	**Comments from participants**
**Lack of WaSH Facilities**	
Inability to wash belongings	"Part of the treatment is also to wash the bedding, and all clothing in hot water, dry it in a drier... those facilities weren't readily available for anybody at the camp... even if we'd had a sort of endless supply of Permethrin or Ivermectin, I think it still would have been problematic with this problem of re-infection." (P6)
Lack of showering facilities	"There is really no shower and there are some areas that are a kind of shower area, but it's not really a shower and its cold water." (P5)<br><br>"Treating scabies was problematic because… the lotion, you're supposed to keep it on for 12 hours and then wash it off… and access to showers was not always that easy." (P6)
Sharing of belongings	"Charities came in to give clothes, but the clothes weren't ever washed and when someone went across the border... their stuff was left, and whoever was in the tent just used that." (P1)
**Social**	
Language barriers	"They don't understand why they have to use the cream and what's going on. So, they feel reluctant to comply, and they're like no, it doesn't work. If you don't have proper translators to translate anything to you that also could be a big barrier." (P2)
Stigma	"Didn't want to tell the other people they had scabies... there is some sort of stigma of course if you have some sort of rash that you are not clean or something." (P8)
Non-compliance to treatment	"I would say to put the stuff all over your body, head down to the toes... to do it all in one night and keep it on for so many hours and then they walk away and then only put a bit of cream here, and a bit of cream there, for a week and then they come back and it hasn't got better." (P7)
Transient population	"You have to trace who might be the contact... in the refugee setting you have to also to treat patients that might be from the same family, and they just move around. You can't really tell who has slept here, who has not slept here, who might possibly get it." (P2)
**Healthcare**	
Availability of medication	"We don't have the treatment for it, because we don't have the money to buy in the treatment." (P4)<br><br>"The medication available is the least convenient medication you could use. If you could use a pill for everyone that would be so much easier." (P10)
Lack of privacy for medical examinations	"Everything sees what we are doing, so if someone has to show something we don't have a closed examination room... you can't really examine them... you can't really touch and see in your own eyes... you have to rely on the photos because they're too shy to show it." (P2)
Inexperienced staff	"European doctors did not have a lot of experience with scabies, so I think a lot of them didn't realize what they were seeing." (P10)
**Organisational**	
Crowded living conditions	"People were living in fairly cramped conditions which obviously made transmission a high possibility." (P6)
Poor coordination between organisations	"Lack of coordination and appropriate communication... I don't think it's the resources; the resources are there they're just not put together in the right way." (P10)<br><br>"Logistical problems, sufficient manpower, sufficient washing machines, sufficient bedding, who cares for this?" (P11)
Poor treatment by authorities	"refugees have their bedding and their coats and shoes confiscated by police at night." (P3)<br><br>"People were hiding from the authorities... the settlement or their place where they would sleep would not be there because tents were being like routinely slashed and belongings were being destroyed by the police and authorities." (P12)

Social barriers*:* Eight interviewees (67%) felt language differences obstructed understanding individuals' problems and explaining treatment and decontamination. Four (33%) felt stigma associated with scabies adversely affected management, as it reduced presentation to services and individuals were less likely to inform close contacts who required treatment. Treatment non-compliance was considered a barrier in four interviews (33%), caused by complicated management

For peer review only - http://bmjopen.bmj.com/site/about/guidelines.xhtml

regimes, and residents' competing priorities. Population mobility concerned six interviewees (50%), who felt it affected contact tracing, follow-up treatment, and disrupted elimination efforts.

Organisational barriers: Six participants (50%) perceived overcrowding as a major barrier to effective management. Large dormitory-style rooms were said to be the hardest in which to determine close contacts, as were bigger units in which blanket partitions were used between families. Five interviewees (42%) described poor coordination between organisations (both NGOs and governmental organisations) as a barrier. This primarily involved organisations providing different resources not communicating about appropriate timings (e.g., lack of connection between treatments, clothing replacement, access to showers). Two participants described disrespectful care and stigmatisation by healthcare workers. Ten participants (83%) described how lack of support and mistreatment by state authorities acted as a major barrier, most of which was described in informal camp settings (7 of 10). Two interviewees mentioned residents facing police brutality, including having tents slashed, and clothing, shoes, and bedding confiscated. This encouraged sharing (with potential implications for transmission), and by adding further challenges to everyday life it reduced volunteer and patient capacity to focus on treatment and decontamination. One participant reported authorities "sometimes they cut… water, they try not to get us to be able to give out clothes, they raided our distribution place." (P2) Their explanation was: "What the government is trying to do is, they're trying not to get the refugees too comfortable."

Healthcare barriers: Eight interviewees (67%) considered scabicide availability a major barrier. Cost, prescribing restrictions, and variations in pharmacy stocking contributed; for most NGOs availability relied on donations. Inconsistency complicated treatment and fostered mistrust. Three interviewees (25%) felt lack of privacy impacted care. Some residents attempted to circumvent this by providing photos on phones, but this was not considered as useful as examinations. Five interviewees (42%) felt healthcare workers accustomed to European work were unfamiliar with scabies, which was exacerbated by volunteer-turnover.

Facilitators: Two participants (17%) said they did not experience factors aiding scabies treatment and management, the remaining described five, when available: WaSH facilities; language facilitators; education; treatment incentives; coordination of organisations (table 3). Three (25%) felt access to showers and washing machines was vital, as clothing and bedding could be reused without concern of reinfection, and topical treatment guidelines adhered to. Nine interviewees (75%) described the importance of translators in overcoming language barriers. Only three NGOs used official translators, the rest were aided by residents. One participant described using recorded voice messages and information sheets in different languages. Four (33%) believed resident education aided outbreak management, by alerting them to others who needed treatment and by providing education on correct treatment application. This was also thought to have reduced stigma within camps. Four (33%) stated

For peer review only - http://bmjopen.bmj.com/site/about/guidelines.xhtml

incentives (e.g., new clothing or just underwear) aided treatment compliance. Eight interviewees (67%) reported positive impacts from inter-organisational communication, including information and resource sharing, and enabling referrals. The GP who described scabies as "just a nuisance" (P7), attributed that to having a protocol followed by all organisations. In particular, the "ability to pass on a systematic and well-rehearsed message" (P7) was thought to have improved adherence.

Table 3. Facilitators to providing effective scabies management.

Further themed participant quotes in online supplementary file, p.21–24.

**Key Themes** *Subthemes*	**Comments from participants**
**WaSH Facilities**	"People have access to showers... clothes had to be washed in the washing machines… everything else was taken out and burnt. That was the protocol... we didn't have outbreaks." (P7)
**Social** *Language facilitators*	"We had our own translator who spoke several languages, and there were people who lived in the camp, working or volunteering as translators." (P1)
Education	"If you educate the patient very well, make them understand very well what's going on, and what needs to be done... They understand, and they start to even manage to recognise their friends." (P2)
**Treatment incentive**	"When they found out we would give them another set of clothes and we would buy underwear for them, some more people were interested as they had an incentive to take part." (P8)
**Coordination of organisations**	"We give them a cream 24 hours before and then asked them to get showers, and then we put the creams again. So we partner with shower teams, and also, we partner with people from the distribution team so we could get them new fresh clothes." (P2)

DISCUSSION

Principal findings

Participants reported in the camps they had worked scabies diagnosis was primarily clinical (without dermatoscopy), and treatment and outbreak management varied highly. Nearly half said scabicides were unavailable; only symptomatic management was offered. They described camps as difficult places to work, with poor living standards for residents. Key perceived barriers to control were (i) lack of WaSH facilities, specifically: absent/limited showers (difficult to wash off topical scabicides); inability to wash clothes and bedding (may have increased transmission/re-infestation), (ii) social factors: language; stigma; treatment non-compliance; mobility (interfering with contact tracing and follow-up treatments), (iii) healthcare factors: scabicide shortages and diversity; lack of examination privacy; staff inexperience, (iv) organisational factors: overcrowding; ineffective inter-organisational coordination; lack of support and maltreatment by state authorities (e.g. not providing basic facilities; obstruction of self-care by camp residents and NGO aid).

Strengths and weaknesses

This qualitative study succeeded in giving voice to the subjective perspectives and experiences of healthcare staff involved in this previously unstudied topic. However, recruitment methods may have prevented fully representative participant selection. Firstly, because advertising, recruitment, and

For peer review only - http://bmjopen.bmj.com/site/about/guidelines.xhtml

interviews were conducted solely in English, which excluded the experiences of non-English speaking individuals. Secondly, as we assessed data saturation had been reached and halted recruitment, there was a fairly small number of participants. However, whilst twelve participants might be small for a quantitative study, our sample size does correspond with what Guest et al.[26] determined was usually sufficient to reach data saturation in qualitative studies focused on narrow topics. Seeking to 'make evidence-based recommendations regarding nonprobabilistic sample sizes for interviews'[26] they found saturation not only usually occurred in the first twelve interviews, but that key constituents of meta-themes presented as early as in the first six. One study strength was that the relatively long semi-structured interviews gave participants opportunity to articulate their perspectives relatively unconstrained by researcher presumptions over what may have been important to them about their experiences. The resultant transcripts provided a large amount of rich data on the perspectives and experiences of the participants (for example see extensive quotes in online supplementary file, p. 9–24), and thus had far more 'information power' [25] than could be expected from a short survey with higher participant numbers. Telephone interviews prevented observation of non-verbal cues, but did enable international recruitment in a timely and resource-efficient manner. However, the retrospective nature of the study may have introduced recall bias. Future work in refugee/migrant camps could reduce this limitation by interviewing healthcare staff during outbreaks, either remotely or in-person. Our study aimed only to describe the perspectives and experiences of the participants, seeking to understand the subjective meaning of the phenomena to them. Given these aims, as our study draws on the understandings of the research subjects themselves its method is high on internal validity, but the findings are not necessarily generalizable. We take the latter limitation as a given, but not one that invalidates the study. However, inference and public health recommendations based on this research must be pragmatic with limitations acknowledged.

Louka et al.[22] report scabies epidemiology in refugee/migrant populations in Greece, while more general quantitative studies from across Europe touch on scabies in these populations (for example, see [19-21]). However, to our knowledge our study is the only qualitative work published on healthcare staff perspectives on barriers and facilitators to treating and managing scabies within refugee/migrant camps, either in Europe or globally. This underlines the major strength of our study in providing a platform for these underrepresented healthcare voices, yet given this it is difficult to compare our findings to others. Wollina et al.[32] do usefully recount their related experience in a German hospital which received dermatological referrals (scabies being the most common) from a nearby formal refugee camp. Even in this more resourced setting language barriers were an issue, as they were for some of our participants. An environmental health assessment of a French informal camp with a high scabies incidence detailed a very similar environment to those described by many of our participants: 'Shelters were vastly inadequate and directly exacerbating the ill-health and

For peer review only - http://bmjopen.bmj.com/site/about/guidelines.xhtml

psychological distress suffered by some residents of the camp... Residents at all sites reported difficulties with washing themselves, their clothes and their bedding.'[18]

The wider context of scabies across Europe

Some studies suggest scabies infestations may have increased across Europe recently, including research conducted in Turkey, France and the Netherlands, countries in which a number of our study participants were based.[33–38] In the general population of the Netherlands recorded scabies diagnosis per 1,000 persons per year increased fourfold 2011–2020 (0.6 to 2.6), in parallel with a sixfold increase in scabicide dispensing over the same period. Most dispensations were permethrin; however large nosocomial outbreaks were linked to a peak of ivermectin prescriptions in 2014–2015.[34] Similarly, German studies found increasing numbers of cases since 2009, most notably an elevenfold increase in persons 15–24 years of age from 2009–2018.[35, 36, 38] In Croatia, where reporting of scabies is mandatory, general incidence increased sixfold 2007–2017, and multiple outbreaks were reported from adult care and nursing homes.[37] Some data suggests an association between population movement and higher incidence in the general population. However, though refugee/migrant groups have been disproportionately affected by scabies when living in the crowded camps which are the subject of our study, they are unlikely to be the cause of changes in reported overall prevalence within the wider population.[33, 35–38] Suggested explanations given for this claimed trend include increased number of sexual contacts, treatment failure in younger patients with presumed poorer compliance, and aging populations.[33, 34, 36–40] The observed trend may also be in part a result of reporting bias, given the higher attention scabies is now being given in some countries following its adoption as a WHO NTD.

Implications for clinicians and policymakers

Diagnosis and treatment

It is unsurprising no participants reported dermatoscopy use given equipment expense, paucity of trained individuals, and its reduced sensitivity when outbreak size limits examination time.[41] However, subsequently published (2018) consensus criteria for scabies diagnosis developed by IACS (https://www.controlscabies.org) provides a standard for clinical diagnosis. This is summarised in Box 1, and fully detailed in Engelman et al.,[42] though it requires validating in refugee/migrant camp settings. Some participants suggested screening on entry to camps to reduce ingress of new infestations. This was also suggested by Wollina et al.[32], has been carried out in reception centres[43], and may be feasible in camps. However, in informal camps screening may not be consented to by those seeking entry and NGOs may have insufficient resources to adequately screen, potentially leading to false positives/negatives. The asymptomatic incubation period would also mean some potential outbreak index cases would still be missed.

For peer review only - http://bmjopen.bmj.com/site/about/guidelines.xhtml

Participants reported problems using topical scabicides. Firstly, topical treatment involves full body coverage with creams, left on for at least half a day. Privacy was limited and individuals living in close proximity may have wished to avoid being seen treating themselves for a stigmatised condition, thus discouraging appropriate application. Secondly, topical scabicides need to be washed off to reduce potential irritancy[4] but facilities were usually only minimally available (if at all). Oral ivermectin Mass Drug Administrations (MDAs) are easier to administer, and were conducted successfully in Dutch and German reception centres during the period.[43, 44] However, ivermectin is expensive in Europe and unlicensed for scabies in most European countries[12] despite a good safety profile,[45,46] and inclusion in WHO's essential medicines list.[47] The potential health impacts of chronic conditions secondary to scabies[7–9] supports the value of timely MDAs in these settings. Participants reported widespread use of camp residents as informal translators, but this raises confidentiality concerns, and ad-hoc interpreters often misinterpret/omit important medical information.[48]

Outbreak management and camp environments

Discrepancies in management reflect lack of widely accepted standard outbreak guidelines. Guidance (for example,[30, 31]) is often for individual households and does not make adaptions based on the logistical difficulties of camp environments. Institutional scabies outbreaks guidelines exist, yet none are based on a systematic review of the published evidence.[12] This is also the case for the 'European Guideline'.[49] More broadly there is a lack of research regarding the most effective interventions for preventing scabies transmission to close contacts,[50] particularly in refugee/migrant settings. Organisations often favoured attempting to decontaminate clothing and bedding rather than shelters, yet living mites have been found in dust samples on floors and furniture.[51] Variance in reported decontamination reflects a sparse historic evidence-base.[12] However, recent experimental work[52] could support uniform action. Where washing/drying machines are available (few camps in this study) ≥10min at ≥50°C will kill all *S.scabiei* mites and eggs.[52] Lacking such resources, some participants reported isolating potential fomites with plastic bags/sheeting for 72 h. Due to mite desiccation this may have been effective in temperate-dry settings, but if bags were left outdoors in the cold and damp mite survival can be longer. At the outer limit, in warm-humid conditions elimination can take >8 d.[52] Existing guidance should be updated. Even though improving WaSH facilities is unlikely to reduce skin-to-skin transmission,[3] it nevertheless would support treatment and control where transmission via fomites may be a risk, when topical scabicides are used, and to minimise secondary infections.[14] It could also be expected to reduce transmission of other diseases. It should however be noted that (primarily) ivermectin-based MDAs have achieved very good results in community intervention trials across Oceania (for a summary, see [53]) without any environmental measures on hygiene or disinfection of bedding etc. (for example, see work in the Solomon Islands [54]). Participants described crowded and substandard living conditions that likely provided an ideal

For peer review only - http://bmjopen.bmj.com/site/about/guidelines.xhtml

environment for scabies transmission, whilst obstructing treatment and outbreak management. Some believed lack of political will was preventing provision of suitable living conditions and access to healthcare, as governments feared they would encourage people to stay too long. Going further, participants reported actual obstruction by authorities of resident self-care and NGO aid efforts, with implications for transmission and control of scabies.

The meaning of common scabies occurrence in camps in Europe

Scabies prevalence differs between world regions, but its occurrence is near ubiquitous in human populations worldwide.[4,13] It follows from this premise that ingress of *S.scabiei* into at least some of the camps was practically inevitable. However, the observed regularity and size of outbreaks wasn't. We aimed to provide insights into experiences and perspectives of healthcare staff who treated scabies or managed associated outbreaks in refugee/migrant camps in Europe 2014–17, but we did not set out to specifically determine the meaning, the explanation, underlying why they took place. To our knowledge the only published research with an objective close to that is Louka et al.[22], who aimed to 'investigate changes over time [in scabies epidemiology in health care centres for refugees and asylum seekers in Greece] and factors relating to these changes'. They note that 'outbreaks of scabies cases coincided with peaks in other infectious diseases' and suggest a possible explanation may be 'an increase of the refugees/asylum seekers residing in the centers at the specific time points, [and] the crowded conditions within the centers'.[22] This suggestion is in line with prior studies which demonstrated that high density of potential hosts is a major transmission driver of scabies.[14] For a detailed overview of scabies transmission drivers in other semi-closed institutional settings where outbreaks are a regular occurrence (e.g., residential settings for elderly people, children and those with learning disabilities; prisons; schools; hospitals and hostels) see Middleton et al.[14] Many of these drivers were mentioned by our participants in some form whilst discussing their experiences of diagnosis, treatment, outbreak management, and camp environments. Specifically of those discussed in [14] the following were mentioned by our studies participants: high densities of potential hosts; residents moving between semi-closed units; communication difficulties; reduced access to appropriate treatment; diagnostic error and/or delay; reduced access to laundry. It is beyond the capacity of this study to determine the relative contribution of these factors to outbreak regularity and size, and indeed their contribution is likely to have been different in different camps, and at different times. Clinicians and policymakers tasked with managing scabies in similar refugee settings may find it helpful to carry out site-specific assessment of scabies transmission drivers to guide intervention and treatment, using the guidance on how to do so provided by Middleton et al.[14]

More broadly, our view is that without support and coordination of all those responsible for care of refugees/migrants, successful scabies management will remain challenging, and the resource poor and overcrowded aspects of camp environments which enable outbreaks will remain unchanged. Indeed,

For peer review only - http://bmjopen.bmj.com/site/about/guidelines.xhtml

in late 2020 a similar situation to those described in 2014–2017 was observed by co-author CS in a formal camp on Lesvos (Greece). The NGO CADUS, in which CS works, had been deployed within a WHO initiative to provide medical care. Scabies was widespread among the camp population of 7500, but the eradication program had to begin with a capacity of ten treatments per day. A small volunteer-staffed NGO provided treatment, including provision of (camp-external) showers, clothes and blankets exchange. The overcrowded camp lacked adequate facilities to wash clothes, and mostly only symptomatic treatment could be offered, which CADUS medical staff viewed as highly unsatisfactory.

Future Recommendations

Our study demonstrates the importance of having clear protocols for scabies diagnosis, treatment, and management in refugee/migrant camps. Systematic evidence evaluation must be combined with input from individuals and organisations on-the-ground to provide high-quality feasible guidelines that explicitly consider camp settings, formal and informal. Given most participants were not doctors, guidelines need to be understandable to everyone involved. Though topicals should be provided for children and pregnant women, difficulties associated with them in camps could be avoided for most adult patients through wider licensing and availability of oral ivermectin. The 'WHO Informal Consultation on a Framework for Scabies Control' recommended populations with ≥10% prevalence should receive ivermectin MDAs;[55, 56] we suggest this should include refugee/migrant camps. Strategies for lower levels of endemicity in camps should also be investigated. More research and advocacy for those living in refugee/migrant camps in Europe could help improve management of common health problems. This is particularly relevant for managing scabies in camps, where substandard conditions, inadequate resources, and lack of guidance can lead to poor quality of care and ineffective treatment. As our participants indicate, much of this work is shouldered by small, volunteer staffed NGOs operating with minimal resources in highly challenging (often illegally occupied) settings. We in the wider healthcare community should reflect on how we can better support these initiatives, and those they serve.

For peer review only - http://bmjopen.bmj.com/site/about/guidelines.xhtml

Box 1. Summary of 2018 IACS criteria for the diagnosis of scabies

A: Confirmed scabies

At least one of:
A1: Mites, eggs or feces on light microscopy of skin samples
A2: Mites, eggs or feces visualized on individual using high-powered imaging device
A3: Mite visualized on individual using dermoscopy

B: Clinical scabies

At least one of:
B1: Scabies burrows
B2: Typical lesions affecting male genitalia
B3: Typical lesions in a typical distribution and two history features

C: Suspected scabies

One of:
C1: Typical lesions in a typical distribution and one history feature
C2: Atypical lesions or atypical distribution and two history features

History features

H1: Itch
H2: Close contact with an individual who has itch or typical lesions in a typical distribution

Notes

1. These criteria should be used in conjunction with the full explanatory notes and definitions (in preparation).
2. Diagnosis can be made at one of the three levels (A, B or C).
3. A diagnosis of Clinical and Suspected scabies should only be made if other differential diagnoses are considered less likely than scabies.

Attribution: Reprinted from Engelman et al (2018),[42], which is licenced under https://creativecommons.org/licenses/by/4.0). No changes have been made.

463

For peer review only - http://bmjopen.bmj.com/site/about/guidelines.xhtml

STATEMENTS

Online supplementary file: This web only file has been produced by the BMJ Publishing Group from an electronic file supplied by the authors and has not been edited for content.

Contributors: This study was carried out as part of the work of the Scabies Research Team based at Brighton and Sussex Medical School, London School of Hygiene and Tropical Medicine, and University of Southampton. Authorship order is alphabetical by surname, except the first (primary researcher) and last (lead supervisor). For clarity we detail contributions using the CRediT Contributor Taxonomy (https://credit.niso.org/), and provide employment and disciplinary descriptions. Conceptualisation: JM, JAC. Formal analysis: NR, JM. Investigation: NR, JM. Methodology: JM. Project administration: NR, JM. Supervision: SL, JM. Writing – original draft: NR, JM. Visualization: NR, JM. Writing – review & editing: NR, JAC, MGH, SL, CS, SLW, JM. NR is a global emergency medicine clinical fellow; JAC a public health physician and epidemiologist; MGH a senior research fellow [global health]; SL a research co-ordinator; CS is an aid worker; SLW a consultant dermatologist, and associate professor [infectious and tropical diseases]; JM a research fellow [public health; neglected tropical skin diseases].

Ethics: This study involves human participants and was approved by Brighton and Sussex Medical School Research Governance and Ethics Committee (ER/BSMS3398/1).

Acknowledgments: We thank the interviewees for their time and openness with our study, and the administrators and members of the healthcare staff networks who advertised our study on-line. We are also grateful to our colleagues at Brighton and Sussex Medical School, Elizabeth Ford (for input into study design), Jessica Stockdale (for producing figure 2), and Maya Khan (for setting up our Facebook recruitment pages).

Data availability: Online supplementary file includes 16 pages of themed quotes from interviews. Other data are available on reasonable request from the corresponding author.

Funding: This research was financially supported by Brighton and Sussex Medical School from internal funds (N/A). JM is funded by the National Institute for Health and Care Research (NIHR) Applied Research Collaboration Kent, Surrey, Sussex (NIHR200179) and the NIHR Global Health Research Unit on Neglected Tropical Diseases (NIHR131996). The views expressed are those of the authors and not necessarily those of the NHS, the NIHR or the Department of Health and Social Care.

Competing interests: None declared.

For peer review only

For peer review only - http://bmjopen.bmj.com/site/about/guidelines.xhtml

REFERENCES

1. Trettin B, Lassen JA, Andersen F, et al. The journey of having scabies– a qualitative study. J Nurs Educ 2019;9:2. doi: 10.5430/jnep.v9n2p1
2. Mellanby K. Scabies in 1976. J R Soc Promo Health 1977;97:32–36. doi: 10.1177/146642407709700112
3. Middleton J, Cassell JA, Jones CI, et al. Scabies control: the forgotten role of personal hygiene- Authors reply. Lancet Infect Dis 2018;18:1068-1069. doi: 10.1016/S1473-3099(18)30558-9
4. Griffiths C, et al. Rook's Textbook of Dermatology. New York: Wiley-Blackwell 2016:1111–1119.
5. Swe PM, Reynolds SL, Fischer K. Parasitic scabies mites and associated bacteria joining forces against host complement defence. Parasite Immunol 2014;36:585–593. doi: 10.1111/pim.12133
6. Romani L, Steer AC, Whitfeld MJ, et al. Prevalence of scabies and impetigo worldwide: a systematic review. Lancet Infect Dis 2015;15:960–967. doi: 10.1016/S1473-3099(15)00132-2
7. Chung SD, Wang KH, Huang CC, et al. Scabies increased the risk of chronic kidney disease: a 5-year follow-up study. J Eur Acad Dermatol 2014;28:286–292. doi: 10.1111/jdv.12099
8. Thornley S, Marshall R, Jarrett P, et al. Scabies is strongly associated with acute rheumatic fever in a cohort study of Auckland children. J Paediatr Child H 2018;54:625–632. doi: 10.1111/jpc.13851
9. Thornley S, King R, Marshall R, et al. How strong is the relationship between scabies and acute rheumatic fever? An analysis of neighbourhood factors. J Paediatr Child H 2020;56:600–606. doi: 10.1111/jpc.14697
10. Cassell JA, Middleton J, Nalabanda A, et al. Scabies outbreaks in ten care homes for the elderly: a prospective study of clinical features, epidemiology, and treatment outcomes. Lancet Infect Dis 2018;18:894–902. doi: 10.1016/S1473-3099(18)30347-5
11. NICE. Clinical Knowledge Summaries: Scabies [online]. 2017. https://cks.nice.org.uk/scabies#!scenario (accessed 10 Nov 2017).
12. White LCJ, Lanza S, Middleton J, et al. The management of scabies outbreaks in residential care facilities for the elderly in England: a review of current health protection guidelines. Epidemiol Infect 2016;144;3121–3130. doi: 10.1017/S0950268816001746
13. Engelman D, Cantey PT, Marks M, et al. The public health control of scabies: priorities for research and action. Lancet Infect Dis 2019;394:81–92. doi: 10.1016/S0140-6736(19)31136-5
14. Middleton J, Cassell JA, Walker SL. Scabies management in institutions. In: Chosidow and Fisher eds. Scabies. Vienna: Springer Nature 2023[In Press expected publication October 2023]. ISBN 978-3-031-26069-8
15. UNHCR. Global Trends 2016 [online]. 2017. http://www.unhcr.org/5943e8a34.pdf (accessed 14 July 2018).
16. WHO Europe. Greece: assessing health-system capacity to manage large influxes of migrants [online]. http://www.euro.who.int/__data/assets/pdf_file/0007/300400/Greece-Assessment-Report-en.pdf?ua=1 (accessed 10 Nov 2017).
17. Catchpole M, Coulombier D. Refugee crisis demands European Union-wide surveillance! Eurosurveillance 2015;20. doi: 10.2807/1560-7917.ES.2015.20.45.30063
18. Dhesi S, Isakjee A, Davies T. An Environmental Health Assessment of the New Migrant Camp in Calais [online]. 2015. https://www.birmingham.ac.uk/Documents/college-les/gees/research/calais-report-oct-2015.pdf (accessed 10 Nov 2017).

For peer review only - http://bmjopen.bmj.com/site/about/guidelines.xhtml

19. Kortas AZ, Polenz J, von Hayek J, et al. Screening for infectious diseases among asylum seekers newly arrived in Germany in 2015: A systematic single-centre analysis. Public Health 2017;153:1. doi: 10.1016/j.puhe.2017.07.011
20. Alberer M, Malinowski S, Sanftenberg L, et al. Notifiable infectious diseases in refugees and asylum seekers: Experience from a major reception center in Munich, Germany. Infection 2018:1–9. doi: 10.1007/s15010-018-1134-4
21. Kühne A, Gilsdorf A. [Infectious disease outbreaks in centralized homes for asylum seekers in Germany from 2004–2014.] Ausbrüche von Infektionskrankheiten in Gemeinschaftsunterkünften für Asylsuchende 2004–2014 in Deutschland] Bundesgesundeheitsbla 2016;59:570–577. doi: 10.1007/s00103-016-2332-9
22. Louka C, Logothetis E, Engelman D, et al. Scabies epidemiology in health care centers for refugees and asylum seekers in Greece. PLoS Negl Trop Dis 2022;16: e0010153. doi: 10.1371/journal.pntd.0010153
23. Seebach S, Baleriola E, Valasquez MM, et al. Fear of and anger against the other - the strange, the sick and the imaginary struggle for survival. Digithum: Las humanidades en la era digital 2016;18:13–24. doi: 10.7238/d.v0i18.2811
24. O'Brien BC, Harris IB, Beckman TJ, et al. Standards for reporting qualitative research: a synthesis of recommendations. Acad Med 2014;89:1245-1251. doi: 10.1097/ACM.0000000000000388
25. Malter K, Siersm VD, Guassora AD. Sample Size in Qualitative Interview Studies: Guided by Information Power. Qual Health Res 2016;26:1753–1760. doi: 10.1177/1049732315617444
26. Guest G, Bunce A, Johnson L. How Many Interviews Are Enough?: An Experiment with Data Saturation and Variability. Field Method 2006;18:59–82. doi: https://doi.org/10.1177/1525822X05279903
27. Fusch PI, Ness LR. Are We There Yet? Data Saturation in Qualitative Research. Qual R 2015;20:1408–1416. doi: 10.46743/2160-3715/2015.2281
28. Faulkner SL, Trotter SP. Data Saturation. In: Matthes J, Davis CS, Robert F, et al., eds. International Encyclopaedia of Communication Research Methods. New Jersey: Wiley 2017. doi: 10.1002/9781118901731.iecrm0060
29. Gale NK, Heath G, Cameron E, et al. Using the framework method for the analysis of qualitative data in multi-disciplinary health research. BMC Med. Res. Methodol 2014;13:117. doi: 10.1186/1471-2288-13-117.
30. Médecins Sans Frontiers. Clinical Guidelines: Diagnosis and treatment manual [online]. 2016. http://refbooks.msf.org/msf_docs/en/clinical_guide/cg_en.pdf (accessed 1 April 18).
31. Joint Formulary Committee. British National Formulary [edition number uncollected]. London: BMJ Group and Pharmaceutical Press [year of publication uncollected].
32. Wollina U, Gaber B, Mansour R, et al. Dermatologic challenges of health care for displaced people. Our Dermatol Online 2016;7:136–138. http://www.odermatol.com/odermatology/20162/2.Dermatologic-WollinaU.pdf
33. Bener F. Increase in scabies incidence: a retrospective cohort study. Eu Res J 2021;7:5. doi: 10.18621/eurj.770849
34. van Deursen B, Hooiveld M, Marks S, et al. Increasing incidence of reported scabies infestations in the Netherlands, 2011–2021. PLoS One 2022;17(6):e0268865. doi 10.1371/journal.pone.0268865

For peer review only - http://bmjopen.bmj.com/site/about/guidelines.xhtml

35. Delaš Aždajić M, Bešlić I, Gašić A, et al. Increased Scabies Incidence at the Beginning of the 21st Century: What Do Reports from Europe and the World Show?. Life 2022;12(10):1598. doi: 10.3390/life12101598

36. Reichert F, Schulz M, Mertens E,et al. Reemergence of scabies driven by adolescents and young adults, Germany, 2009–2018. Emerg Infect Dis 2021;27(6):1693. doi: 10.3201/eid2706.203681

37. Lugović-Mihić L, Aždajić MD, Filipović SK, Bukvić I, Prkačin I, Grbić DŠ, Ličina ML. An increasing scabies incidence in Croatia: A call for coordinated action among dermatologists, physicians and epidemiologists. Zdr Varst 2020; 59(4): 264–272. doi: 10.2478/sjph-2020-0033

38. Sunderkötter C, Aebischer A, Neufeld M, et al. Increase of scabies in Germany and development of resistant mites? Evidence and consequences. J Dtsch Dermatol Ges 2019;17(1):15–23. doi: 10.1111/ddg.13706

39. Zhang W, Zhang Y, Luo L, et al. Trends in prevalence and incidence of scabies from 1990 to 2017: findings from the global Burden of disease study 2017. Emerg Microbes Infect 2020;9(1):813-816. doi: 10.1080/22221751.2020.1754136

40. Mbuagbaw L, Sadeghirad B, Morgan RL, et al. Failure of scabies treatment: a systematic review and meta-analysis. Br J Dermatol 2023;ljad308. doi: 10.1093/bjd/ljad308

41. Walker SL, Middleton J, Cassell JA. Scabies outbreaks in care homes for the elderly – Authors' reply. Lancet Infect Dis 2019;19:26–27. doi: 10.1016/S1473-3099(18)30668-6

42. Engelman D, Fuller LC, Steer AC, et al. Consensus criteria for the diagnosis of scabies: A Delphi study of international experts. PLoS Neglect Trop D 2018;12:e0006549. doi: 10.1371/journal.pntd.0006549 .

43. Beeres DT, Ravensbergen SJ, Heidema A, et al. Efficacy of ivermectin mass-drug administration to control scabies in asylum seekers in the Netherlands: A retrospective cohort study between January 2014 – March 2016. PLoS Neglect Trop D 2018;12:e0006401. doi: 10.1371/journal.pntd.0006401.

44. Solbach P, Sdlacek L, Schmidt RE, et al. [Mass therapy with ivermectin for scabies outbreak during the refugee crisis in Germany in 2015] Systemische antiinfektive Therapie eines Skabiesausbruchs mit Ivermectin in einem Flüchtlingslager. Anästh Intensivmed 2017;58:534–541. doi: 10.19224/ai2017.534

45. Marks M, Taotao-Wini B, Satorara L, et al. Long-Term Control of Scabies Fifteen Years after an Intensive Treatment Programme. PLoS Neglect Trop D 2015;9:e0004246. doi: 10.1371/journal.pntd.0004246

46. Kinyanjui T, Middleton J, Guttel S, et al. Scabies in residential care homes: Modelling, inference and interventions for well-connected population sub-units. PLoS Comput Biol 2018;14:e1006046. doi: 10.1371/journal.pcbi.1006046.

47. Middleton J, Walker SL, House T, et al. Ivermectin for the control of scabies outbreaks in the UK. Lancet 2019;10214:2068–2069. doi: 10.1016/S0140-6736(19)32619-4

48. Flores G. The impact of medical interpreter services on the quality of health care: a systematic review. Med Care Res Rev 2005;62:255–299. doi: 10.1177/1077558705275416

49. Salavastru CM, Chosidow O, Boffa MJ, et al. European guideline for the management of scabies. J Eur Acad Dermatol 2017;31:1248–1253. doi: 10.1111/jdv.14351

50. FitzGerald D, Grainger RJ, Reid A. Interventions for preventing the spread of infestation in close contacts of people with scabies. Cochrane DB Syst Rev 2014:CD009943. doi: 10.1002/14651858.CD009943.pub2

51. Arlian LG, Estes SA, Vyszenski-Moher DL. Prevalence of Sarcoptes scabiei in the homes and nursing homes of scabietic patients. J Am Acad Dermatol 1988;19:806–811. doi: 10.1016/s0190-9622(88)70237-6

For peer review only - http://bmjopen.bmj.com/site/about/guidelines.xhtml

52. Bernigaud C, Fernando DD, Lu H, et al. How to eliminate scabies parasites from fomites: A high-throughput ex vivo experimental study. J Am Acad Dermatol 2020;83:241–245. doi: 10.1016/j.jaad.2019.11.069

53. Middleton J. Can ivermectin mass drug administrations to control scabies also reduce skin and soft tissue infections? Hospitalizations and primary care presentations lower after a large-scale trial in Fiji. Lancet Reg Health - West Pac 2022;22: 100454. doi: 10.1016/j.lanwpc.2022.100454

54. Romani L, Marks M, Sokana O, et al. Efficacy of mass drug coadministration of ivermectin and azithromycin for control of scabies and impetigo: a single-arm community intervention trial. Lancet Infect Dis 2019;19:510–518. doi:10.1016/S1473-3099(18)30790-4

55. WHO. WHO informal consultation on a framework for scabies control, meeting report [online]. 2019. https://www.who.int/publications/i/item/9789240008069 (accessed 16 April 2021).

56. Engelman D, Marks M, Steer AC, et al. A framework for scabies control. PLoS Neglect Trop D 2021;15: e0009661. doi: 10.1371/journal.pntd.0009661

FIGURES

Figure 1. Informal and formal refugee/migrant camps.
Left: informal camp in Calais, France, 2015 (VOA News - Nicolas Pinault (2015), public domain, original: https://web.archive.org/web/20210406154625/https://commons.wikimedia.org/wiki/File:Calais2015a.jpg). Right: formal camp Thessaloniki, Greece, 2016 (Konstantinos Stampoulis (2016), used under CC BY-SA 4.0 which also applies to this figure, original: https://web.archive.org/web/20210406155208/https://commons.wikimedia.org/wiki/File:Refugee_hotspot_Fessas_(3).jpg).

Figure 2. Camp locations and number of participants (n=12) with experience at each site. Distribution of camps follows eastern entry migration routes into Europe, and clustering at the English Channel by those seeking to enter the UK. Left to right: Calais, France, 5 participants all active at informal (IF) camps; Dunkirk, France, 3 all IF; Brussels, Belgium, 1 formal (F); Heumensoord, Netherlands, 1 F; Belgrade, Serbia, 1 IF; Macedonia, 1 IF; Patras, Greece, 1 IF; Idomeni, Greece, 1 IF; Thessaloniki, Greece, 3 – 2 F, 2 IF; Athens, Greece, 1 IF; Attica, Greece, 1 F; Lesbos, Greece, 3 –1 F, 2 IF; Çesme, Turkey, 1 IF; Samos, Greece, 2 F; Tilos, Greece, 1 F. (Figure uses adapted basemap by Julio Reis (2006) created using public domain mapping from the European Environment Agency, use and changes made under CC BY-SA 3.0 which also applies to this image. Original basemap: https://web.archive.org/web/20161019140631/https://commons.wikimedia.org/wiki/File:Europe_biogeography_blank.svg

For peer review only - http://bmjopen.bmj.com/site/about/guidelines.xhtml

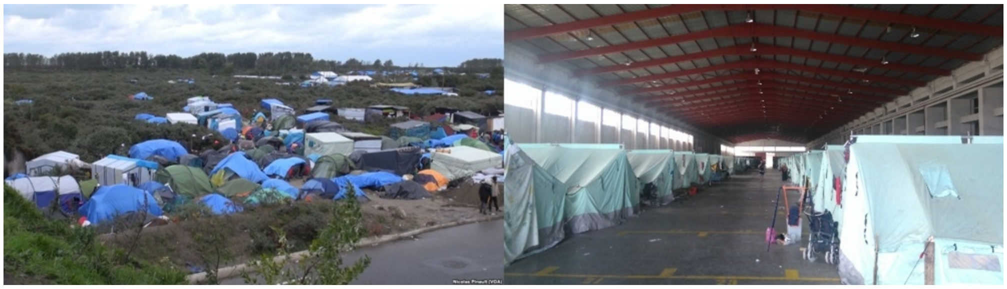

Figure 1. Informal and formal refugee/migrant camps. Left: informal camp in Calais, France, 2015 (VOA News - Nicolas Pinault (2015), public domain, original: https://web.archive.org/web/20210406154625/https://commons.wikimedia.org/wiki/File:Calais2015a.jpg). Right: formal camp Thessaloniki, Greece, 2016 (Konstantinos Stampoulis (2016), used under CC BY-SA 4.0 which also applies to this figure, original: https://web.archive.org/web/20210406155208/https://commons.wikimedia.org/wiki/File:Refugee_hotspot_Fessas_(3).jpg)

263x74mm (96 x 96 DPI)

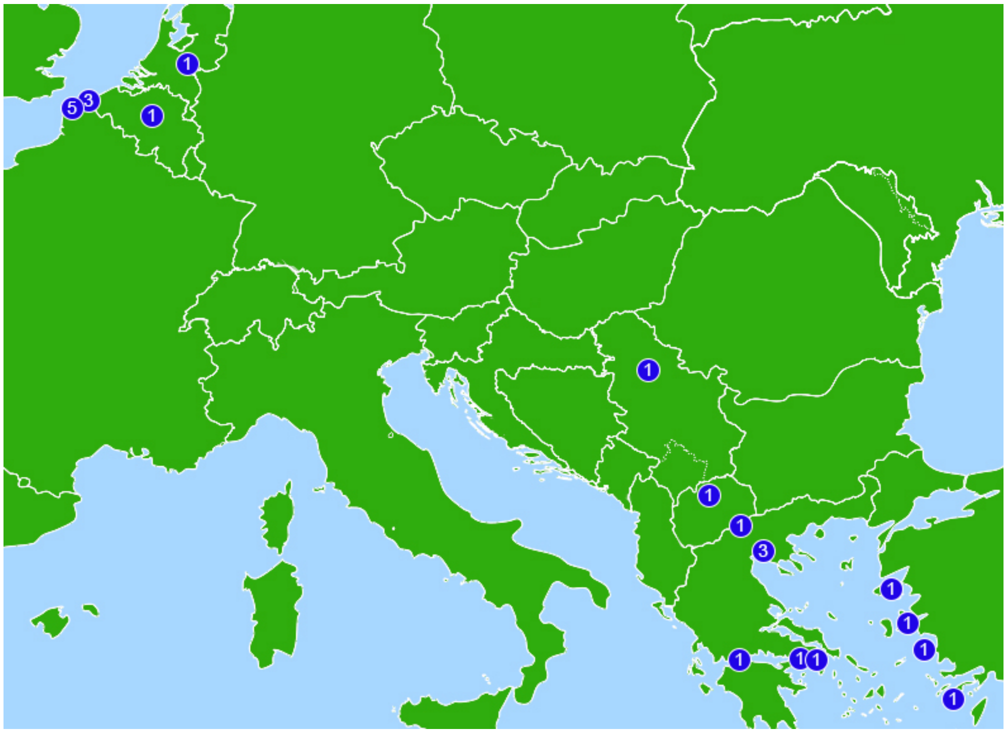

Figure 2. Camp locations and number of participants (n=12) with experience at each site. Distribution of camps follows eastern entry migration routes into Europe, and clustering at the English Channel by those seeking to enter the UK. Left to right: Calais, France, 5 participants all active at informal (IF) camps; Dunkirk, France, 3 all IF; Brussels, Belgium, 1 formal (F); Heumensoord, Netherlands, 1 F; Belgrade, Serbia, 1 IF; Macedonia, 1 IF; Patras, Greece, 1 IF; Idomeni, Greece, 1 IF; Thessaloniki, Greece, 3 – 2 F, 2 IF; Athens, Greece, 1 IF; Attica, Greece, 1 F; Lesbos, Greece, 3 –1 F, 2 IF; Çesme, Turkey, 1 IF; Samos, Greece, 2 F; Tilos, Greece, 1 F. (Figure uses adapted basemap by Julio Reis (2006) created using public domain mapping from the European Environment Agency, use and changes made under CC BY-SA 3.0 which also applies to this image. Original basemap: https://web.archive.org/web/20161019140631/https://commons.wikimedia.org/wiki/File:Europe_biogeography_blank.svg

80x58mm (250 x 250 DPI)

SUPPLEMENTARY FILE

NA Richardson, JA Cassell, MG Head, S Lanza, C Schaefer, SL Walker, J Middleton. Scabies outbreak management in refugee/migrant camps in Europe 2014–17: a retrospective qualitative interview study of healthcare staff experiences and perspectives. Submitted to *BMJ Open* 2023.

CONTENTS

- Examples of news reports of scabies in refugee/migrant camps in Europe 2
- Supplementary Table 1: Standards for Reporting Qualitative Research checklist 3
- Characteristics of researchers 3
- Supplementary Table 2: Facebook.com hosted groups in which the study was advertised 4
- Semi-structured interview topic guide 5
- Supplementary Table 3. Participants' characteristics and settings 7
- MSF clinical guidance for scabies 8
- Themed quotations from interviews 9–24

For peer review only

For peer review only - http://bmjopen.bmj.com/site/about/guidelines.xhtml

Examples of news reports of scabies in refugee/migrant camps in Europe

Cooper C. Calais crisis: Migrants are living in appalling conditions, say doctors: Scabies is spreading like wildfire. The Independent. 26 September 2015, London. [Accessed 10 Nov 2017]. Available from: https://www.independent.co.uk/news/world/europe/calais-crisis-migrants-are-living-in-appalling-conditions-say-doctors-a6668631.html

Lichfield J. French police clear hundreds of asylum-seekers from Calais migrant camps after outbreak of scabies. The Independent. 28 May 2014, London. [Accessed 3 Jan 2019]. Available from: https://www.independent.co.uk/news/world/europe/french-police-clear-hundreds-of-asylum-seekers-from-calais-migrant-camps-after-outbreak-of-scabies-9444407.html

Newton J. Migrants living in Paris refugee camp often used as a stop-off before heading to Calais and Britain are forced to move on by police over fears they were sparking a scabies epidemic. Mail Online. 1 June 2015, London. [Accessed 3 Jan 2019]. Available from: http://www.dailymail.co.uk/news/article-3105501/Migrants-living-Paris-refugee-camp-used-stop-heading-Calais-Britain-forced-police-fears-sparking-scabies-epidemic.html

For peer review only

For peer review only - http://bmjopen.bmj.com/site/about/guidelines.xhtml

Supplementary Table 1.
Standards for Reporting Qualitative Research [24] checklist.

No.	Topic	Page/line no(s)
	**Title and abstract**	
S1	Title	1/2–4
S2	Abstract	2/25–51
	**Introduction**	
S3	Problem formulation	4–5/84–102
S4	Purpose of research question	4–5/98–107
	**Methods**	
S5	Qualitative approach and research paradigm	6/135, 141–143
S6	Researcher characteristics and reflexivity	5/113, 131-132; 19/467–478<br>Sup file, 3
S7	Context	4–5/65–102; Figures 1 and 2<br>Sup file, 2
S8	Sampling strategy	5/114–125;Sup file, Sup table 2
S9	Ethical issues pertaining to human subjects	19/479–480
S10	Data collection methods	5/130–132, 126–141
S11	Data collection instruments and technologies	5/128–129
S12	Units of study	6/154–165; Sup file/Sup table 3
S13	Data processing	6/139–141; Figure 2
S14	Data analysis	6/135–139, 141–148
S15	Techniques to enhance trustworthiness	6/145–147
	**Results/findings**	
S16	Synthesis and interpretation	6–12/153–279
S17	Links to empirical data	Tables 1–3; 9/203–218<br>11/252–254; 12/274–276<br>Sup file 9–24/
	**Discussion**	
S18	Integration with prior work, implications, transferability and contribution(s) to the field	13–17/320–462
S19	Limitations	12–13/293–333
	**Other**	
S20	Conflicts of interest	19/493
S21	Funding	19/488–492

Characteristics of researchers

NR is a global emergency medicine clinical fellow with a Diploma in Tropical Medicine and Hygiene, and training in interview skills and qualitative analysis. SL and JM are Brighton and Sussex Medical School staff with prior research into institutional scabies outbreaks [3, 10, 12, 14, 41, 46, 47]. JM has previously done voluntary NGO work with refugees, and lived in physically similar informal camps to those described in the study.

For peer review only

For peer review only - http://bmjopen.bmj.com/site/about/guidelines.xhtml

Supplementary Table 2.
Facebook.com hosted groups in which the study was advertised

- Boat Refugee Foundation – UK Medical Recruitment
- Care 4 Calais Volunteer Chat Group
- CARE UK Charity (Refugee aid NW)
- Humanitarians in the UK
- Information Point for Lesvos Volunteers
- Junior doctors – refugee medical volunteers
- Medics for Refugees (former Medics for Greece)
- People to People Solidarity – Dunkirk and small camps
- RAISE – Refugee Action In Somerset East
- Solidarity for Refugees

For peer review only

For peer review only - http://bmjopen.bmj.com/site/about/guidelines.xhtml

Semi-structured interview topic guide

- Introduce self and project
- Confirm consent
- Explain that all responses are confidential and confirm what will happen to the data

Q1 Can you tell me what **experience** you have had working/volunteering in refugee/migrant camps and reception centres in Europe?

Probes: In which countries?
Was the camp/reception centre informal or formal?
What was your general role at the camp/reception centre?
How did you end up working/volunteering there?
Had you worked/volunteered in this kind of setting before?

Roughly how long (in days) were you working/volunteering?

How would you describe the camp/reception centre **as an environment**?

Q2 Can you tell me about your experience of **managing** and/or **treating** scabies in refugee/migrant camps and reception centres? If you were only involved in one of these tasks please also describe how the outbreak was managed or treated by others.
Who contacted you/first told you about it?
How did they describe the problem?
What did you do first?
What type of HCPs or non-HCPs were involved in managing/treating scabies?
What did you find difficult about managing these patients?
How did you feel about working with this outbreak?

Probes: Did you feel confident?
Did you seek help/advice?
What was difficult?
What worked well?
Who did you report to?
What would have made it easier to manage?

Q3 Can you tell me a bit more about how you made your **diagnosis** of scabies? If you did not make a diagnosis of scabies yourself, please describe how scabies diagnoses were made in the refugee/migrant camp and/or reception centres you were involved in.
Who did you examine and why?
How did you examine them?

Probes: Which parts of the body did you examine?
What equipment did you use?
Did you take a skin scrape?
How long did it take?
Who helped you?

What signs and symptoms did you come across?
What other conditions did you notice or consider?
What kind of residents had scabies?

Probes: What age-range?
How long had they been at the camp/reception centre?
Had they come from another site with a known outbreak?

Q4 What **treatment** did you use or consider?

For peer review only - http://bmjopen.bmj.com/site/about/guidelines.xhtml

Probes: Who did you treat?
What did you use?
What other options did you consider?
Why did you decide against them?
What advice did you give other staff working in the camp/reception centre?
What information did you get?/from where?

Q5 What, if any, environmental **decontamination** measures were taken to control the outbreak?
Probes: Clothes washing/replacement?
Shelter/bedding?
Furniture?

Q6 If you experienced **barriers** to treating/managing scabies in this setting, what were they?
Probes: Were there any barriers related to the:
Physical environment?
Legal status of the setting?
Availability of the medication?
Diagnostic capacity?
Staffing?
NGO structures?
Social structures amongst residents?
Languages?

Q7 What factors, if any, did you experience as **helpful** (i.e. facilitators) to treating/managing scabies in this setting?
Probes: Were there any facilitators related to the:
Physical environment?
Legal status of the setting?
Availability of the medication?
Diagnostic capacity?
Staffing?
NGO structures?
Social structures amongst residents?
Languages?

Q8 Is there anything else that you feel would be useful to discuss about scabies or scabies outbreaks in refugee/migrant camps and reception centres?

Q9 Do you know of any sources of data on the outbreak/s you were involved in that may be useful to our future research?

- Thank the participant
- Take contact details
- Make arrangement to provide results to participant

For peer review only - http://bmjopen.bmj.com/site/about/guidelines.xhtml

Supplementary Table 3.
Participant characteristics and settings.

**Sex**	
Female	10
Male	2
**Age**	
≤30	5
31–40	2
41–50	2
51–60	1
≥61	1
Unknown	1
**Type of healthcare staff**	
Doctor	4
Nurse	4
Allied health professional	3
Other (medical student)	1
**Number of years practising**	
1	1
2	2
5.5	1
11	1
12	1
22	1
24	1
40	1
Unknown	1
N/A	2
**Nation state of usual practice**	
UK	6
France	1
Belgium	1
Netherlands	2
India	1
USA	1
**Pay status**	
Volunteer	10
Paid staff	2
**Legal status of camp***	
Informal	9
Formal	6

** n=12, total >12 as some participants worked in multiple camps*

For peer review only

For peer review only - http://bmjopen.bmj.com/site/about/guidelines.xhtml

MSF clinical guidance for scabies [30]

All cases:

- Close contacts treated simultaneously
- Clothing and bedding changed after treatment

1st line:

Permethrin 5% (lotion or cream)

Apply everywhere on the body, contact time of 8 hours then rinse

One application may be sufficient, second application 7 days later reduces treatment failure

Or **Oral ivermectin** 200mg single dose

Single dose sufficient, second dose 7 days later reduces treatment failure

Not for pregnant women or children under 15kg

If 1st line treatment unavailable:

Benzyl Benzoate 25% lotion

Diluted before application use based on age

Apply everywhere on the body, contact time of 24 hours then rinse (contact time reduced for infants/babies)

Second application reduces the risk of treatment failure (eg. after 24 hours, with a rinse between applications or two successive applications 10 minutes apart)

For peer review only

For peer review only - http://bmjopen.bmj.com/site/about/guidelines.xhtml

THEMED QUOTATIONS FROM INTERVIEWS

Quotes from the thematic framework matrix, extracted from verbatim transcriptions of 12 interviews, 34–71min each (mean 47).

Description of the Camp Environment

General

P1: "It was quite distressing to look at really, and people were exploited."

"There was quite a few cameras from the newspapers... which was quite intrusive"

P4: "Can you imagine like frontline war correspondent, you know just getting there or I know frontline medic you know in World War 2, bit like that without the bullets"

P7: "Very confusing, very high stress levels and very difficult. It was a transient camp, people came straight from the boats, the tents, they were hypothermic, it was winter, so it was extremely transient and extremely a difficult situation."

P8: "It's horrible. There's only one water source for over two thousand people, there's not enough clothes, people are sleeping outside, there's not enough tents, the hygiene is terrible. I mean I was there in the summer so it was hot, there's not enough shading and people couldn't find a place outside the sun. It's more horrible now, as its winter, so it's freezing, and there's no heating. To be honest it's worse than the refugee camps I've seen in Africa"

P9: "I think they are a human rights violation. I don't think people should be living in them, especially in a first world European country this should not be happening. People are living in cold tents without heating and electricity, there's rats, there's scabies, there's a lack of toileting facilities, a lack of hygiene, lack of food."

P10: "You don't see why everyone says it's horrible, it doesn't seem that bad when you first arrive, but when you start talking to people who live in these camps every single day, you see how it crushes them over time."

"The other thing that really struck me was how gender based vulnerability was accounted for in practice. When it snowed, they kept moving women out to a better and nicer camp, but men get cold to yet they only moved out the women. It sucks to be a man."

P11: "It was like an emergency pavilion... they put 3,000 refugees together in a big camp... Every tent, there were 10 people sleeping, and they went eating in a large room, where they could eat together with 100s of people. They have all the place where they can go to the toilet, and places where they can wash themselves, but it was all outside, it was inside they could sleep and eat, but they can wash themselves outside, and they could to the toilet. It was all arranged, but it was very basic."

Shelter

P1: "It's very organised... you think you're in a small village really... but then when you go beyond that you see just a sea of tents where people just live"

P2: "There's not even proper tents or anything, they just use whatever they can find"

"In Serbia it's almost like a jungle, so it depends on like how lucky you are. So, some refugees have tents, some doesn't have tent. And then sometimes they just sleep in the field because they walk to the border and sometimes they just stop in between and they sleep like under the stars."

For peer review only - http://bmjopen.bmj.com/site/about/guidelines.xhtml

P3: "There's no shelter... there's just literally people sleeping in bushes"

P4: "There are no more camps, they're gone... so we have like, nine hundred refugees living rough in Calais and then we have probably about four hundred and fifty refugees living in the forests of Dunkirk."

P5: "People live there with tents or without tents."

P7: "The camp was steadily improving by the volunteer and NGO work, and residents work, it was not as stressful environment as the factory hole which had been utilised in the first as tents... It was not a pretty environment but a lot of effort to make it a habitual environment and make it was a better environment in the end."

P12: "Different locations so it was the side of roads mainly, and next to the roundabout there would be a patch of grass and there would be a large group of people on the grass... then at another location might be in kind of a residential area but at the back of that there'd be a field, just some woodland and then there'd be a large group of people there as well so it was, it was groups of people scattered throughout Calais. It wasn't a camp"

Water and Sanitation

P1: "Running water was in troughs, like animal troughs. People washed in the open, or didn't wash at all. They didn't have any clothes washing; there was no way... People would just wash their clothes and just hang it over a bush or something"

P2: "There is no proper latrine system"

"In Serbia, there's one German NGO that are giving free showers every second day for refugees. They also provide some water so the refugees can wash their clothes and stuff. But, in general, in one setting they can only give showers to up to forty people max. So, there are like one hundred and fifty to two hundred people, they could only give showers to forty people. So, some people might not be able to do showers, like maybe you can do once in a week. The sanitation is quite bad, and even the water, sometimes there is no water, if this NGO doesn't come and provide water, so they do take water from some cart, but it's not really clean"

P3: "There are portable toilets there, but that's about it, really. Occasionally an organisation, like Calais government, kind of, a local authority, they occasionally bring, I guess you would describe them as, not showers, but more like taps, portable taps with cold water, so people could have a kind of a quick wash and things"

P6: "It was quite well established... having said that, facilities were still really quite basic. The toilets were just portaloos, there was very limited access to showers, and running water and it was very muddy"

P10: "I never stepped inside any of the facilities in the camps, I smelled them as I walked past but I didn't have an incentive to go inside so I didn't. I assume they were quite terrible, like I could smell them, but not good, I did not see them, they were not good, I didn't want to see them."

Food

P3: "There was the food distribution which was run by volunteers so there would be a van going twice a day to camps around Calais and once a day to Dunkirk. But apart from that, there's very little"

Safety

P1: "There was areas there where the women and children stayed that was quite well organised and had security and were patrolled... but a lot of people didn't want to go in there, and often families were separated"

For peer review only - http://bmjopen.bmj.com/site/about/guidelines.xhtml

P2: "Often the riot police, they're called the CRS, they have these big vans and they would often kind of hover around, trying to intimidate the refugees and the volunteers, and so that didn't kind of make for a very nice atmosphere it was quite tense"

P5: "It feels like the woman, she is not completely safe because they protect themselves, they are old, sometimes there are pictures of walls falling down. It's not safe also because there are a lot of tensions so sometimes fights happen."

P12: "People were hiding from the authorities... because tents were being routinely slashed and belongings were being destroyed by the police and authorities"

Healthcare

P2: "Those camps in Turkey, the settlements, they are really in bad shape, no NGOs are running in, no-one to give free food, no-one to give the healthcare. I mean we were there giving free healthcare, but basically we didn't go and visit them every day, so they'd basically sometimes end up not having at all, and they were refused to get healthcare in normal hospitals"

P10: "You start to see how someone with heat failure isn't able to access treatment - it's crushing him. A person with diabetes can't get their blood sugars up - its killing them."

"A lot of organisations can be disrespectful, especially the doctors, they can be very humiliating, that makes people reluctant to go back for treatment. And they can also be rather indifferent to a lot of things, which means things don't get taken care of. Refugees wait in line for several hours, to be seen, to be disrespected, so they don't go back to the doctors."

Barriers

Water, Sanitation and Hygiene Facilities

Washing of clothes

P1: "Clothes that they hadn't been able to wash, or they only came over in their clothes that they were standing in"

"There wasn't anywhere that people could wash their clothes, yeah, have washing facilities to wash their clothes... They wanted to be clean. And it's very difficult to keep clean if you're living outside in a tent"

P2: "Most of the scabies has gone so bad, the scar has got so infected because of the very poor clean and hygiene. They've been wearing the same pants, sometimes if it's raining you get wet and you don't have any new clothes so you just wear the wet clothes all over again."

"They might have some utensils to cook, but not enough to boil so much of water, or somewhere they can soak all the clothes and to wash them. And even if they hand it all to the volunteers, even we don't have the capacity to disinfect all the clothes"

"Sometimes they don't have water...it's quite tricky to wash the scabies infected clothes. If you have big washing machine that has a warm water setting, that would be great. But some of them doesn't have it, so you have to boil water and soak it or you have to put chemicals and stuff... the chemicals that were needed, you don't know where to get them, so you need to get support from the larger NGOs. If you don't have larger NGOs then you might not be able to do it"

P3: "People have so little and they don't want to give any clothes or bedding that they have up... definitely not clothes washing"

P4: "There was like cold taps there that they could wash with to wash it off the next day, but my problem with that was the clothes that they're putting back on. Yeah, the clothes have to be washed at a high temperature. So it's very much a struggle"

For peer review only - http://bmjopen.bmj.com/site/about/guidelines.xhtml

"the treatment we were giving was that because it was toxic we told them you have to wash it off the next day, that they could shower it off, but that's all. They put back on infected clothes or they'd dry with infected towels"

P5: "Then we have other issues which is means we have to get clothes, new clothes for these people and new sleeping bags because, and then we cannot tell them to wash the sleeping bags in the residence at 60°, it doesn't work because they're homeless.... they can only wash their clothes by hand so of course they cannot wash everything that there is... we cannot provide clothes for all of them."

P6: "Even if a patient is treated for scabies, part of the treatment is also to wash the bedding, and all clothing in hot water, dry it in a drier picking up all the scabies mites. And those facilities weren't readily available for anybody at the camp... I still think that even if we'd of had a sort of endless supply of Permethrin or Ivermectin, I think it still would have been problematic with this problem of re-infection."

P8: "The challenge was to do the hygiene part of the treatment... The problem was to find enough clothes for them to change their clothes because there is no washing thing in the camp so we would have to do the whole thing of putting the clothes in a garbage bag, close it for 72 hours, then take it out. Then we actually found out that a lot of people don't actually have a spare set of clothes, or they have a sweater and trousers but don't have more underwear."

"There's only one water source for over two thousand people... there was no washing facilities for the people, they had to wash their clothes by hand. There was no soap provided. People did not have the possibility to really wash their clothes well."

P9: "They couldn't wash and we didn't have enough blankets to give out, so everything was getting re-infected all the time. We couldn't take everybody's clothes because there wasn't enough clothes to give out again so that was really hard to manage... Things were just getting re-infected because it wasn't very well controlled."

P11: "Insufficient washing and bedding, and like not enough washing machines... a lot of people, most of them only had one pair of clothes, and they can't change them, when they treat. We need clean clothes, so that was a difficult part... You also have to think about the hats, scarves, they also have to wash them, because in the beginning, that was a thing we didn't clean the hats, scarves."

P12: "Most quite overwhelming was the lack of amenities, people's clothing, sanitation facilities, so no showers, no toilets."

Shower facilities
P2: "There are one hundred and fifty to two hundred people, but they could only give showers to forty people. So, some people might not be able to do showers… the sanitation is quite bad, and even the water, sometimes there is no water. If this NGO doesn't come and provide water, so they do take water from some cart, like not really clean and it's not really in a good shape."

P3: "Not having access to showers"

"That would be the only way to get rid of scabies. But this doctor said that he was already, I mean it's a small clinic, he was already overwhelmed, he was saying he'd have to… They only had two showers, there's no way they could do that, he just wouldn't be able to cope with that many people."

"They don't have showers, or they don't let the refugees use the showers."

"At least in Calais they have these kind of portable taps, shower things. That's not provided in Dunkirk, the council in Dunkirk won't provide that for the refugees. One of the main barriers we faced was getting proper showers to people, or people to the showers."

For peer review only - http://bmjopen.bmj.com/site/about/guidelines.xhtml

P4: "Lack of showers and lack of laundry meant that you couldn't focus on one. Okay, here's a tube of scabies treatment, it's quite toxic so you put this on your body, rub it all over, twenty-four hours later you wash it off, but all your clothes have to be clean and they'll look at you like really how. It's a very, very uphill struggle to treat it and it's really prolific."

"They did have that facility [clothes washing] in the Dunkirk camp but, towards the end of the camp, before it burnt down, they stopped washing clothes and they stopped, they turned off the showers and then scabies started there as well... In the beginning in the Dunkirk camp it seemed manageable. When we were there we hardly saw any. It was under control, but then the minute you put people out there in the middle of a forest in filth and dirt."

P5: "There is really no shower and there are some areas that are a kind of shower area, but it's not really a shower and its cold water. Basically it's not helping at all... it's very, very difficult to stay clean."

P6: "Treating scabies was problematic because as you're probably aware, with the Permethrin treatment, the lotion, you're supposed to keep it on for 12 hours and then wash it off after a 12-hour period, and access to showers was not always that easy... I never actually saw the shower facilities there but I understand they were very basic and there were often very long queues for the showers. So it wasn't easy to shower."

P8: "There is not even one shower, there's no soap to wash yourself... people couldn't even wash their bodies with soap."

Sharing of belongings
P1: "Charities came in to give clothes, but the clothes weren't ever washed and when someone went across the border, tried to come into England, all their stuff was left, and whoever was in the tent just used that"

P2: "The main biggest problem is to get them to improve their own hygiene... to get them to clean themselves, and have each one have their own towel, and not to share them."

P8: "A lot of people basically only had one pair or underwear, or socks, or they couldn't wash their shoes... they usually only had one pair of blankets... There would always be something that wouldn't be replaceable... Because of lack of things for themselves, they keep borrowing each other's clothes and blankets."

Healthcare factors
Availability of medication

P2: "Limited availability of medications."

"In Turkey, we don't have big NGOs because they're not allowed to be… so that was a bit tricky. We need get all this medications on the camp by ourselves. It's also depends on what is in each country. In some countries they use Benzyl Benzoate, then some of the countries Permethrin, so you need to know which one that they use for that country and what's familiar to that country. Then you can source it locally... Some people smuggle it in the bag, and they took a flight and then they give it to the organisation. In the long run it's not going to work because when you run out and you change to different regimes that's a bit complicated, if you stick to one thing that's easier to source."

P3: "The medication was very expensive... there's a lot of people, nearly 1000 people there in Calais and Dunkirk, that's an issue."

P4: "We don't have the treatment for it, because we don't have the money to buy in the treatment."

For peer review only - http://bmjopen.bmj.com/site/about/guidelines.xhtml

P5: “Only one we can buy in Greece is the more difficult to apply and the one you have to apply many times.... so there is less chance of success of people really following how to do it.”

P8: “We didn’t have enough medicine most of the time... what we would do is write down their names and a week later if we got the medicine we would ask them to come back... Because we were short of pills we would just give it once.”

“In the beginning they trusted us, after a while they didn’t because we promised them treatment and we didn’t give it.”

P9: “Just didn’t have enough treatment... we have to fundraise to buy it, and if we don’t have funds we can buy it.”

P10: “Permethrin in pill form [we presume they actually mean ivermectin], they don’t sell that in Greece, that would be the most convenient way to provide medical treatment... X had a stock from Netherlands, but as soon as that ran out it was gone. The only treatment that is available is Benzyl peroxide, but you have to dilute that to make it the right concentration, and dilute it differently for children, then leave it on for 24 hours or at least overnight. There was only one pharmacy that would only sometimes have it in stock, so we had to arrange with them beforehand... The medication available is the least convenient medication you could use. If you could use a pill for everyone that would be so much easier.”

P12: “The lack of cream or treatment... Because none had been donate. We didn’t have any in the container, and we just weren’t able to get hold of any. The only medication we had was what was in the container, which I believe was from volunteers bringing over all donations. It was quite limited really.”

Lack of privacy for medical exams
P2: “Don’t have any proper building, clinics set up. So, everything sees what we are doing, so if someone has to show something we don’t have a closed examination room, we don’t have a private examination... you can’t really examine them, like you can’t really touch and see in your own eyes, you have to rely on the photos because they’re too shy to show it to you.”

P4: “You’re out there in the middle of, you know, you’re not somewhere quiet and private you can’t ask people to strip off.”

P12**:** “If you were treating somebody, there certainly wasn’t any privacy for people... you’d find there’d be sometimes, gathering around and watching you... amongst large crowd.”

“I don’t think we were openly saying to people, “Oh I think you’ve got scabies,” just because we didn’t want to say in large groups of people and I don’t think we wanted to publicise that type thing because you don’t want anybody to be offended or people, other people are listening.”

Lack of staff with experience of scabies
P2: “Most of the Western doctors are European, they’re not very used to seeing scabies in normal settings.”

“They have that scabies and it gets really bad and infected, some people might mistake it for like normal infected things... they don’t give the creams for scabies and stuff.”

“In Greece I met a lot of UK doctors. I find most of them, they know scabies and stuff. But when I was working with in Turkey and in Serbia, I worked with people from Europe, like Austrian and Germans, and then some of them had never seen scabies before. Because it’s not very common in these kind of areas... so in terms of that staffing, it’s not really hard to get staff, but it’s really hard to get staff that have seen or have managed scabies before.”

For peer review only - http://bmjopen.bmj.com/site/about/guidelines.xhtml

P4: "Most of the other first aid people they'd never seen scabies in their life."

P8: "We heard they say they haven't seen scabies which could mean they don't know how to recognise scabies or they don't want to, so could mean the other doctors in the camp don't know how to recognise scabies."

"Many people came to volunteer only for a few weeks so a lot of them didn't have much experience to recognise it."

P10: "European doctors did not have a lot of experience with scabies, so I think a lot of them didn't realise what they were seeing."

"A lot of volunteers had never seen scabies before... volunteer based organisations take people that want to volunteer, but because they need people they're not really in a position to screen them for the job. I've definitely noticed that there really isn't a lot of experience of doctors with scabies."

P12: "I don't know if I ever seen scabies before so I felt quite inexperienced."

Social factors

Language barrier
P2: When I was working with the Afghan refugees, one of the time we couldn't get a proper translator... and Didn't want to tell the other people they had scabies and what's going on. So, they feel reluctant to comply, and they're like no, it doesn't work. If you don't have proper translators to translate anything to you that also could be a big barrier to understand what's going on really."

"When I was working in Serbia we don't have a proper translator, so we were using one of the refugees to translate for us, which is not very ethically correct I would say, because of the confidentiality issues"

P3: "There were a lot of people, their English wasn't great and we rarely had someone on the team who could speak Arabic for example so sometimes it was difficult to diagnose someone... it was difficult for them to explain to us, and for us to understand exactly what the problem was."

P4: "We didn't have all translators... so that was an issue. We sometimes had to ask another refugees and call out for somebody that could come over and help and speak enough English to tell us what was wrong with this person... I couldn't explain to him what I could do so it'd just be, just frustration."

P6: "We didn't have proper translating facilities. Trying to explain all of what I've just explained to you about washing off the treatment, the lotion and washing bedding and clothing, even if that was all possible, explaining all that to people without proper translators was difficult."

P8: "It's hard, because we were a volunteer organisation we didn't have official translators. We would always ask people in the camp to help us translate. Sometimes it was difficult to find translators in certain languages, like Kurdish. In the end that would mean that the Kurdish people have less access to our care than Arabic or Farsi speaking patients."

P9: "We usually have translators... but you don't always have the right translator."

P11: "When you talk to 10 people at a time, and both women and men together, it's maybe difficult, and there are also people who had another language, and some languages are not known in other translator centre, so we couldn't get a translation for them."

P12: "Language barrier"

For peer review only - http://bmjopen.bmj.com/site/about/guidelines.xhtml

Stigma

P2: “To ask them to, not to share things with your friend, try not to sleep with them, I feel bad sometimes. They sleep together, they have like one blanket and all sleep like five people on the same blanket. So, when you say okay, you have this problem, you can’t sleep with your friend, it’s almost like giving them a stigma.”

P7: “Some people had social barriers, as in I am so clean and I don’t get this kind of thing... "I have eczema", or "I have dry skin" or this or that, "I don’t have scabies". Those were the people who often suffered for the longest... Possibly as a result of stigma surrounding scabies... Scabies and mites are not very pleasant things to have.”

P8: “didn’t want to tell the other people they had scabies... there is some sort of stigma of course if you have some sort of rash that you are not clean or something.”

P11: “it was difficult because the Syrian people looked at Eritrean people like they were dirty… because they had scabies.”

Non-compliance with treatment

P2: “This whole effort has to be put by the patient, and under these circumstances this patient has a lot … a lot other things to think about rather than this itchy thing ... The compliance is really hard, and also to get them to continue the treatment over a few days, also quite hard as well.”

P5: “You have to treat case by case explaining to them how to do the treatment with the message of giving them new clothes and sleeping bag and that are shown everything and even when we try to explain everything they don’t always do it correctly. That’s for a major issue because if the treatment is not done correctly then it won’t be effective.”

P7: “The one thing which we did encounter occasionally was people that didn’t listen to us, so I would say to put the stuff all over your body, head down to the toes and they got it everywhere... to do it all in one night and keep it on for so many hours and then they walk away and then only put a bit of cream here, and a bit of cream there, for a week and then they come back and it hasn’t got better.”

P8: “When they would hear about the whole treatment thing that their whole tent has to be treated or it doesn’t make sense, then they would say ok never mind and go away and don’t come back. Or people would come and say that their roommates don’t want to come, or they would bring their roommates and then the roommates would say oh this is too much work, I don’t have itching so I won’t take part in this.”

Population Mobility

P2: “You have to trace who might be the contact... in the refugee setting you have to also to treat patients that might be from the same family, and they just move around. You can’t really tell who has slept here, who has not slept here, who might possibly get it.”

P3: “Not being able to kind of, you know, everyone’s moving around Calais all the time, so not being able to kind of, I guess, not having the resources to be able to keep track of individuals with scabies.”

P5: “You have always new people coming in, and people leaving so it means that even the people treated, if they come to you and you treat them and then they disappear, not having any other treatment.”

“Making one day of treatment of scabies is not successful because the population keeps changing... all the energy and the money spent from the treatment day actually is a kind of a waste... in this population which is always changing, it is not a good use of resources because we won’t be able to properly medicate it.”

For peer review only - http://bmjopen.bmj.com/site/about/guidelines.xhtml

P7: "Often these [single young men] were the most transient people who would simply wander off at some point, and not come back for a second dose or not come back for collecting whatever they're supposed to collect"

P8: "The fact that people move in the camp is also a big challenge because even if you treat a whole container, if one person is put there by the police and if they say this person has to sleep there and this person has scabies then the whole effort is for nothing. So that's actually one of the biggest challenges, that people are moving all the time."

P11: "It was difficult in this setting, because you can see there are 3,000 people, and everywhere probably 10 people in and 10 people out... it was also very difficult to treat them well, because normally we have to treat everyone together, at the same time."

"It's difficult to treat everybody together, and there's nowhere to isolate people, a lot of mobility of people, and logistical problems with that."

Organisational Factors
Living conditions – dormitory style rooms
P2: "In the government run camps they usually have their own tent or their own cubicle but in, in the jungle settings most of them live in quite overcrowded, they all just live, sit together and stuff."

P3: "Barriers in terms of the crowded conditions... I think just in terms of people who are living close together so scabies is very easily spread."

P6: "People were living in fairly cramped conditions which obviously made transmission a high possibility."

P7: "The most difficult to treat was the single young men, of being in the large accommodation, and camp accommodation, while the families, were in the family rooms. The single young men were in large dormitory style rooms and so it was a lot more difficult to determine the close contacts."

P8: "A lot of people were sharing tents or containers with large number of people."

P10: "Sometimes you would have people living in bigger units... and people would partition of with blankets where different families lived. That was really difficult."

Poor coordination between organisations
P5: "I remember one guy that they sent with everything for the scabies treatment and because it was my first day I was really trying to do it the way it should be done... He had an appointment for the day, so I wrote on a bit of paper and I wrote please allow this man to have two showers because he needs to do the lotion and wash it, 8 hours later. I sent him with a paper and everything. He had the new clothes, to bring back. Then they didn't allow him to have the first shower because he had scabies. They sent him away because he had scabies, even though he was coming to get the treatment."

P8: "A lot of things are managed by volunteers. Ideally in the camp you would want to have one named medical actor providing healthcare and asking for specialist to come and do clinics or to ask for advice from other organisations but there was no governmental healthcare facility. There was a huge gap because it didn't even exist, so other organisations would come in and do little things but the whole coordination between us and the others were so strange... there's no continuity. It was really unorganised."

"So many different organisations with people coming and going, also the paid staff did not stay longer than 6 months to one year, and so many volunteers coming and going - there's no continuity. It was really unorganised."

For peer review only - http://bmjopen.bmj.com/site/about/guidelines.xhtml

P10: “Lack of coordination and appropriate communication. I don’t think it’s the resources, the resources are there they're just not put together in the right way.”

“Topical steroids was what was given for symptom relief because they couldn’t give anything else, proper treatment required too much coordination.”

“The medical organisation in Moria didn’t communicate with non-food item NGOs, so due to lack of coordination he could never have medication at a time that he wouldn’t be at risk of re-infection... X used to write prescriptions for non-food items that weren’t honoured by the non-food item NGOs.”

P11: “There was another NGO who worked there, and sometimes they did their own things, their own plan. Sometimes it didn't work well together.”

P12: “Clothing was distributed, but we weren’t really able to coordinate that because we, we didn’t really have communication between the individuals and the charity.”

Lack of support and poor treatment from authorities
P1: “There wasn’t anybody in charge of this camp, to tell people that there was a scabies.”

“They were hidden, you know, thousands of people hidden in one corner of France that one-one wanted, the whole world didn’t want to recognise.”

“Because the French Government didn’t want the camp there, they weren’t doing anything. There wasn’t even... basic facilities... They didn’t really want to provide healthcare for people. There wasn’t any shelter, there wasn’t any child protection, there wasn’t any protection for any vulnerable people there at all, adult protection, there wasn’t any of those type of things there.”

P2: [informal camps] “What the government is trying to do is, they’re trying not to get the refugees too comfortable, so sometimes they cut like water, they try not to get us to be able to give out clothes, they raided our distribution place.”

P3: “I mean a lot of people avoid going to anywhere where they’ll interaction with the authorities because, because of the police brutality, they’re worried about how they’re going to be treated and worried about being deported. Even though that can’t happen at hospitals and things, and people at this particular clinic, you know, they’re running it for people like this and they’re very nice, but there’s still that perception. I think a lot of people try to avoid going.”

“The refugees have their bedding and their coats and shoes confiscated by police at night.”

“We had to take someone to A&E once, and he was treated awfully by staff because he was a refugee... what we saw it was just the refugees being stigmatised at being refugees.”

P4: “In France you’re forbidden by law to give first aid, well not give first aid, but to give prescription medication, my hands were very tied into how to treat it.”

“Until authorities do the right thing and take care of these people properly you can never, will eradicate it. It will carry on as long as they can’t shower properly, as long as they can’t get clean clothes and wash the clothes the scabies won’t go away and the trouble is that the authorities in France and in Greece and even in Italy do not care and they’re not bothered and they don’t wanna know and basically refugees are depending on volunteer groups and small charities for their wellbeing and they’re very limited.”
P6: “Local authorities were not getting involved because the camp was informal.”

P8: “This official organisation that was responsible for that kind of camp, there was one doctor who was responsible for the public health in the camp, he was responsible but most the time he wasn’t

For peer review only - http://bmjopen.bmj.com/site/about/guidelines.xhtml

there, he and the other organisation were ignoring that there was scabies in the camp... The Greek doctor kept saying it's not a life-threatening disease, yes its annoying but there are worse things to care about, but even the worse things weren't cared about... The people responsible for it would try to ignore it to ensure that they won't have to deal with it... in the meantime there was lots of suffering."

The care in the camp was very limited. In general, the government would be responsible to provide the healthcare but the government wasn't there so they had contracted [healthcare] out... but they were always short of staff and short of a lot of things.

P9: "In the official camps if scabies happened it would be much easier to deal with because they have some washing facilities and they've got like WaSH and that kind of organisations working there. I was working more in non-official, and where it was happening more for me was nonofficial and we didn't have that support."

P10: "Lack of will and commitment."

"A lot of organisations can be disrespectful, especially the doctors, they can be very humiliating, that makes people reluctant to go back for treatment. And they can also be rather indifferent to a lot of things, which means things don't get taken care of. Refugees wait in line for several hours, to be seen, to be disrespected, so they don't go back to the doctors."

P11: "They were just logistical problems, sufficient manpower, sufficient washing machines, sufficient bedding, who cares for this? It certainly wasn't a priority for the organisation, because people have itching, but they are not going to die"

"The responsible authority, the central body for reception and asylum seekers... they don't support it."

P12: "People were hiding from the authorities... the settlement or their place where they would sleep would not be there because tents were being like routinely slashed and belongings were being destroyed by the police and authorities."

"What would have made it easier to manage?"
P1: [Washing facilities]
"I think it [washing facilities] would have made it easier to manage, because people could wash their clothes, and the people wanted to wash their clothes as well. They wanted to dry them, they wanted to wear clean clothes. They wanted to be clean. And it's very difficult to keep clean if you're living outside in a tent."

P2: [Washing facilities; Public Awareness; Divided living areas]
"If you have a proper washing machine that have hot water then you can do it, or you can ask the refugees to do it themselves, but if you don't have that then it's really hard to be able to eradicate it."

"It's not hard, as long as you educate them and try to make them understand that what's going on, this is what needs to be done."

"For public health education you'd probably want to print out like a really easy version for it, or maybe you have to make a new one so they can hand out to the refugees to say we have these issues, and this is what happens if you are itching."

"If you have like a proper segregations of rooms, or cubicles for each family, that will be a really good point to really manage it. You can just do entire families. It's easier to also plot where the scabies is, you can trace them, where they go and what the patterns... But in the jungle they just get it like a wildfire."

P3: [Coordination of organisations; Access to resources]

For peer review only - http://bmjopen.bmj.com/site/about/guidelines.xhtml

"If we could kind of put a system in place, if we worked with X, one of the main organisations at Calais, they have a warehouse and do clothes distribution... to see if we could organise, maybe have a ticket system so organise getting people treated at the clinic so with cream or tablets, and then on the same day giving them a shower, clean clothes, taking away the old clothes to burn... giving them clean clothes, new clothes straight away and giving them a ticket so they can come back the next day for another shower 'cause you need to, and then to completely get rid of it, and making sure everyone in the camp does that.

"Access to resources... showers, clean clothes, and cream."

P4**: [**Better living conditions; Washing facilities]
"If they had somewhere decent to live... If they had somewhere with a roof over their heads and if they had somewhere where they could have a hot shower and somewhere to wash their clothes."

"I did think at one point I wonder if I could get like a mobile clothes washing van or something, it crossed my mind that it could be one thing we could have done with the treatment - get the clothes washed, shower, clothes washed, but it didn't happen."

P5: [Screening on arrival; Washing facilities]
"When we have treatment and sleeping bags and clothes, it's easier to manage because at least we can do something."

"The only measure that you could take is to take every new arrival and any sign of scabies and treat them, but it would take a lot of time to get sorted to do it. It is what they are doing in Serbia where all new arrivals with scabies, is quite a lot. We give all our new clothes and sleeping bag."

"If we could have a place to send people where there is a washing machine and dryer and maybe a shower, in this case we could treat more because we could have, like the new clothes but if all clothes that people wear, and the sleeping bags and blankets."

P6: **[**Oral medication]
"Availability of Ivermectin may have, to some extent because it doesn't require washing the lotion off afterwards, you know it's an oral treatment, a single dose oral treatment, may have helped but it wouldn't have really have overcome the problem of reinfection."

P7: "Nothing in particular - it wasn't a problem."

P8: **[**Screening on entry; Cooperation of organisations]
"In every camp where people arrive there's always a medical check for all the people so that would be a great time to check everybody for it, before they allocate them in a tent."

"Cooperation of the people that manage the camp because they could help by not moving people all the time, or always discuss it with a public health responsible person in the camp, but there was no such person. Making sure to allocate people in the right place to not disturb the scabies treatment, that would be great. Working together, putting together resources, but the first thing is to recognise that there is a problem."

P9: **[**Better living conditions]
"They shouldn't be living in these conditions in the first place. They should be in homes and houses, not refugee camps and abandoned buildings."

P10: **[**Interpersonal communication; Coordination of organisations]
"I think it's really important to train humanitarian workers in communication and interpersonal interaction... You have to really care, you have to understand that the other person isn't always going

For peer review only - http://bmjopen.bmj.com/site/about/guidelines.xhtml

to understand right away and you have to work with them... There's a lot of conversations that need to be had."

"You need to appreciate what others in your NGO and what other NGOs are doing, and be conscious of the fact that it is a collaborative goal."

P11: [More resources; Systematic treatment]
"If we had the time, the people, and just one tent more, then it would be no problem anymore because you had to do one day, very intensive, and a good job, and then you have not left problems... The plan I made I think it would be good, if there was money for it, if there were the people for it, and if there were an extra tent over there, then it was managed in one day, in two days. Then we would have to keep doing it, that every person who came in, and had the same treatment. You had first to do it with all the people who were there, Eritrean people, and after that you had to do every Eritrean who came in, we had to give them treatment, and then the problem was solved."

P12: [Coordination of organisations]
"We could organise what the warehouse felt getting them fresh clothes, but then we'd have to also organise things like showers."

Facilitators
P3, P4, and P12: "nothing"

Water, Sanitation and Hygiene
P5: "What was really helpful is that the centre where people could go with a shower, and have a washing machine... they can get a hot shower two times a week and they can also wash their clothes over there. I mean if we provide one set of new clothes, we can help them to wash the clothes they have. Twice a week is not enough to really make the treatment as it should be, but it is very good, because before we had only places with appointments for the shower, there was only one shower. There we can be sure they have their follow up of 10 days so they can get the treatment, the new clothes, the new sleeping bag and take to the shower."

P7: "People have access to showers so that was not a problem, clothes had to be washed in the washing machines… and bed sheets and everything else was taken out and burnt. That was the protocol and we didn't have a persistent problem, we didn't have outbreaks."

P9: "There was mobile showers we were getting access to, not everybody could get access all the time, so that was still difficult but there was that there so it's better than nothing."

Coordination of organisations
P1: "What we suggest we do is that they have clean clothing ready so that when they go to the Government organisations, health organisations for the scabies, that they can actually start treatment that evening, and we explain how they do that."

"We referred a few people to A&E because they were quite unwell. We gave letters to people about scabies to bring to the medical place the next day. We made sure everyone had a letter saying it was scabies, and documented when it was and how many people were in their tent etc."

P2: "We worked a team that provide showers. On one of the days, we call it a scabies shower, so where we have all the scabies patients. We give them a card that says if you have scabies you get showered tomorrow. We give them a card that says they have scabies, we give them a cream 24 hours before and then asked them to get showers, and then we put the creams again. So we partner with shower teams, and also, we partner with people from the distribution team so we could get them new fresh clothes. I think the treatment of scabies, it has to be like not just a healthcare provider, and it has to be like non-NGOs to help you work on that as well."

For peer review only - http://bmjopen.bmj.com/site/about/guidelines.xhtml

"It's really good to liaise with the large NGOs, that could provide, most of the time they [medications] were available. They have all those creams."

P3: "NGOs were more of a help, as I said X said that they could provide clean clothes and they could work with us."

P5: "Make a letter for them saying that this person has been diagnosed with scabies, please wash his clothes, allow him to have a shower and we will make a thing that it's not as contagious and that you cannot catch it like this. It's working well."

P7: "There was the camp administration which was run by a local NGO and the staff were aware of the problem and were good at directing people to us if there was a problem... we gave the patients who were referred slips to take to the administration to get new blankets, new bedding and that and take the old stuff away for washing or burning and give access to machines for to the washing."

"They have no difficulties staying on top of it, because the camp administration are behind it and the residents are behind it... That was all positive... the support from the camp administration."

P8: "The willingness of other organisations like the warehouse on lesbos that provided us with clothes."

P10: "Rented them clothes... built up an inventory of clothes, I worked with contacts in the warehouses to get donations so we could ship them over so we could lend them clothes based on their size/gender etc."

P11: "We had a meeting with them [NGO]... they had their own plan, and it was different from our plan. But we talked about it, and then it's okay. We gave everyone a clear task and then the problem was solved."

Education
P1: "We explain how they do that [the treatment] … and then for those that have been infected, for them not to shake hands, not to share bedding."

P2: "If you educate the patient very well, make them understand very well what's going on, and what needs to be done, I think that also worked very well... They understand, and they start to even manage to recognise their friends who have the same thing, which is really useful."

P3: "Friends might have been able to tell each other. I don't know help diagnose each other or encourage them to go to the clinics."

P7: "One treatment which was once easily explained and where the neighbours could explain to you, and everyone could explain to each other and reinforce the message which we gave every day."

Language facilitators
Translators
P1: "We had lots of translators there. We had our own translator who spoke several languages, and there were people who lived in the camp, working or volunteering as translators, and they were actually very good. Certainly, got the message across. So, there wasn't a barrier."

P3: "Some people did speak very good English and often if someone didn't, they would get someone who they knew did speak English and they would come and translate, so that helped."
P6: "Sometimes there were people that helped with translation, other occupants of the camp, other migrants would help with translating, but that wasn't consistent help it was just if they were around at the time and they offered to stick around to help a little bit.... if we did have people to translate then we were able to explain what the probable cause was of the itching and I suppose in itself having an

For peer review only - http://bmjopen.bmj.com/site/about/guidelines.xhtml

For peer review only

explanation can offer some relief to people, and also reassurance that there wasn't anything serious in terms of, you know life threatening."

P7: **"**They had very good translators for the other doctors and nurses."

P8: "A lot of people came to help us translate... The few people who spoke English would always be after other people to translate. Many people don't have anything to do all day so they'll help to translate for other people."

P9: **"**There's a range of languages, if somebody knows both they can tell us a bit more."

P10: **"**We were very lucky because X worked with unofficial translators from every community in the camp, so they were a great facilitator for getting the message across."

P11: "The language is difficult, but we talked with the translator, with a live translator."

P12: "There were a number of people there who had been residents in the UK but then had been deported so often there was somebody who could speak really, really good English. They would act as a translator and that was really helpful."

Voice messages
P5: "Voice messages in different languages."

"I tried to treat and tried to improve the way we are treating, to make it clear for the people in their language. So we have voice messages explaining how to take their cortisone treatment and how to apply the treatment. Basically for each person that we treat, we say in the voice message and ask which one [language] they prefer and then we play the voice message."

Information sheets in different languages
P5: "We do have information sheets... we show them with the data, and the map to go to the shower place and there if this is written in Urdu, then we will have some positive treatment."

Incentive to take part in treatment
P2: "The best way that we come up with is just get them to get rid of whatever clothing and bedding, and then we give them replacements back. So there's an exchange there. That's the only way that we can minimise the spread and stuff."

P7: "The patients knew that it could be treated, some used it possibly as a way of getting new things and were quite disappointed if they didn't have it, but that didn't happen that often either."

P8: "Some people when they found out we would give them another set of clothes and we would buy underwear for them, some more people were interested as they had an incentive to take part in it."

P10: "We would take back everything except the underwear, so they got to keep the underwear, which for a lot of people was like an incentive to participate."

General
Division of living areas
P2: "If you go for like a government run camp you have proper dividing, like in Northern Greece, they have cubicles. So, usually the outbreaks are a lot smaller and usually limited to a few cubicles, or maybe just one cubicle."
"If you have like a proper segregations of rooms, or cubicles for each family, that will be a really good point to really manage it. You can just do entire families. It's easier to also plot where the scabies is, you can trace them, where they go and what the patterns... But in the jungle they just get it like a wildfire."

For peer review only - http://bmjopen.bmj.com/site/about/guidelines.xhtml

P7: “People were living in individual, family individual cells, not exactly houses, not exactly rooms… Anyway there was some level of physical insulation from family to family and so you could say this family is all close contacts and it gives you good conscience to clear other people like neighbours, declare them as neighbours, but not close contacts.”

Technology
P2: “We don’t have a closed examination room... But some refugees are quite smart, so they bring in photos.”

Donations
P3: “We managed to raise money for the cream... we have managed to raise quite a lot, well not a lot, but some money to be able to buy medication.”

“There would be donations of clothes, quite often from the main two warehouses.”

P5: “When I got the donation of ivermectin coming, it was good. I was so happy, I think the person who was leaving me so meds, she didn’t know what happened, I was really quite… I knew that it was so much easier to treat scabies.”

P8: “Our organisation runs on donations so if we did have a donation then we would be able to buy medicines.”

P9: “Fundraising for medicines.”

P10: “We had a stock [medication] from the Netherlands from donations.”

Guidance
P**7**: [Treatment protocol]
“The protocols worked well... The ability to pass on a systematic and well-rehearsed message.”

“One treatment which was once easily explained and where the neighbours could explain to you, and everyone could explain to each other and reinforce the message which we gave every day... We had one treatment which fits into the medical centre and one treatment which was approved by the different governments and that treatment is positive.”

P10: [staff advice/screening documents]
“Screening questionnaire that X staff could use to screen people, which was really important as a lot of volunteers had never seen scabies before... it meant that a lot of people could do the counselling, and tell people to get seen and at the time when the medication was being distributed the one or two doctors who had experienced seeing it would be there, but in the meantime it gave everyone else a tool to find household who had members suffering from scabies.”

For peer review only - http://bmjopen.bmj.com/site/about/guidelines.xhtml

COREQ (COnsolidated criteria for REporting Qualitative research) Checklist

A checklist of items that should be included in reports of qualitative research. You must report the page number in your manuscript where you consider each of the items listed in this checklist. If you have not included this information, either revise your manuscript accordingly before submitting or note N/A.

Topic	Item No.	Guide Questions/Description	Reported on Page No.
**Domain 1: Research team and reflexivity**			
Personal characteristics			
Interviewer/facilitator	1	Which author/s conducted the interview or focus group?	
Credentials	2	What were the researcher's credentials? E.g. PhD, MD	
Occupation	3	What was their occupation at the time of the study?	
Gender	4	Was the researcher male or female?	
Experience and training	5	What experience or training did the researcher have?	
Relationship with participants			
Relationship established	6	Was a relationship established prior to study commencement?	
Participant knowledge of the interviewer	7	What did the participants know about the researcher? e.g. personal goals, reasons for doing the research	
Interviewer characteristics	8	What characteristics were reported about the inter viewer/facilitator? e.g. Bias, assumptions, reasons and interests in the research topic	
**Domain 2: Study design**			
Theoretical framework			
Methodological orientation and Theory	9	What methodological orientation was stated to underpin the study? e.g. grounded theory, discourse analysis, ethnography, phenomenology, content analysis	
Participant selection			
Sampling	10	How were participants selected? e.g. purposive, convenience, consecutive, snowball	
Method of approach	11	How were participants approached? e.g. face-to-face, telephone, mail, email	
Sample size	12	How many participants were in the study?	
Non-participation	13	How many people refused to participate or dropped out? Reasons?	
Setting			
Setting of data collection	14	Where was the data collected? e.g. home, clinic, workplace	
Presence of non-participants	15	Was anyone else present besides the participants and researchers?	
Description of sample	16	What are the important characteristics of the sample? e.g. demographic data, date	
Data collection			
Interview guide	17	Were questions, prompts, guides provided by the authors? Was it pilot tested?	
Repeat interviews	18	Were repeat inter views carried out? If yes, how many?	
Audio/visual recording	19	Did the research use audio or visual recording to collect the data?	
Field notes	20	Were field notes made during and/or after the inter view or focus group?	
Duration	21	What was the duration of the inter views or focus group?	
Data saturation	22	Was data saturation discussed?	
Transcripts returned	23	Were transcripts returned to participants for comment and/or	

For peer review only

Topic	Item No.	Guide Questions/Description	Reported on Page No.
		correction?	
**Domain 3: analysis and findings**			
Data analysis			
Number of data coders	24	How many data coders coded the data?	
Description of the coding tree	25	Did authors provide a description of the coding tree?	
Derivation of themes	26	Were themes identified in advance or derived from the data?	
Software	27	What software, if applicable, was used to manage the data?	
Participant checking	28	Did participants provide feedback on the findings?	
Reporting			
Quotations presented	29	Were participant quotations presented to illustrate the themes/findings? Was each quotation identified? e.g. participant number	
Data and findings consistent	30	Was there consistency between the data presented and the findings?	
Clarity of major themes	31	Were major themes clearly presented in the findings?	
Clarity of minor themes	32	Is there a description of diverse cases or discussion of minor themes?	

Developed from: Tong A, Sainsbury P, Craig J. Consolidated criteria for reporting qualitative research (COREQ): a 32-item checklist for interviews and focus groups. *International Journal for Quality in Health Care*. 2007. Volume 19, Number 6: pp. 349 – 357

Once you have completed this checklist, please save a copy and upload it as part of your submission. DO NOT include this checklist as part of the main manuscript document. It must be uploaded as a separate file.

For peer review only
